# Conserved Secondary Structures in Viral mRNAs

**DOI:** 10.3390/v11050401

**Published:** 2019-04-29

**Authors:** Michael Kiening, Roman Ochsenreiter, Hans-Jörg Hellinger, Thomas Rattei, Ivo Hofacker, Dmitrij Frishman

**Affiliations:** 1 Department of Bioinformatics, Wissenschaftszentrum Weihenstephan, Technische Universität München, Maximus-von-Imhof-Forum 3, D-85354 Freising, Germany; m.kiening@wzw.tum.de; 2 Research Group Bioinformatics and Computational Biology, Faculty of Computer Science, University of Vienna, Währingerstr. 29, 1090 Vienna, Austria; romanoch@tbi.univie.ac.at (R.O.); ivo@tbi.univie.ac.at (I.H.); 3 Department of Theoretical Chemistry, Faculty of Chemistry, University of Vienna, Währingerstrasse 17, 1090 Vienna, Austria; 4 Division of Computational Systems Biology, Department of Microbiology and Ecosystem Science, University of Vienna, Althanstraße 14, 1090 Vienna, Austria; joerg.hellinger@univie.ac.at (H.-J.H.); thomas.rattei@univie.ac.at (T.R.); 5 St. Petersburg State Polytechnic University, St. Petersburg 195251, Russia

**Keywords:** mRNA structure, structure database, secondary structure, viral mRNA, subVOG, structurally related, RNA structure, structurally homogenous, structurally related, mRNA families

## Abstract

RNA secondary structure in untranslated and protein coding regions has been shown to play an important role in regulatory processes and the viral replication cycle. While structures in non-coding regions have been investigated extensively, a thorough overview of the structural repertoire of protein coding mRNAs, especially for viruses, is lacking. Secondary structure prediction of large molecules, such as long mRNAs remains a challenging task, as the contingent of structures a sequence can theoretically fold into grows exponentially with sequence length. We applied a structure prediction pipeline to Viral Orthologous Groups that first identifies the local boundaries of potentially structured regions and subsequently predicts their functional importance. Using this procedure, the orthologous groups were split into structurally homogenous subgroups, which we call subVOGs. This is the first compilation of potentially functional conserved RNA structures in viral coding regions, covering the complete RefSeq viral database. We were able to recover structural elements from previous studies and discovered a variety of novel structured regions. The subVOGs are available through our web resource RNASIV (RNA structure in viruses).

## 1. Introduction

Secondary structures formed in single-stranded mRNA molecules through complementary self-interactions, both in the untranslated (UTR) and coding (CDS) regions of mRNAs, have been implicated in a variety of regulatory functions [1]. For example, riboswitches modulate gene expression through conformational changes in response to various stimuli [2]. Translation initiation, elongation, and termination as well as translation efficiency depend on higher order mRNA secondary structures in non-coding regions [3,4]. CDS hairpins have also been suggested to play a role in the regulation of translation [5], in particular by causing ribosomal stalling and modulating translational efficiency [6]. The relationship between mRNA structure in the CDS and gene expression has been demonstrated both computationally and experimentally [7,8,9,10,11]. In particular, reduced mRNA stability near the start codon has been observed in a wide range of species, probably as a mechanism to facilitate ribosome binding or start codon recognition by initiator-tRNA [12]. Structured elements within CDS directly influence mRNA abundance [13]. Computational studies show that native mRNAs have lower folding energies and are thus more stable than codon-randomized ones [5]. The three mRNA functional domains—5′UTR, CDS, and 3′UTR—form largely independent folding units, while base pairing across domain borders is rare [14]. The ability of viruses to persist in their host in a genus-specific manner is influenced by the interplay between local structural motifs and genome-scale ordered RNA structures (GORS) [15], which impose additional restraints on the RNA sequence space. Evolutionarily conserved local secondary structures have been identified in CDSs [16] and shown to be functional [17]. An indirect indication of the global importance of RNA structures in the coding regions comes from the recent study of Fricke et al. who identified selection favoring specific pairing patterns between synonymous codons within RNA hairpins [18].

Increasing evidence suggests that secondary structural elements in the CDSs of viral RNAs also constitute a previously underappreciated, evolutionarily conserved level of functional organization of viruses. A large number of conserved secondary structural motifs were computationally identified in the Flavivirus genomes [19,20,21], predicted to restrain sequence variability [22] and experimentally shown to regulate important biological processes, such as replication and infection [21]. Multiple secondary structures were described in the coding regions of the (+) sense RNA of the Influenza A virus [23]. Another example is a secondary structural element within the coding region of the Dengue virus type 2, which is essential for its replication [24]. More recently, using a comparative genomics approach, Goz and Tuller identified a large number of potentially functionally important regions in the coding regions of Dengue viruses, in which the RNA folding strength is conserved independently of sequence conservation and compositional bias [25]. Specific regions in the HIV structural genes were reported to be under strong selection for stable secondary structures [26]. Recent research shows that mechanisms of translational control by RNA structures can be shared between viruses and cellular organisms [27].

Given the important role played by RNA structures in shaping the evolutionary dynamics of viruses and modulating their interaction with the host, a large-scale investigation of RNA motifs in viruses would be warranted. However, there are two major challenges that need to be addressed before embarking on such an investigation. First, accurate structure prediction for long RNA molecules, such as mRNAs, is generally out of reach for the existing computational methods. Second, conserved stem-loop structures can only be derived from a collection of high-quality alignments of orthologous viral transcripts, which are difficult to obtain, given the rapid pace of viral evolution and the ensuing poor sequence conservation, even between closely related species.

Here, we propose a computational approach to explore the RNA structurome of the viral coding regions, in which local structure predictions are applied to VOG (Viral Orthologous Groups, http://vogdb.org), the first comprehensive collection of orthologous groups derived for all viral proteins contained in the RefSeq [28] database. We utilize RNALalifold [29] to scan long input sequences for locally optimal secondary structures. The identified structural boundaries are more accurate than those derived from using a sliding window of fixed length. Functional importance of structured regions is assessed by RNAz [30]. We present a novel database, RNASIV (RNA structure in viruses; http://rnasiv.bio.wzw.tum.de), which contains the largest currently available collection of predicted RNA structures in viruses. It provides access to 201,708 viral mRNA sequences clustered into 42,293 structurally homogenous groups and is intended to become a useful tool for exploring structure–function relationships in virus families.

## 2. Materials and Methods

### 2.1. Viral Orthologous Groups (VOGs)

All genome sequences and their annotations were retrieved from the RefSeq viral database release 79 [31] and grouped into phages and non-phages, based on the available taxonomic information. Assemblies containing inconsistently annotated or completely unannotated polyproteins were identified based on the manually curated information provided by ViralZone [32] and excluded from consideration. Phage and non-phage protein sequences were clustered into phage and non-phage preVOGs, using the NCBI’s COG software package with all default settings.

For all phage and non-phage preVOGs, multiple sequence alignments were constructed with Clustal Omega v1.2.4 [33] and used to build HMM-profiles using HMMer 3 [34]. The profiles were subsequently aligned against each other, using HHalign from the HHsuite toolkit [35]. The number of aligned HMM columns was used as an alignment score. All scores for alignments with HHalign probability >85, HHalign *e*-Value < 10^−5^, and more than 70% of aligned columns between the query and the match HMM were stored as an all-against-all matrix. This matrix was clustered into 21,200 VOGs, using the MCL (Markov Clustering) method [36]. Based on the manual inspection of the homogeneity of the protein function descriptions in the resulting clusters, we selected the inflation value of 2.0 for the MCL clustering. For all VOG member proteins, we determined the closest homolog in the UniProt database [37] from BLAST [38] hits with *E*-values better than 10^−5^ and a minimal query coverage of 90%. Functional descriptions of VOGs were automatically derived based on the most frequent protein description found in the UniProt entries or, if not available, in the RefSeq annotation [31]. The complete VOG dataset, which was used in this study, and supplementary files are available for download at http://vogdb.org.

### 2.2. Mapping VOG Sequences to Specific Hosts

**Figure 1:**
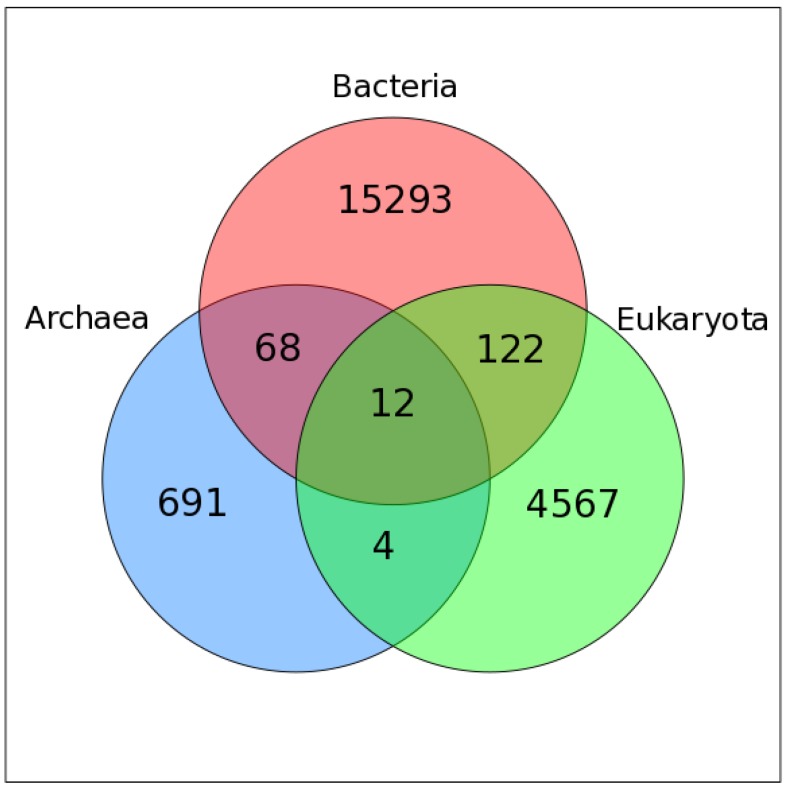
Venn diagram showing the taxonomy of the host organisms within all viral orthologous groups (VOGs). Only those VOGs are included for which host annotation for all viruses is available in the Virus-Host DB.

We used Virus-Host DB [39] to assign host information to VOG proteins. For 20757 VOGs, we were able to map all contained sequences to a specific host, while 428 VOGs contain proteins from at least one viral species for which we could not find host annotation. Most VOGs include viruses infecting hosts from only one domain of life, i.e., bacteria (~72%), eukaryotes (~22%), or archaea (4%), while only 2% of VOGs are taxonomically mixed (Figure 1). Only 12 VOGs contain viruses that infect hosts from all three domains of life. The VOG sizes range from 15 proteins of 12 distinct species, up to 265 proteins belonging to 261 different species (on average, 104 proteins from 95 different species). These VOGs mostly harbor highly conserved core enzymes of double-stranded DNA viruses, such as kinases, ligases, methylases, helicases, hydrolases, and synthases [40]. The other two VOGs additionally contain proteins from viruses belonging to the order of Caudovirales, which belong to the bacteriophages, which are not classified as double-stranded DNA viruses, according to the NCBI taxonomy. We excluded from consideration 15 VOGs containing satellite viruses infecting other viruses.

### 2.3. Distance Trees of VOG Proteins

**Figure 2:**
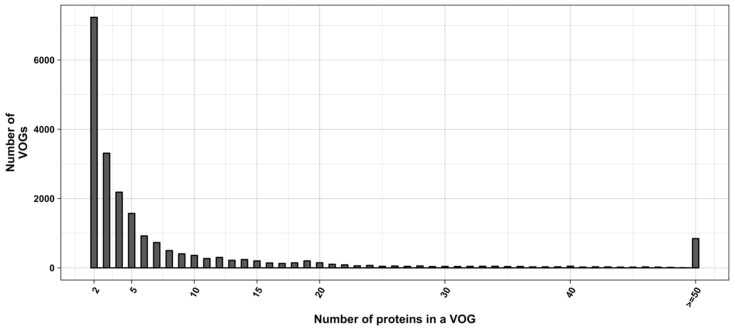
Distribution of VOG sizes.

Expectedly, we found that RNA structure conservation within VOGs decreases with increasing VOG size. Most VOGs (66%) consist of at least three sequences (size distribution shown in Figure 2) and can therefore potentially be split into smaller groups containing structures that are not conserved across the entire VOG. We therefore utilized distance trees derived by the neighbor-joining algorithm [41] to identify structurally homogeneous subsets of VOGs (subVOGs). All-against-all pairwise alignments of protein sequences were calculated using Clustal Omega and then converted to the nucleotide alphabet. The distance matrices were derived from pairwise sequence identity values, and the trees were created from the matrices using neighbor joining, as implemented in the BioPerl toolkit [42]. The inner nodes of the sequence trees represent possible subVOG candidates, potentially containing structurally homogenous sequences.

### 2.4. Structure Prediction and subVOG Assignment

In order to assess the amount of structural RNA conservation present in subVOG candidates, multiple sequence alignments (MSAs) of proteins were calculated for each inner node of the distance trees and converted to the nucleotide alphabet. The RefSeq nucleotide and protein sequences were obtained from the VOGDB. We then employed RNALalifold from the ViennaRNA package [29], with default parameters, to determine the boundaries of locally stable structures within each MSA, and realigned these local regions using mLocARNA [43]. MLocARNA produces structure-guided multiple sequence alignments, using an adapted version of the Sankoff algorithm. The significance and conservation of the found structures was assessed with RNAz [30]. This procedure is simpler and arguably more accurate than the usual approach of applying RNAz to the entire MSA within a sliding window. RNAz classifies fragments of an MSA pre-selected by RNALalifold as containing or not containing a functional RNA secondary structural element. Realignment with mLocARNA significantly increases the precision of RNAz [30]. As no sequence of a potential subVOG can be regarded as a reference sequence, the option “no reference” was used for the subsequent RNAz analysis. The RNAz method uses the RNAfold algorithm from the ViennaRNA package to calculate secondary structures and the corresponding minimum free energy (MFE) for each individual RNA sequence in the alignment. In addition, for each aligned sequence set, RNAz calculates a consensus secondary structure and its MFE using the RNAalifold algorithm. RNAz assumes that conserved and thermodynamically stable structures are functional, in which case it outputs “RNA”. Otherwise, it outputs “OTHER”. For this purpose, a class probability value, combining all information on an input alignment is calculated. We used a stringent threshold of 0.9 (default 0.5) for the class probability value, which is recommended for finding high confidence structures [30]. Subsequently, the trees were scanned for subtrees containing at least one conserved structural element, that is, predicted to be functional, and the largest subtrees were designated as structurally homogenous subVOGs. We found that sequences that are only distantly related according to the neighbor-joining tree may still share conserved RNA structures. In order to account for structure-level relationships between sequences, we built covariance models for all conserved structures found within subVOGs, using the tool cmbuild from the infernal package [44], and used them to search against all sequences in the entire VOG database.

### 2.5. mRNA Stability

Following Tuller et al. [45] and Faure et al. [46], we employed RNAfold to calculate the folding energy of the most and the least stable 30-nucleotide segment of mRNAs (ΔGmin and ΔGmax, respectively), as well as the average folding energy of all possible 30 nucleotide segments (ΔGmean). Faure et al. investigated the effect of mRNA stability on the translation rate and protein folding. During translation, the ribosome sequentially unfolds parts of the mRNA. These parts are typically 30 nucleotides long, which explains the choice of segment length in Faure et al. As this procedure does not take into account the actual boundaries of local structures, but rather limits all structures to the size of 30 nucleotides, we additionally calculated the three energy values for all local optimal structures found with RNALfold.

### 2.6. mRNA Structures and Protein Function

We investigated the relationship between protein function, described in terms of gene ontology (GO) annotation [47], and mRNA structures. Instead of using the global folding energy for classifying mRNAs as highly or lowly structured [48], we considered structural coverage—the portion of an mRNA covered by functional and conserved structures. GO terms for all VOG proteins were downloaded using QuickGO [49], where available. Based on the Evidence & Conclusion Ontology (ECO) evidence codes [50], two separate datasets were created: (i) Proteins annotated by manually or experimentally derived GO terms (ECO evidence codes: ECO:0000352, ECO:0000269), and (ii) proteins annotated by GO terms with any evidence codes. To find out whether mRNAs of proteins with certain functions tend to harbor more or fewer structures, we pooled together functionally similar GO terms with the average structural coverage of their corresponding mRNAs, using Revigo [51]. Revigo uses a semantic similarity measure to group similar GO terms together, which results in a concise list of distinct functions. To perform this analysis, we calculated the average structural coverage of all subVOG mRNAs with available GO annotation. For the experimental dataset we allowed a coverage value to be associated with a GO term if more than 50% of the sequences in a particular subVOG were annotated with this term. Within the dataset based on all evidence codes, we only allowed GO terms shared by all sequences of a subVOG. We only used mRNAs that were clustered into a subVOG. For sequences that were not part of any subVOG, we did not find conserved structures, although this does not necessarily mean that the mRNA did not contain functional structures. The distributions of standard deviations of the structural coverage values were compared within the actual and randomly generated Revigo clusters. Randomization was performed 1000 times by preserving the size of the clusters and filling them with randomly chosen GO terms.

## 3. Results

### 3.1. Overview of the Study

**Figure 3:**
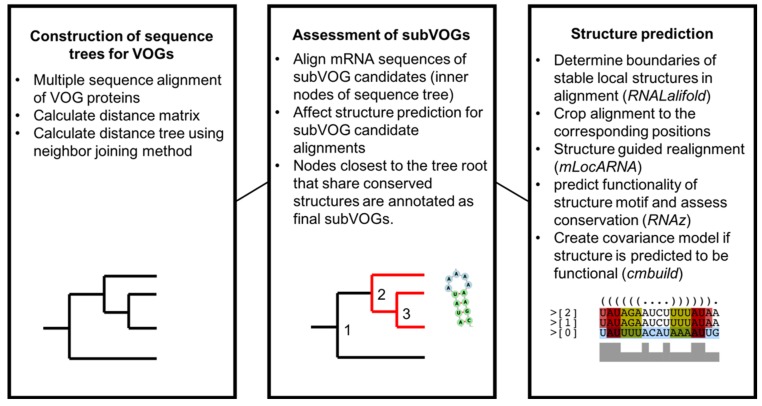
Overview of the analysis of conserved RNA structures in VOGs.

A graphical overview of the study is given in Figure 3. In a first step, we created distance trees for all protein sequences contained in each VOG, using the neighbor joining method, as described in Materials and Methods. All sequences of the inner nodes of each tree, representing potential subVOGs, were multiply aligned, converted to the nucleotide alphabet and processed with RNALalifold to obtain all potentially conserved local optimal structures. Each part of the alignment covering a potential structure was then realigned with the structure-guided alignment method mLocARNA and checked for functionality using RNAz. The use of structure-guided alignments as input for RNAz improves the performance, compared to pure sequence-based alignments [30]. The tree nodes containing the most sequences that yielded conserved structures were taken as final subVOGs. For all obtained subVOG structures, we computed covariance models that could be used to search for similar structures in future research.

### 3.2. Structure Conservation in VOGs

**Figure 4:**
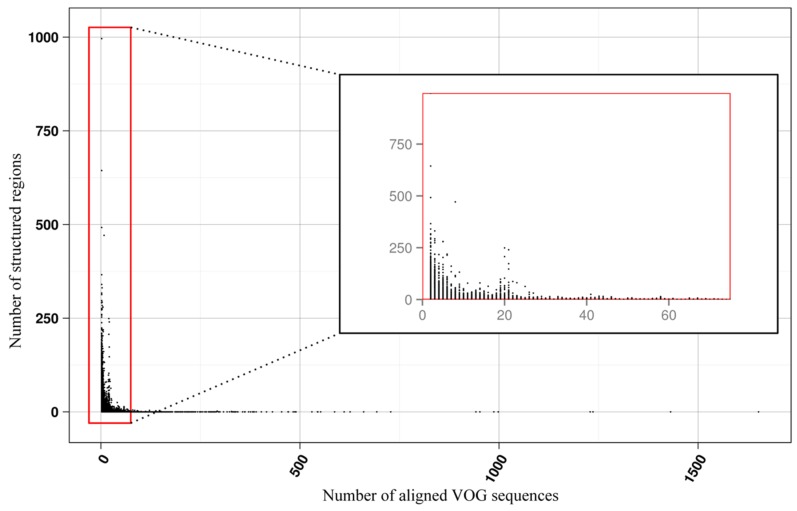
Number of local RNA structures as a function of VOG size.

**Figure 5:**
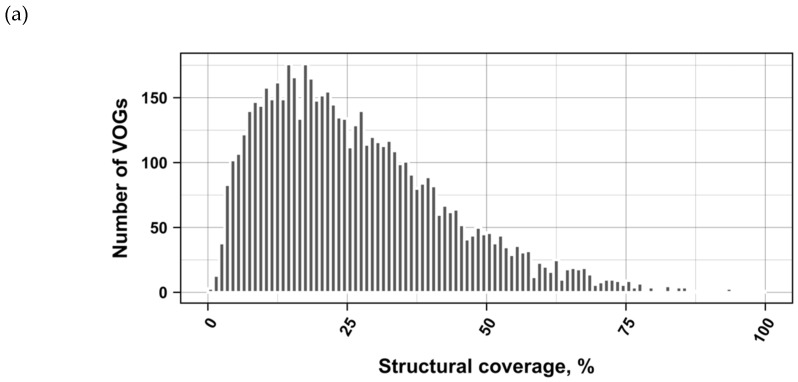

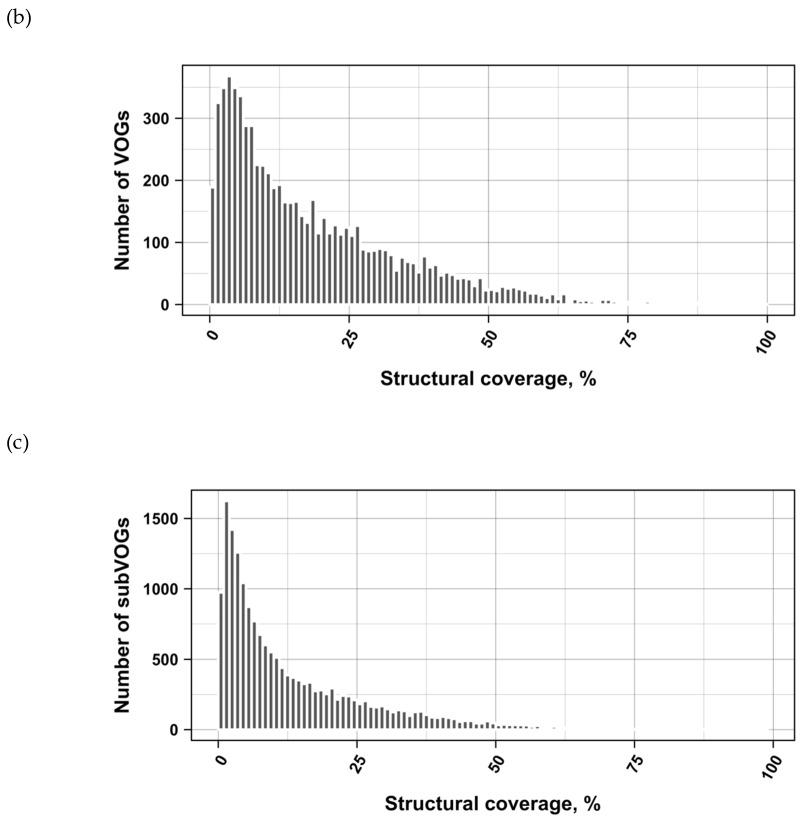
Coverage of VOG alignments by local optimal RNA structures. (**a**) VOGs with two sequences. (**b**) VOGs with more than two sequences, in which structures are conserved across all sequences. (**c**) subVOGs. VOGs that did not contain conserved structures, even after splitting into subVOGs, are not shown.

**Figure 6:**
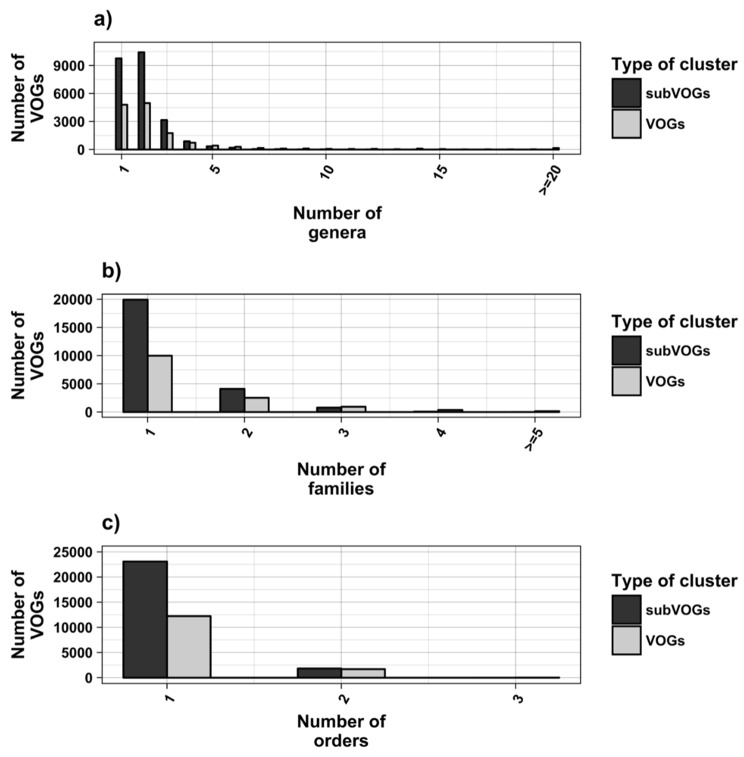
Taxonomic distribution of proteins in VOGs (with more than two sequences) and subVOGs.

The current release of the VOG database, derived from the RefSeq release 77, contains 21,200 VOGs, composed of 251,796 proteins from 6252 phages and eukaryotic viruses (Figure S1). Protein sequences in each VOG were aligned by Clustal Omega, converted to the nucleotide alphabet, and used as input for RNA structure prediction by RNALalifold. As seen in Figure 4, the number of local optimal structures conserved within entire VOGs decreases quickly with the number of aligned sequences, which may in part be the consequence of poor multiple alignment quality in large sets of sequences. Indeed, we found that proteins in smaller VOGs tend to be more closely related (Figure S2). To exclude structures found due to sequence conservation only, the potential functionality of structures was verified with RNAz. However, even those VOGs that only consist of a few sequences do not always contain conserved structures. There are 7232 VOGs with exactly two sequences, and for 1237 of these, we could not find any conserved structures. The remaining 5995 VOGs of size two had an average structural coverage of approximately 25% (Figure 5a). Out of the 13,968 VOGs with more than two sequences, 7238 VOGs were predicted to contain RNA structures conserved across the entire VOG, with an average structural coverage of approximately 18% (Figure 5b). These contain between 3 and 96 sequences, with an average of 6. On average, VOGs contain sequences from three different genera, mostly belonging to the same taxonomic family and thus also to the same order (Figure 6a–c). The 25 most diverse VOGs contain sequences from three different orders and up to 19 taxonomic families. On average, a VOG contains mRNAs from viruses that infect hosts from four different genera, belonging to three different taxonomic families and two orders. The VOG with the highest host diversity corresponds to 209 different host genera from 114 families and 64 orders.

### 3.3. Structure Conservation in subVOGs

**Figure 7:**
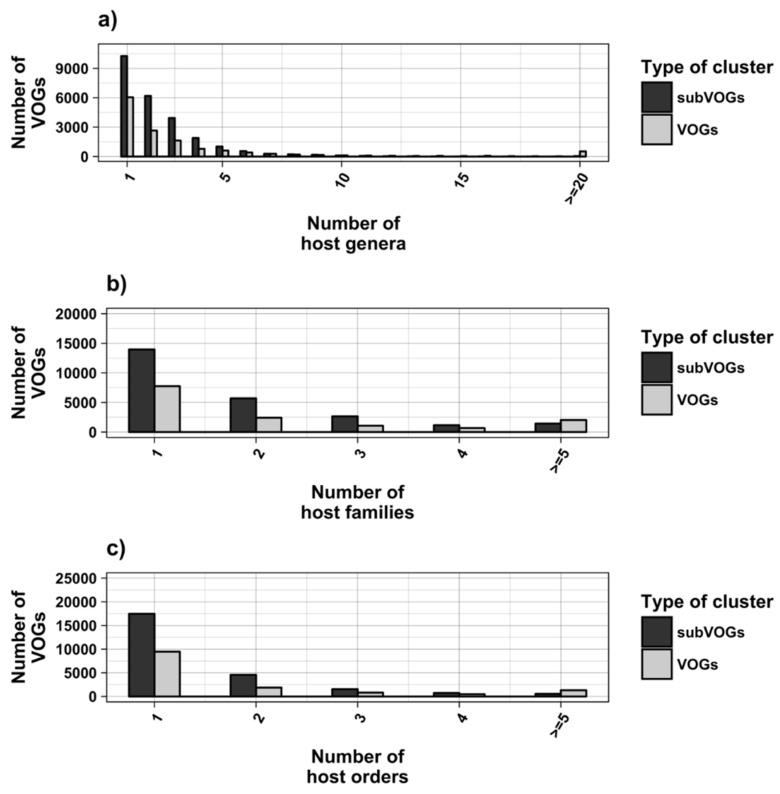
Taxonomic distribution of hosts in VOGs (with more than two sequences) and subVOGs.

**Figure 8:**
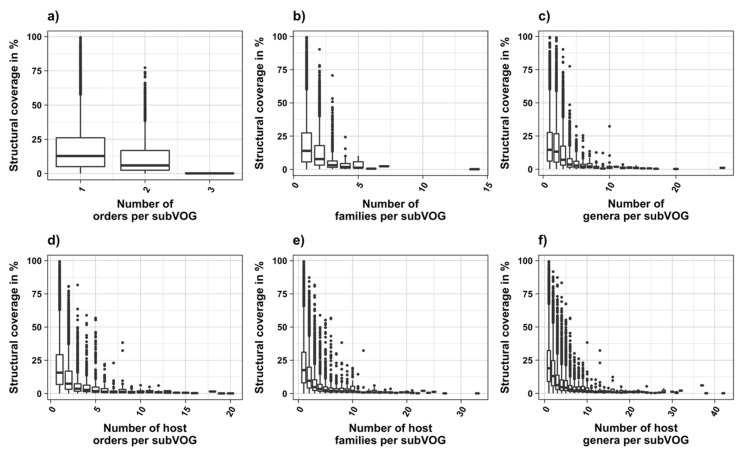
Structural coverage as a function of the taxonomic variety of subVOGs and their host organisms.

**Figure 9:**
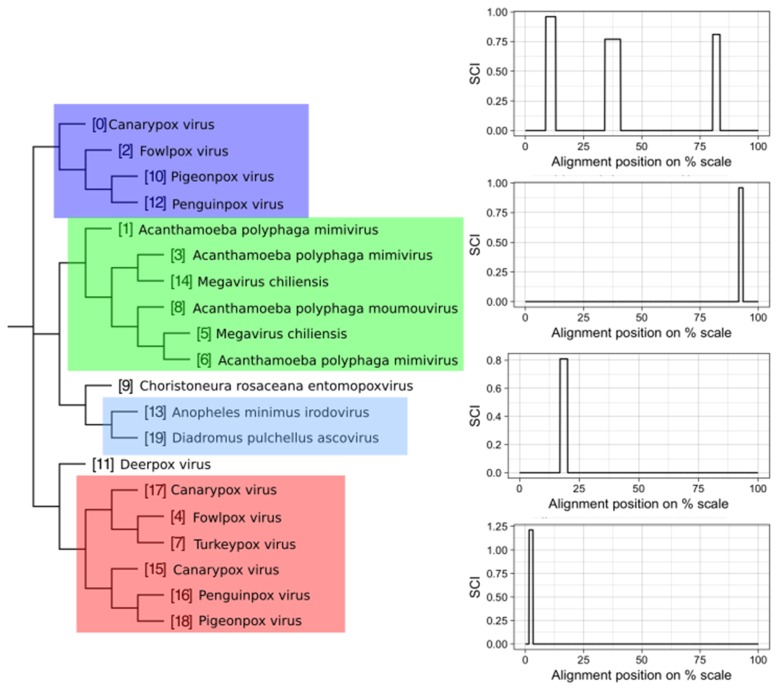
Example of a VOG split into structurally homogenous subVOGs. Shown is the VOG 00052 containing 20 mRNAs, encoding for Kila-N domain proteins, from 12 virus species. On the left, the neighbor-joining tree based on the pairwise sequence identity between the protein sequences is shown. Colored boxes indicate subVOGs, within which conserved structures were predicted. The tree nodes outside colored boxes did not yield any conserved structures. On the right, the structure conservation index (SCI) (black line for each subVOG alignment) is plotted against the alignment position on the percentage scale. Plots are ordered according to the subVOG position in the tree.

We attempted to subdivide 6730 VOGs with more than two sequences and without conserved structures into structurally homogeneous subsets, which we call subVOGs, using phylogenetic trees derived by the neighbor-joining method. This procedure resulted in 17,678 subVOGs with an average structural coverage of approximately 13% (Figure 5c). The average number of genera per subVOG is 2 and the most diverse of them contains sequences from three orders and 14 families. A subVOG contains on average sequences that infect two different host genera, and the most diverse subVOG infects hosts of 42 different genera, belonging to 33 families and 20 different orders (Figure 7a–c). Thus, unsurprisingly, subVOGs, which constitute subsets of full VOGs with increased structural homogeneity, exhibit a reduced taxonomic spread, both of the viruses they contain and their hosts. A large fraction of subVOGs (63%) contains sequences from more than one genus and 21% contain sequences from more than one family. The structural coverage of subVOGs, i.e., the fraction of alignment positions that are located within conserved RNA structures, decreases with increasing taxonomic diversity of the viruses and their hosts (Figure 8). An example that demonstrates the reduction of taxonomic spread between a VOG and its corresponding subVOGs is given in Figure 9. Here, the VOG 00052, which contains 20 proteins from 12 different virus species belonging to 4 different taxonomic families, was split into four structurally homogenous subVOGs. Two of the four subVOGs consist of mRNAs belonging to the genus Avipoxvirus from the family Poxviridae, the third subVOG contains sequences from the family Mimiviridae, and the fourth subVOG consists of two mRNAs belonging to viruses from two different taxonomic families, the Ascoviridae and the Iridoviridae. For two mRNAs, we could not find structures conserved in any of the other VOG members and they are therefore not part of any subVOG.

**Figure 10:**
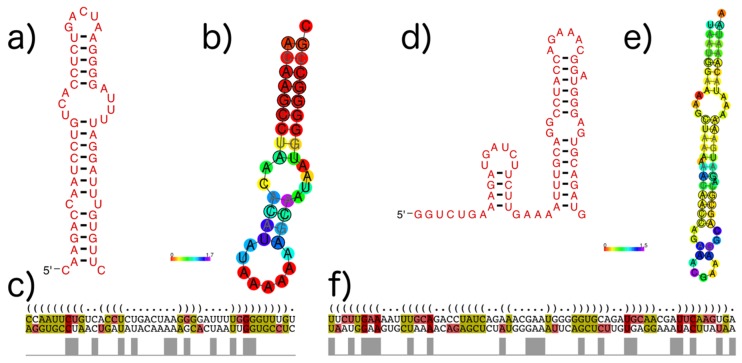
Structures found in Influenza A and B mRNAs encoding the matrix protein (VOG11160). Colors in MSA pictures encode compensatory mutations supporting the consensus structure. Red marks pairs with no sequence variation; ochre, green, turquoise, blue, and violet mark pairs with 2, 3, 4, 5, and 6 different types of pairs, respectively. (**a**) The second of the two consecutive stem loops of the structure proposed by Jiang et al. [52], covering positions 147–192, visualized with R2R [54]; (**b**) The predicted conserved consensus structure for nucleotides 148–188 supports the second hairpin loop of the model of Jiang et al., shown in (**a**). Colors encode the positional entropy; (**c**) Structure-guided alignment and dot bracket structure notation for the consensus structure shown in (**a**). The upper sequence corresponds to Influenza A and the lower sequence to Influenza B; (**d**) Shown are two consecutive hairpin loops for nucleotide positions 682 to 744, proposed by Moss et al. [23], visualized with R2R; (**e**) The predicted conserved structure for nucleotides 697–758 partly supports the model shown in (**e**). Colors encode the positional entropy; (**f**) Structure-guided alignment and dot bracket notation for the consensus structure shown in (**e**). The upper sequence corresponds to Influenza A and the lower sequence to Influenza B.

As an example, Figure 10 shows the subVOG 1 of VOG11160, which contains two mRNAs encoding the matrix protein 1 from the Influenza A virus (H3N2) and the Influenza B virus. There are three RNA structural motifs described in the literature for the Influenza A mRNA. Nucleotides 105 to 192 form either a multibranch structure, according to Moss et al. [23] and Jiang et al. [52], or a double hairpin structure, proposed by Jiang et al. [52]. Two consecutive stem-loop structures are formed from position 682 to 744, according to Moss et al. [23]. Despite the sequences’ dissimilarity between Influenza A and B, both motifs are partly conserved, according to our RNAz analysis of the corresponding subVOG (Figure 10). Our analysis supports the second hairpin loop from the double hairpin structure, described by Jiang et al. (Figure 10a–c). From the second motif, proposed by Moss et al., we also found that the second hairpin structure was partly conserved (Figure 10d–e). The consensus structure of the first motif has a high structure conservation index (SCI) of 0.78, although the part of the alignment covering the structure has a low pairwise identity of 29%. The second motif has an SCI of 0.58 and a pairwise identity of 32%. Our analysis also revealed three further conserved stem-loop structures—position 346 to 369, 438 to 483, and 654 to 674, with SCIs and mPIDs of 0.81 and 29%, 0.66 and 48%, and 0.65 and 33%, respectively.

A recent study of secondary structures in alphaviruses by Kutchko et al. revealed that Sindbis virus mRNAs harbor many functional structures, but they are poorly conserved in the closely related Venezuelan equine encephalitis virus [53]. The corresponding subVOG containing mRNAs coding for the non-structural protein 1 includes orthologous mRNAs from 12 further alphaviruses. We identified three short structures that are conserved in all of the contained species and overlap with the functional structures described by Kutchko et al., while all other structures reported by Kutchko et al. are indeed poorly conserved in further Alphavirus species. 

**Figure 11:**
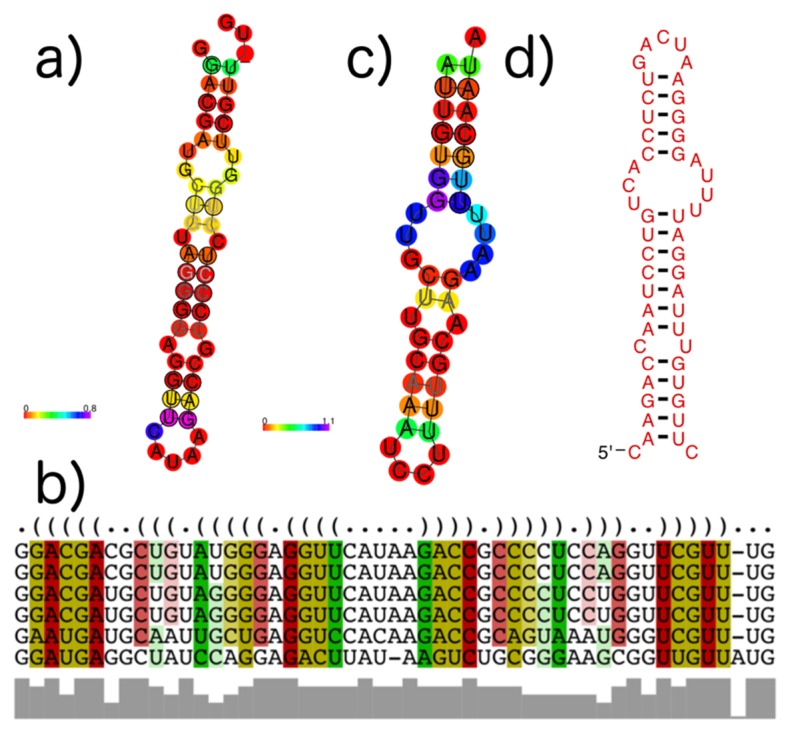
Example structures that were identified within subVOGs. (**a**) Structural annotation of the subVOG 30, belonging to VOG00029, which contains six mRNAs encoding a replicase protein of different Tobamovirus species. Consensus structure visualized by RNAalifold. Colors encode the positional entropy; (**b**) Structure-guided MSA and consensus structure in dot bracket notation corresponding to consensus structure shown in (**a**). Colors encode compensatory mutations supporting the consensus structure. Red marks pairs with no sequence variation; ochre, green, turquoise, blue, and violet mark pairs with 2, 3, 4, 5, and 6 different types of pairs, respectively; (**c**) Consensus structure of subVOG 64 from VOG00003, which contains four mRNAs coding for a p28-like protein of different alphabaculoviruses; (**d**) Structure found in a Heliothis virescens ascovirus 3e, by covariance model search of the structure shown in (**c**), using cmsearch in the entire sequence space of all VOGs.

An example of a subVOG in which structures are conserved across mRNAs from different taxonomic families is given in Figure 11. Shown is a subVOG containing proteins from two mosaic viruses (Maracuja mosaic virus, Tobacco mosaic virus), the Bell pepper mottle virus, and the Odontoglossum ringspot virus (Figure 11a,b). The proteins are classified as replicases and RNA polymerases. The subVOG contains overall 15 locally conserved structured regions. Figure 11 shows the region covering alignment positions 4766 to 4815. The alignment covering this structure has an mPID of 72% and the structures are conserved with an SCI of 0.9.

Overall, we subdivided 21,200 VOGs containing, on average, 11 proteins (233,380 in total) into a total of 42,293 subVOGs, containing, on average, five mRNAs (201,708 in total) and three structured regions (147,087 in total). The VOGs with more than two sequences that had to be split up contain, on average, four subVOGs.

### 3.4. subVOG Covariance Models

We built covariance models for all structures found within subVOGs and, using cmsearch, found that in many cases, structures are conserved between different subVOGs and even between different VOGs. In most cases, this was due to a shared sequence domain. For example, the subVOG 64 from VOG00003 harbors four mRNA sequences from different nucleopolyhedroviruses, belonging to the family Baculoviridae. This subVOG was predicted to contain four conserved structures. One of these structures is a highly conserved stem-loop structure (Figure 11c). This structure can also be found in an mRNA of Heliothis virescens ascovirus 3e, belonging to the family Ascoviridae, which is part of VOG01276 (Figure 11d). The two structures are highly conserved with an SCI close to 1, although they are part of different VOGs and belong to mRNAs of different virus families. The alignment of the corresponding proteins revealed that these sequences share a common domain, but the sequence similarity is below the inclusion threshold of the VOG pipeline (Figure S3).

### 3.5. mRNA Stability and Length

**Figure 12:**
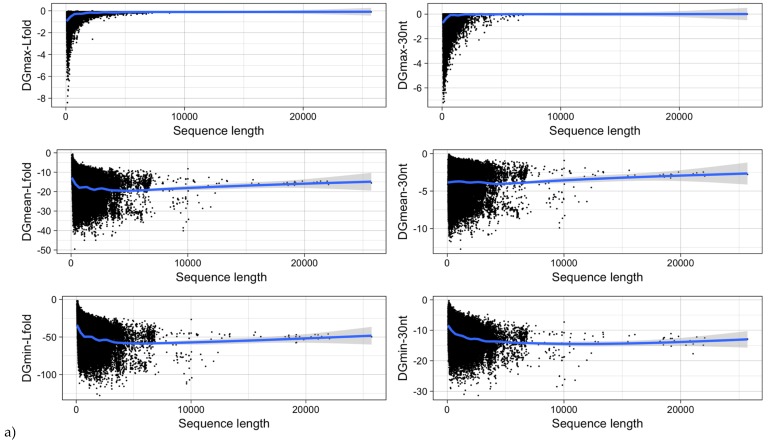

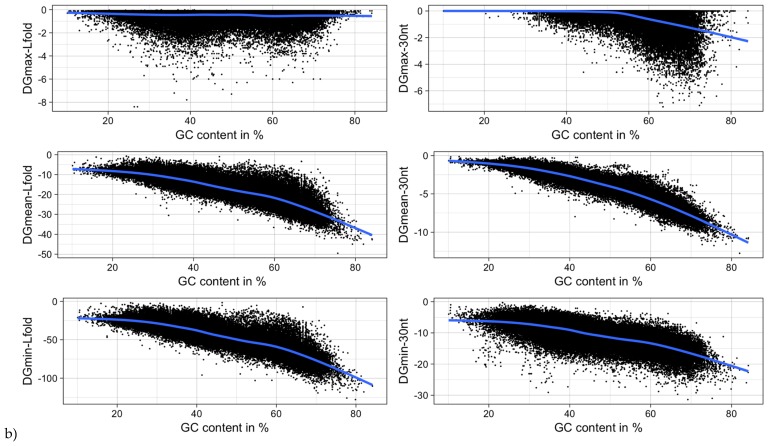
MRNA folding energy as a function of (**a**) sequence length and (**b**) GC-content. DGmin: Minimum folding energy of either all possible 30-nucleotide windows of a sequence or all found local optimal structures using RNALfold. DGmean and DGmax: Mean and maximum of all windows, respectively.

**Figure 13:**
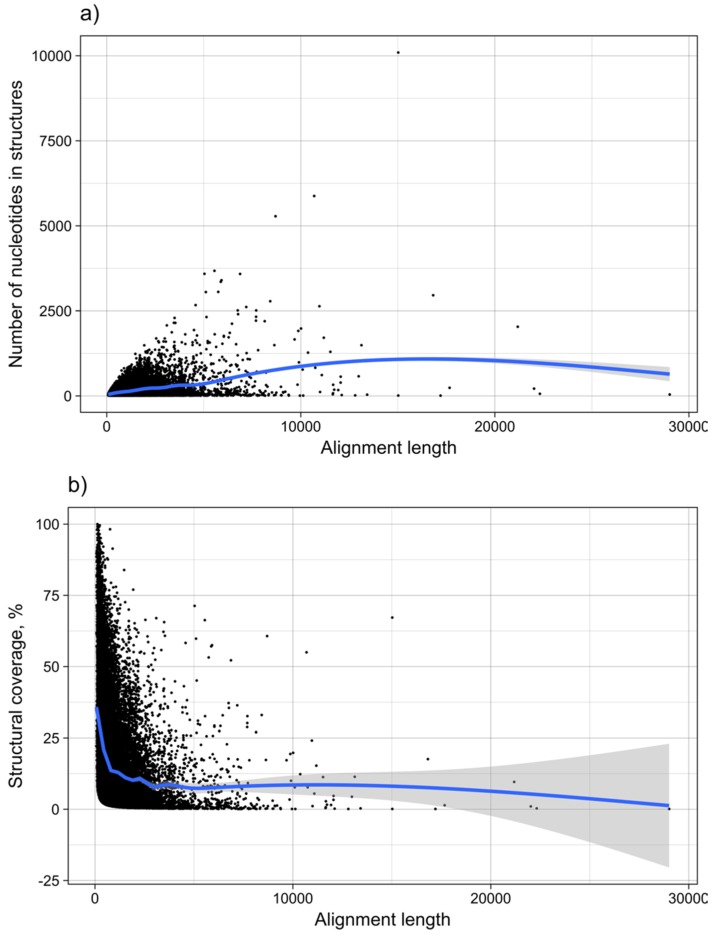
MRNA structure as a function of length. The graph shows the dependence of (**a**) the number of nucleotides within structures predicted to be functional, and (**b**) the structural coverage of the mRNAs in %, from the total length of mRNAs. Each point corresponds to one subVOG.

It was shown for a number of eukaryotic and prokaryotic organisms that longer mRNAs exhibit more stable RNA structures, which allows for more efficient control of co-translational protein folding [45,46]. In our dataset of viral mRNA sequences, we also found a correlation between the free energy of the most stable 30-nucleotide segment of an mRNA (ΔGmin) and mRNA length (Pearson correlation coefficient −0.27; from here on referred to as Pearson’s *r*), but no correlation between the average energy of all possible 30-nucleotide windows (ΔGmean) and mRNA length (Table 1, Figure 12a). We additionally calculated the free energy of the most and least stable local optimal segment found by RNALalifold as well as the mean energy of all found RNALalifold segments, and obtained Pearson’s r values of −0.25, −0.07, and 0.29 respectively. The Pearson’s r of folding energy and GC content lies between −0.5 for ΔGmax and −0.94 for ΔGmean (Table 1, Figure 12b). The number of bases that are within functional structures is positively correlated with the alignment length of subVOGS (Pearson’s r 0.40, *p*-value < 2.2^−16^), while this correlation becomes negative when considering the percentage of bases within structures (structural coverage) instead of the absolute value (Pearson’s r −0.27, *p*-value < 2.2^−16^) (Figure 13). In other words, longer mRNAs harbor more or longer structured regions, but at the same time, the percentage of positions in functional structures decreases with increasing length. The only explanation for this effect that we can think of is that there is a certain number of structured elements needed for regulatory functions, which is largely independent of the mRNA length. As expected (see Figure 8), there is a weak but significant negative correlation (Pearson’s r −0.23, *p*-value < 2.2^−16^) between structural coverage and the number of sequences in the MSA, with more taxonomically diverse alignments containing fewer conserved structures. 

### 3.6. mRNA Structures and Protein Function

We analyzed the relationship between protein function and mRNA structure in viral subVOGs by comparing RNA structural coverage with gene ontology (GO) annotation. Using the QuickGO database, we identified a total of 814 VOG proteins that are manually or experimentally annotated (according to ECO evidence codes, as described in Materials and Methods) with GO terms, of which 727 are part of a subVOG, and thus harbor conserved structures according to our analysis. (For the sake of completeness, we also performed the same analysis for all GO annotated proteins, without regard for the annotation evidence codes, see Table S2). For each individual GO term, we only considered the structural coverage of mRNA sequences if that term was assigned to more than 50% of the proteins in a given subVOG. This resulted in 106 GO terms from the biological process sub-ontology and 17 terms from the molecular function sub-ontology. Note that no GO terms from the cellular component sub-ontology satisfied the criteria explained above. 

**Figure 14:**
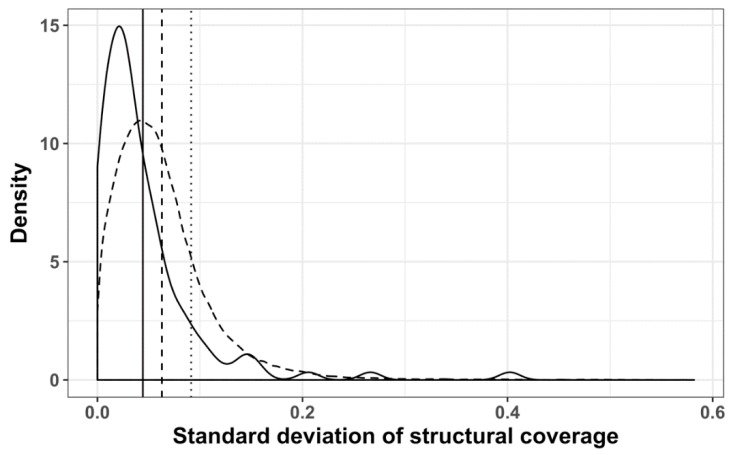
Distribution of standard deviations of mRNA structural coverage, mapped to GO-terms: Clustered with Revigo (solid line); randomized Revigo clusters (dashed line); not clustered (dotted line); vertical lines represent the mean of the corresponding dataset.

Using Revigo, we derived 70 functionally similar groups of GO terms, with 57 belonging to the biological process ontology and 13 to the function sub-ontology (Table S1). The resulting GO term groups were subdivided into three categories, according to the average structural coverage of the corresponding subVOGs: Low structural coverage (up to 10%), medium structural coverage (up to 20%), and high structural coverage (more than 20%). We found that the standard deviation of the structural coverage values within the Revigo clusters was significantly smaller (Wilcoxon test *p*-value 1.068^−10^), compared to randomized clusters (Figure 14). In other words, our findings suggest that mRNAs encoding the proteins with coherent functions tend to exhibit a similar structural coverage.

These findings are in line with the previous study by Vandivier et al. who found that transcripts in *Arabidopsis thaliana* with similar levels of secondary structure in their untranslated and coding regions tend to encode functionally similar proteins [48]. Likewise, Wang et al. also identified GO terms associated with highly or lowly folded mRNAs in yeast [55]. Four of the GO terms associated with highly structured mRNAs, according to Wang et al. (regulation of translation, posttranscriptional regulation of gene expression, regulation of cellular protein metabolic process, and cellular nitrogen compound biosynthetic process), correspond to highly structured viral mRNAs in our data. At the same time, none of the GO terms corresponding to lowly structured yeast mRNAs according to Wang et al. were enriched in our results. On the other hand, Fan Li et al. found that *Arabidopsis thaliana* mRNAs related to “regulation of transcription” were structurally unstable [56], while we found that mRNAs encoding the proteins related to “viral transcription” do harbor conserved RNA structures. We also found virus-specific trends not previously observed for cellular proteins, such as the high structure of viral mRNAs coding for proteins that regulate replication and transcription, suppression by viruses of host translation, or modulation by viruses of host process (Table S1). It has been reported that mRNA folding strength influences the efficiency of gene expression and that mRNAs encoding abundant proteins generally tend to be more structured [57]. In the future, once RNA-seq data for a sufficient number of viral genes becomes available, it will be interesting to investigate whether functional coherence between mRNAs with similar structural coverage is, at least in part, caused by similar expression levels.

### 3.7. subVOG Online Resource

Structurally homogenous subVOGs can be accessed online (http://rnasiv.bio.wzw.tum.de) through two entry points: “Browse by VOG” and “Browse by taxonomy”. The first option is a list of all VOGs, together with the consensus description of their constituent proteins. The list can be filtered with a keyword search and links to the corresponding subVOGs of each VOG are provided. The second option is an expandable taxonomic tree, based on the NCBI taxonomy [58], which allows navigation to the viral species of interest. For each species, mRNA sequences are provided, if available, interlinked to the corresponding subVOGs. Tree nodes containing only mRNAs that are not part of any subVOG are colored grey. Each subVOG contains at least two sequences that share at least one structural element predicted to be functional. If a species of interest is not contained in the subVOG database, the taxonomy tree makes it possible to find the taxonomically closest species. Web pages describing individual subVOGs contain four parts:

(i) General information, i.e., number of mRNAs in the subVOG, the number of proteins and species in the parent VOG, as well as a consensus functional description;
(ii) Information on conserved structures among the subVOG sequences. A plot outlining the SCI for each column of the subVOG MSA gives a brief overview over the structure of the subVOG members. Also provided is a table that shows a list of all structures found, including the corresponding values of SCI, mPID, and the GC content. The consensus structure can also be visualized by Forna, and a covariance model is provided, which can be used to search for similar structures. Additionally, the RNAz results for each individual structured region can be accessed, including structure visualization, dot plots, and the local structure-guided alignments;
(iii) The global MSA for the subVOG sequences. Alignment columns colored in blue correspond to the structured regions described in the previous section. The alignment is visualized with the javascript library MSAviewer [59], which is based on Jalview [60];
(iv) The list of subVOG members, including protein names, descriptions, and taxonomy. For each protein, a link to the RefSEQ entry is provided, as well as the amino acid and nucleotide sequences. The leftmost column of the list contains a checkbox for each subVOG member, which can be used to build a subset of members and analyze the RNA structures shared by these.

## 4. Discussion

In this work we set out to create a possibly complete census of conserved RNA secondary structures in the coding regions of viruses and to shed light on their biological role. Using sequence comparison and structure prediction methods, we derived structurally homogenous groups of viral mRNAs from subsets of viral orthologous groups (VOGs), which we call subVOGs. We identified a total of 147,087 conserved structures in 42,293 subVOGs, which we make accessible through our database RNASIV (RNA Structures in Viruses). On average, subVOGs contain three structured regions and their structural homogeneity decreases with increasing taxonomic diversity of the viruses and their hosts. We found that 63% of all subVOGs contain mRNAs from at least two genera and 21% from more than one taxonomic family. In line with the previous studies on cellular organisms, we confirm that, in viruses, longer mRNAs tend to contain more stable structures. However, the number of structures grows only slowly with length, which implies that there is a certain minimum amount of structures required to maintain regulatory functions and control protein folding. MRNAs annotated with similar GO terms tend to have a similar structural coverage, hinting at possible commonalities in the regulatory mechanisms of functionally related proteins. It is hoped that RNASIV will be a useful resource for exploring the structure–function relationships in viral mRNAs.

## Figures and Tables

**Table 1:** Pearson correlation between alignment length or GC-content and the minimum (ΔGmin), maximum (ΔGmax), or mean (ΔGmean) folding energy of either all possible 30-nucleotide long-sequence windows or all local optimal structures found with RNALfold, of all mRNAs in our data set. *P*-values are given in parentheses.

Type of ΔG	Pearson Correlation Coefficient	
	ΔG vs. Sequence Length	ΔG vs. GC-Content
ΔGmin	−0.27 (<2.2^−16^)	−0.73 (<2.2^−16^)
ΔGmean	0.004 (0.1655)	−0.94 (<2.2^−16^)
ΔGmax	0.17 (<2.2^−16^)	−0.50 (<2.2^−16^)
ΔGmin (RNALfold)	−0.24 (<2.2^−16^)	−0.86 (<2.2^−16^)
ΔGmean (RNALfold)	−0.16 (<2.2^−16^)	−0.86 (<2.2^−16^)
ΔGmax (RNALfold)	0.29 (<2.2^−16^)	−0.07 (<2.2^−16^)

## Supplementary Materials

The following are available online at https://www.mdpi.com/1999-4915/11/5/401/s1: Figure S1: Virus lineages included in the VOGs; Figure S2: Mean pairwise sequence identity of VOG alignments as a function of VOG size; Figure S3: Sequence Alignment of a protein from Heliothis virescens ascovirus 3e and proteins belonging to the mRNAs of subVOG 64 of VOG00003; Table S1: Clustering of GO terms of subVOG proteins and the average structural coverage of their corresponding mRNAs; Table S2: Clustering of GO terms of subVOG proteins and the average structural coverage of their corresponding mRNAs (regardless of GO evidence codes).

## References

[1] Bevilacqua P.C., Blose J.M. (2008). Structures, kinetics, thermodynamics, and biological functions of RNA hairpins. Annu. Rev. Phys. Chem..

[2] Serganov A., Patel D.J. (2007). Ribozymes, riboswitches and beyond: Regulation of gene expression without proteins. Nat. Rev. Genet..

[3] Gray N.K., Hentze M.W. (1994). Regulation of protein synthesis by mRNA structure. Mol. Biol. Rep..

[4] Kozak M. (2005). Regulation of translation via mRNA structure in prokaryotes and eukaryotes. Gene.

[5] Katz L., Burge C.B. (2003). Widespread selection for local RNA secondary structure in coding regions of bacterial genes. Genome Res..

[6] Mortimer S.A., Kidwell M.A., Doudna J.A. (2014). Insights into RNA structure and function from genome-wide studies. Nat. Rev. Genet..

[7] Kudla G., Murray A.W., Tollervey D., Plotkin J.B. (2009). Coding-sequence determinants of gene expression in Escherichia coli. Science.

[8] Duan J., Wainwright M.S., Comeron J.M., Saitou N., Sanders A.R., Gelernter J., Gejman P.V. (2003). Synonymous mutations in the human dopamine receptor D2 (DRD2) affect mRNA stability and synthesis of the receptor. Hum. Mol. Genet..

[9] Ilyinskii P.O., Schmidt T., Lukashev D., Meriin A.B., Thoidis G., Frishman D., Shneider A.M. (2009). Importance of mRNA secondary structural elements for the expression of influenza virus genes. OMICS.

[10] Carlini D.B., Chen Y., Stephan W. (2001). The relationship between third-codon position nucleotide content, codon bias, mRNA secondary structure and gene expression in the drosophilid alcohol dehydrogenase genes Adh and Adhr. Genetics.

[11] Nackley A.G., Shabalina S.A., Tchivileva I.E., Satterfield K., Korchynskyi O., Makarov S.S., Maixner W., Diatchenko L. (2006). Human catechol-O-methyltransferase haplotypes modulate protein expression by altering mRNA secondary structure. Science.

[12] Gu W., Zhou T., Wilke C.O. (2010). A universal trend of reduced mRNA stability near the translation-initiation site in prokaryotes and eukaryotes. PLoS Comput. Biol..

[13] Del Campo C., Bartholomäus A., Fedyunin I., Ignatova Z. (2015). Secondary Structure across the Bacterial Transcriptome Reveals Versatile Roles in mRNA Regulation and Function. PLoS Genet..

[14] Shabalina S.A., Ogurtsov A.Y., Spiridonov N.A. (2006). A periodic pattern of mRNA secondary structure created by the genetic code. Nucleic Acids Res..

[15] Simmonds P., Tuplin A., Evans D.J. (2004). Detection of genome-scale ordered RNA structure (GORS) in genomes of positive-stranded RNA viruses: Implications for virus evolution and host persistence. RNA.

[16] Meyer I.M., Miklós I. (2005). Statistical evidence for conserved, local secondary structure in the coding regions of eukaryotic mRNAs and pre-mRNAs. Nucleic Acids Res..

[17] Olivier C., Poirier G., Gendron P., Boisgontier A., Major F., Chartrand P. (2005). Identification of a conserved RNA motif essential for She2p recognition and mRNA localization to the yeast bud. Mol. Cell. Biol..

[18] Fricke M., Gerst R., Ibrahim B., Niepmann M., Marz M. (2019). Global importance of RNA secondary structures in protein-coding sequences. Bioinformatics.

[19] Thurner C., Witwer C., Hofacker I.L., Stadler P.F. (2004). Conserved RNA secondary structures in Flaviviridae genomes. J. Gen. Virol..

[20] Fricke M., Dünnes N., Zayas M., Bartenschlager R., Niepmann M., Marz M. (2015). Conserved RNA secondary structures and long-range interactions in hepatitis C viruses. RNA.

[21] Pirakitikulr N., Kohlway A., Lindenbach B.D., Pyle A.M. (2016). The Coding Region of the HCV Genome Contains a Network of Regulatory RNA Structures. Mol. Cell.

[22] Simmonds P., Smith D.B. (1999). Structural constraints on RNA virus evolution. J. Virol..

[23] Moss W.N., Priore S.F., Turner D.H. (2011). Identification of potential conserved RNA secondary structure throughout influenza A coding regions. RNA.

[24] Clyde K., Harris E. (2006). RNA secondary structure in the coding region of dengue virus type 2 directs translation start codon selection and is required for viral replication. J. Virol..

[25] Goz E., Tuller T. (2015). Widespread signatures of local mRNA folding structure selection in four Dengue virus serotypes. BMC Genomics..

[26] Goz E., Tuller T. (2016). Evidence of a Direct Evolutionary Selection for Strong Folding and Mutational Robustness Within HIV Coding Regions. J. Comput. Biol..

[27] Díez J., Jungfleisch J. (2017). Translation control: Learning from viruses, again. RNA Biol..

[28] Pruitt K.D., Tatusova T., Brown G.R., Maglott D.R. (2012). NCBI Reference Sequences (RefSeq): Current status, new features and genome annotation policy. Nucleic Acids Res..

[29] Lorenz R., Bernhart S.H., Höner Zu Siederdissen C., Tafer H., Flamm C., Stadler P.F., Hofacker I.L. (2011). ViennaRNA Package 2.0. Algorithms Mol. Biol..

[30] Gruber A.R., Findeiß S., Washietl S., Hofacker I.L., Stadler P.F. (2010). RNAz 2.0: Improved noncoding RNA detection. Pac. Symp Biocomput.

[31] O’Leary N.A., Wright M.W., Brister J.R., Ciufo S., Haddad D., McVeigh R., Rajput B., Robbertse B., Smith-White B., Ako-Adjei D. (2016). Reference sequence (RefSeq) database at NCBI: Current status, taxonomic expansion, and functional annotation. Nucleic Acids Res..

[32] Hulo C., de Castro E., Masson P., Bougueleret L., Bairoch A., Xenarios I., Le Mercier P. (2011). ViralZone: A knowledge resource to understand virus diversity. Nucleic Acids Res..

[33] Sievers F., Higgins D.G. (2014). Clustal omega. Curr. Protoc. Bioinformatics.

[34] Eddy S.R. (2009). A new generation of homology search tools based on probabilistic inference. Genome Inform..

[35] Remmert M., Biegert A., Hauser A., Söding J. (2011). HHblits: Lightning-fast iterative protein sequence searching by HMM-HMM alignment. Nat. Methods.

[36] Enright A.J., Van Dongen S., Ouzounis C.A. (2002). An efficient algorithm for large-scale detection of protein families. Nucleic Acids Res..

[37] (2015). UniProt Consortium UniProt: A hub for protein information. Nucleic Acids Res..

[38] Altschul S.F., Gish W., Miller W., Myers E.W., Lipman D.J. (1990). Basic local alignment search tool. J. Mol. Biol..

[39] Mihara T., Nishimura Y., Shimizu Y., Nishiyama H., Yoshikawa G., Uehara H., Hingamp P., Goto S., Ogata H. (2016). Linking Virus Genomes with Host Taxonomy. Viruses.

[40] Kazlauskas D., Krupovic M., Venclovas Č. (2016). The logic of DNA replication in double-stranded DNA viruses: Insights from global analysis of viral genomes. Nucleic Acids Res..

[41] Saitou N., Nei M. (1987). The neighbor-joining method: A new method for reconstructing phylogenetic trees. Mol. Biol. Evol..

[42] Stajich J.E., Block D., Boulez K., Brenner S.E., Chervitz S.A., Dagdigian C., Fuellen G., Gilbert J.G.R., Korf I., Lapp H. (2002). The Bioperl toolkit: Perl modules for the life sciences. Genome Res..

[43] Will S., Joshi T., Hofacker I.L., Stadler P.F., Backofen R. (2012). LocARNA-P: Accurate boundary prediction and improved detection of structural RNAs. RNA.

[44] Cui X., Lu Z., Wang S., Jing-Yan Wang J., Gao X. (2016). CMsearch: Simultaneous exploration of protein sequence space and structure space improves not only protein homology detection but also protein structure prediction. Bioinformatics.

[45] Tuller T., Veksler-Lublinsky I., Gazit N., Kupiec M., Ruppin E., Ziv-Ukelson M. (2011). Composite effects of gene determinants on the translation speed and density of ribosomes. Genome Biol..

[46] Faure G., Ogurtsov A.Y., Shabalina S.A., Koonin E.V. (2016). Role of mRNA structure in the control of protein folding. Nucleic Acids Res..

[47] Ashburner M., Ball C.A., Blake J.A., Botstein D., Butler H., Cherry J.M., Davis A.P., Dolinski K., Dwight S.S., Eppig J.T. (2000). Gene ontology: Tool for the unification of biology. The Gene Ontology Consortium. Nat. Genet..

[48] Vandivier L., Li F., Zheng Q., Willmann M., Chen Y., Gregory B. (2013). Arabidopsis mRNA secondary structure correlates with protein function and domains. Plant. Signal. Behav..

[49] Binns D., Dimmer E., Huntley R., Barrell D., O’Donovan C., Apweiler R. (2009). QuickGO: A web-based tool for Gene Ontology searching. Bioinformatics.

[50] Giglio M., Tauber R., Nadendla S., Munro J., Olley D., Ball S., Mitraka E., Schriml L.M., Gaudet P., Hobbs E.T. (2019). ECO, the Evidence & Conclusion Ontology: Community standard for evidence information. Nucleic Acids Res..

[51] Supek F., Bošnjak M., Škunca N., Šmuc T. (2011). REVIGO summarizes and visualizes long lists of gene ontology terms. PLoS ONE.

[52] Jiang T., Nogales A., Baker S.F., Martinez-Sobrido L., Turner D.H. (2016). Mutations Designed by Ensemble Defect to Misfold Conserved RNA Structures of Influenza A Segments 7 and 8 Affect Splicing and Attenuate Viral Replication in Cell Culture. PLoS ONE.

[53] Kutchko K.M., Madden E.A., Morrison C., Plante K.S., Sanders W., Vincent H.A., Cruz Cisneros M.C., Long K.M., Moorman N.J., Heise M.T. (2018). Structural divergence creates new functional features in alphavirus genomes. Nucleic Acids Res..

[54] Weinberg Z., Breaker R.R. (2011). R2R--software to speed the depiction of aesthetic consensus RNA secondary structures. BMC Bioinformatics.

[55] Wang X., Li P., Gutenkunst R.N. (2017). Systematic Effects Of mRNA Secondary Structure On Gene Expression And Molecular Function In Budding Yeast. BioRxiv.

[56] Li F., Zheng Q., Vandivier L.E., Willmann M.R., Chen Y., Gregory B.D. (2012). Regulatory impact of RNA secondary structure across the Arabidopsis transcriptome. Plant. Cell.

[57] Zur H., Tuller T. (2012). Strong association between mRNA folding strength and protein abundance in *S. cerevisiae*. EMBO Rep..

[58] Federhen S. (2012). The NCBI Taxonomy database. Nucleic Acids Res..

[59] Yachdav G., Wilzbach S., Rauscher B., Sheridan R., Sillitoe I., Procter J., Lewis S.E., Rost B., Goldberg T. (2016). MSAViewer: Interactive JavaScript visualization of multiple sequence alignments. Bioinformatics.

[60] Waterhouse A.M., Procter J.B., Martin D.M.A., Clamp M., Barton G.J. (2009). Jalview Version 2--a multiple sequence alignment editor and analysis workbench. Bioinformatics.

