# Supplementary material for: Conserved Secondary Structures in Viral mRNAs
Source: Viruses. 2019 Apr 29;11(5):401. doi: 10.3390/v11050401 (PMC6563262; doi:10.3390/v11050401)
Supplement: Supplementary file 1 [file viruses-11-00401-s001.zip › supplement/Fig_S1.html]

Javascript must be enabled to view this page.

magnitude
magnitudeUnassigned

krona\_input

1
6251

6250

51

1

1

1

1

1

1

1

1

1

1

1

1

1

1

1

1

1

1

1

1

1

1

1

1

1

1

1

1

1

1

1

1

1

1

1

1

1

1

1

1

1

1

1

1

1

1

1

1

1

1

1

58

45

1

1

1

1

1

1

1

1

1

1

1

1

1

1

1

1

1

1

1

1

1

1

1

1

1

1

1

1

27

1

1

1

1

1

1

1

1

1

1

1

1

1

1

1

1

1

1

1

1

1

1

1

1

1

1

1

1

1

1

1

1

1

4

1

1

1

1

1

1

1

1

3

1

1

1

1

1

1

1

1

1

871

8

2

1

1

4

1

1

1

1

2

1

1

88

1

1

1

1

1

1

1

1

1

1

1

1

1

1

1

1

1

1

1

1

1

1

1

1

1

1

1

1

1

1

1

1

1

1

1

1

1

1

1

1

1

1

1

1

1

1

1

1

1

1

1

1

1

1

1

1

1

1

1

1

1

1

1

1

1

1

1

1

1

1

1

1

1

1

1

1

1

1

1

1

1

1

1

1

1

1

1

1

43

14

4

1

1

1

1

1

8

1

1

1

1

1

1

1

1

1

1

1

1

1

8

1

1

1

3

2

1

1

1

1

4

1

1

1

1

1

21

1

1

1

1

1

1

1

1

1

1

1

1

1

1

1

1

1

1

1

1

1

1

53

2

1

1

1

1

1

1

1

1

22

1

1

1

1

1

1

1

1

1

1

1

1

1

1

1

1

1

5

1

1

1

1

1

9

1

1

1

1

1

1

1

1

1

1

1

2

1

1

12

1

1

1

3

1

1

1

1

1

1

1

1

1

1

1

1

1

410

3

1

1

1

2

1

1

1

2

1

1

355

1

1

1

1

1

1

1

1

1

1

1

1

1

1

1

1

1

1

1

1

1

1

1

1

1

1

1

1

1

1

1

1

1

1

1

1

1

1

1

1

1

1

1

1

1

1

1

1

1

1

1

1

1

1

1

1

1

1

1

1

1

1

1

1

1

1

1

1

1

1

1

1

1

1

1

1

1

1

1

1

1

1

1

1

1

1

1

1

1

1

1

1

1

1

1

1

1

1

1

1

1

1

1

1

1

1

1

1

1

1

1

1

1

1

1

1

1

1

1

1

1

1

1

1

1

1

1

1

1

1

1

1

1

1

1

1

1

1

1

1

1

1

1

1

1

1

1

1

1

1

1

1

1

1

1

1

1

1

1

1

1

1

1

1

1

1

1

110

1

1

1

1

1

1

1

1

1

1

1

1

1

1

1

1

1

1

1

1

1

1

1

1

1

1

1

1

1

1

1

1

1

1

1

1

1

1

1

1

1

1

1

1

1

1

1

1

1

1

1

1

1

1

1

1

1

1

1

1

1

1

1

1

1

1

1

1

1

1

1

1

1

1

1

1

1

1

1

1

1

1

1

1

1

1

1

1

1

1

1

1

1

1

1

1

1

1

1

1

1

1

1

1

1

1

1

1

1

1

1

1

1

1

1

1

1

1

1

1

1

1

1

1

1

1

1

1

1

1

1

1

1

1

1

1

1

1

1

1

1

1

1

1

1

1

1

1

1

1

1

1

1

1

1

1

1

1

1

1

1

1

1

1

1

1

1

1

1

1

1

1

1

1

1

1

1

1

1

1

1

1

1

1

1

1

1

1

11

1

2

1

1

1

1

1

1

1

1

1

1

1

1

1

1

35

1

1

1

1

1

1

1

1

1

1

1

1

1

1

1

1

1

1

1

1

1

1

1

1

1

1

6

1

1

1

1

1

1

1

1

1

78

50

2

1

1

1

1

1

4

1

1

1

1

2

1

1

1

8

1

1

1

3

1

1

1

1

1

10

1

7

1

1

1

1

1

1

1

1

1

2

1

1

18

1

1

1

1

1

1

1

1

10

1

1

1

1

1

1

1

1

1

1

3

1

1

1

4

1

1

1

1

24

1

1

3

1

1

1

1

1

1

11

1

1

1

1

1

2

1

1

1

1

1

1

1

6

1

1

1

1

1

1

1

1

1

39

1

1

8

1

1

1

4

1

1

1

1

1

2

1

1

3

1

1

1

2

1

1

1

8

1

1

1

1

1

1

1

1

1

1

2

1

1

3

1

1

1

9

1

1

1

1

1

1

1

1

1

152

28

1

1

1

1

1

1

1

1

12

1

1

1

1

1

1

1

1

1

1

1

1

1

1

1

1

1

1

1

1

18

1

1

1

1

1

1

1

1

1

1

1

1

1

1

1

1

1

1

98

1

1

1

1

1

1

1

1

1

1

1

17

1

1

1

1

1

1

1

1

1

1

1

1

1

1

1

1

1

1

1

1

1

1

1

1

1

1

1

1

1

1

1

1

1

1

1

1

1

1

1

1

1

1

1

1

1

1

1

1

1

1

1

1

1

1

1

1

1

1

1

1

1

1

1

1

1

1

1

1

1

1

1

1

1

1

1

1

1

1

1

1

1

1

1

1

1

1

1

8

1

7

1

1

1

1

1

1

1

129

1

1

1

1

1

1

1

1

1

1

1

1

1

1

1

1

1

1

1

16

1

1

1

1

1

1

1

1

1

1

1

1

1

1

1

1

1

1

1

1

1

1

1

1

1

1

1

1

1

1

1

1

1

1

1

1

1

1

1

1

1

1

1

1

1

1

1

1

1

1

1

1

1

1

1

1

1

1

1

1

1

1

1

1

1

1

1

1

1

1

1

1

1

1

1

1

1

1

1

1

1

1

1

1

1

1

1

1

1

1

1

1

1

1

1

1

1

1

1

1

1

1

1

1

1

1

1

1

1

1

2640

21

3

1

2

1

1

1

1

7

1

6

1

1

1

1

1

1

1

1

6

1

1

2

1

1

1

1

3

1

1

1

1

1

1

5

5

1

1

2

1

1

1

141

1

1

1

1

1

1

1

1

1

1

1

1

1

1

1

1

4

1

1

1

1

1

1

1

1

1

1

1

1

1

1

1

3

1

1

1

1

1

7

1

1

1

1

1

1

1

1

2

1

1

1

1

3

1

1

1

1

1

14

1

1

1

1

1

1

1

1

1

1

1

1

1

1

1

1

1

1

6

1

1

1

1

1

1

1

34

1

1

27

1

1

1

1

1

1

1

1

1

1

1

1

1

1

1

1

1

1

1

1

1

1

1

1

1

1

1

5

1

1

1

1

1

1

1

3

1

1

1

1

2

1

1

1

1

2

1

1

1

1

1

1

1

1

7

1

1

1

1

1

1

1

1

1

1

24

1

1

1

1

1

1

1

1

1

1

1

1

2

1

1

1

1

1

1

1

1

1

1

1

1

2

1

1

1

1

1

1

1

1

1

1

1

1

1

1

6

2

1

1

3

1

1

1

1

1

15

8

6

1

1

1

1

1

1

1

1

1

1

7

6

3

1

1

1

1

1

1

1

1

2

2

1

1

1951

341

1

1

124

1

1

1

1

1

1

1

1

1

1

1

1

1

1

1

1

1

1

1

1

1

1

1

1

1

1

1

1

1

1

1

1

1

1

1

1

1

1

1

1

1

1

1

1

1

1

1

1

1

1

1

1

1

1

1

1

1

1

1

1

1

1

1

1

1

1

1

1

1

1

15

1

1

1

1

1

1

1

1

1

1

1

1

1

1

1

1

1

1

1

1

1

1

1

1

1

1

1

1

1

1

1

1

1

1

1

1

1

1

1

1

1

1

1

1

1

1

1

1

1

1

1

1

1

1

2

1

1

19

18

1

1

1

1

1

1

1

1

1

1

1

1

1

1

1

1

1

1

1

4

1

1

1

1

7

1

1

1

1

1

1

1

2

1

1

1

8

1

1

1

1

1

1

1

1

2

1

1

2

1

1

4

1

1

1

1

1

6

2

1

1

4

1

1

1

1

18

4

1

1

1

1

3

1

1

1

7

5

1

1

1

1

1

1

1

3

1

1

1

1

1

2

1

1

11

7

1

1

1

1

1

1

1

1

1

1

1

109

12

1

1

1

1

1

1

1

1

1

1

1

1

39

1

1

36

1

1

1

1

1

1

1

1

1

1

1

1

1

1

1

1

1

1

1

1

1

1

1

1

1

1

1

1

1

1

1

1

1

1

1

1

1

1

1

21

1

17

1

1

1

1

1

1

1

1

1

1

1

1

1

1

1

1

1

1

1

1

10

1

1

6

1

1

1

1

1

1

1

1

9

1

1

1

1

1

1

1

1

1

4

1

1

1

1

6

1

1

1

1

1

1

7

1

1

1

1

1

1

1

2

1

1

2

1

1

3

1

1

1

1

1

1

3

1

1

1

1

1

4

1

1

2

1

1

1

1

1

1

3

1

1

1

19

1

1

1

1

1

1

1

1

1

1

1

1

1

1

1

1

1

1

1

526

28

4

2

1

1

1

1

1

1

1

2

1

1

1

3

1

1

1

2

1

1

2

1

1

10

1

1

1

1

1

1

1

1

1

1

4

1

3

1

1

1

5

1

1

1

1

1

2

1

1

1

3

1

1

1

3

1

1

1

1

1

6

1

1

1

1

1

1

2

1

1

13

11

1

1

1

1

1

1

1

1

1

1

1

1

2

1

1

222

1

1

1

1

1

1

1

1

1

1

1

1

1

1

1

1

1

1

1

1

1

1

1

1

1

1

1

1

1

1

1

1

1

1

1

1

1

1

1

1

1

1

1

1

1

1

1

1

1

1

1

1

1

1

1

1

1

1

1

1

1

1

1

1

1

1

1

1

1

1

1

1

1

1

1

1

1

1

1

1

1

1

1

1

1

1

1

1

1

1

1

1

1

1

1

1

1

1

1

1

1

1

1

1

1

1

1

1

1

1

1

1

1

1

1

1

1

1

1

1

1

1

1

1

1

1

1

1

1

1

1

1

1

1

1

1

1

1

1

1

1

1

1

1

1

1

1

1

1

1

1

1

1

1

1

1

1

1

1

1

1

1

1

1

1

1

1

1

1

1

1

1

1

1

1

1

1

1

1

1

1

1

1

1

1

1

1

1

1

1

1

1

1

1

1

1

1

1

1

1

1

1

1

1

1

1

1

1

1

1

1

1

1

1

1

1

1

1

1

1

1

1

2

1

1

2

1

1

1

1

1

1

13

1

1

1

1

1

1

1

1

1

1

1

1

1

1

1

1

1

1

1

1

5

1

1

1

1

1

1

1

2

1

1

3

1

1

1

1

1

3

1

1

1

1

1

96

3

1

1

1

5

1

1

1

1

1

2

1

1

2

1

1

4

1

1

1

1

67

1

1

1

1

57

1

1

1

1

1

1

1

1

1

1

1

1

1

1

1

1

1

1

1

1

1

1

1

1

1

1

1

1

1

1

1

1

1

1

1

1

1

1

1

1

1

1

1

1

1

1

1

1

1

1

1

1

1

1

1

1

1

1

1

1

1

1

1

2

1

1

4

1

1

1

1

1

1

2

1

1

4

1

1

1

1

1

1

1

7

1

1

1

1

1

1

1

2

1

1

1

1

2

1

1

1

1

2

1

1

29

24

1

1

1

1

1

1

1

14

1

1

1

1

1

1

1

1

1

1

1

1

1

1

1

1

1

5

1

1

1

1

1

4

1

1

1

1

2

1

1

15

15

1

1

1

1

1

1

1

1

1

1

1

1

1

1

1

2

1

1

4

1

1

1

1

1

3

1

1

1

2

1

1

1

1

5

1

1

1

1

1

6

4

1

1

1

1

1

2

1

1

13

4

1

1

1

1

5

1

1

1

1

1

4

1

1

1

1

5

1

3

1

1

1

1

3

2

1

1

1

1065

3

1

1

1

3

1

1

1

2

1

1

5

1

1

1

1

1

1

1

2

1

1

1

1

1

1

1

33

1

1

1

1

1

1

1

1

1

1

1

1

1

1

1

1

1

1

1

1

1

1

1

1

1

1

1

1

3

1

1

1

1

1

1

1

2

1

1

2

1

1

6

4

1

1

1

1

1

1

1

1

1

1

1

1

6

1

1

1

1

1

1

1

1

1

7

1

1

1

1

1

1

1

1

1

46

1

1

1

1

1

1

1

1

1

1

1

1

1

1

1

1

1

1

1

1

1

1

1

1

1

1

1

1

1

1

1

1

1

1

1

1

1

1

1

1

1

1

1

1

1

1

2

1

1

1

1

2

1

1

5

1

1

1

1

1

2

1

1

5

1

1

1

1

1

5

1

1

1

1

1

5

1

1

1

1

1

6

1

1

1

1

1

1

1

5

1

1

1

1

1

10

1

1

1

1

1

1

1

1

1

1

2

1

1

7

1

1

1

1

1

1

1

2

1

1

1

1

1

1

3

1

1

1

1

2

1

1

1

1

1

1

1

1

1

1

3

1

1

1

33

1

1

1

1

1

1

1

1

1

1

1

1

1

1

1

1

1

1

1

1

1

1

1

1

4

1

1

1

1

1

1

1

1

1

1

1

2

1

1

4

1

1

1

1

3

1

1

1

6

1

1

1

1

1

1

1

1

3

1

1

1

2

1

1

1

1

2

1

1

1

1

20

5

1

1

1

1

1

1

1

2

1

1

5

1

1

1

1

1

7

1

1

1

1

1

1

1

3

1

1

1

4

1

1

1

1

2

1

1

2

1

1

3

1

1

1

5

1

1

1

1

1

2

1

1

1

1

1

1

32

1

1

8

1

1

1

1

1

1

1

1

1

1

1

4

1

1

1

1

18

1

1

4

1

1

1

1

1

6

1

1

1

1

1

1

1

3

1

1

1

1

1

1

11

1

1

1

1

1

1

1

3

1

1

1

1

1

1

3

1

1

1

2

1

1

1

1

611

1

1

1

1

1

1

1

1

1

1

1

1

1

1

1

1

1

1

1

1

1

1

1

1

1

1

1

1

1

1

1

1

1

1

1

1

1

1

1

1

1

1

1

1

1

1

1

1

1

1

1

1

1

1

1

1

1

1

1

1

1

1

1

1

1

1

1

1

1

1

1

1

1

1

1

1

1

1

1

1

1

1

1

1

1

1

1

1

1

1

1

1

1

1

1

1

1

1

1

1

1

1

1

1

1

1

1

1

1

1

1

1

1

1

1

1

1

1

1

1

1

1

1

1

1

1

1

1

1

1

1

1

1

1

1

1

1

1

1

1

1

1

1

1

1

1

1

1

1

1

1

1

1

1

1

1

1

1

1

1

1

1

1

1

1

1

1

1

1

1

1

1

1

1

1

1

1

1

1

1

1

1

1

1

1

1

1

1

1

1

1

1

1

1

1

1

1

1

1

1

1

1

1

1

1

1

1

1

1

1

1

1

1

1

1

1

1

1

1

1

1

1

1

1

1

1

1

1

1

1

1

1

1

1

1

1

1

1

1

1

1

1

1

1

1

1

1

1

1

1

1

1

1

1

1

1

1

1

1

1

1

1

1

1

1

1

1

1

1

1

1

1

1

1

1

1

1

1

1

1

1

1

1

1

1

1

1

1

1

1

1

1

1

1

1

1

1

1

1

1

1

1

1

1

1

1

1

1

1

1

1

1

1

1

1

1

1

1

1

1

1

1

1

1

1

1

1

1

1

1

1

1

1

1

1

1

1

1

1

1

1

1

1

1

1

1

1

1

1

1

1

1

1

1

1

1

1

1

1

1

1

1

1

1

1

1

1

1

1

1

1

1

1

1

1

1

1

1

1

1

1

1

1

1

1

1

1

1

1

1

1

1

1

1

1

1

1

1

1

1

1

1

1

1

1

1

1

1

1

1

1

1

1

1

1

1

1

1

1

1

1

1

1

1

1

1

1

1

1

1

1

1

1

1

1

1

1

1

1

1

1

1

1

1

1

1

1

1

1

1

1

1

1

1

1

1

1

1

1

1

1

1

1

1

1

1

1

1

1

1

1

1

1

1

1

1

1

1

1

1

1

1

1

1

1

1

1

1

1

1

1

1

1

1

1

1

1

1

1

1

1

1

1

1

1

1

1

1

1

1

1

1

1

1

1

1

1

1

1

1

1

1

1

1

1

1

1

1

1

1

1

1

1

1

1

1

1

1

1

1

1

1

1

2

1

1

1

1

1

1

1

1

1

1

1

1

1

1

1

1

1

1

1

1

1

1

1

1

1

1

1

1

1

1

1

1

1

1

1

1

1

1

1

1

1

1

1

1

1

1

1

1

1

1

1

1

1

1

1

1

1

1

1

1

1

1

1

1

1

1

1

1

17

1

1

1

1

1

1

1

1

1

1

1

1

1

1

1

1

1

2

1

1

6

1

1

1

1

1

1

2

1

1

1

1

1

1

7

5

1

1

1

1

1

1

1

2

1

1

3

1

1

1

3

1

1

1

7

1

1

1

1

1

1

1

4

1

1

1

1

33

1

30

1

1

1

1

1

1

1

1

1

1

1

1

1

1

1

1

1

1

1

1

1

1

1

1

1

1

1

1

1

1

1

1

3

1

1

1

4

1

1

1

1

9

6

1

1

1

1

1

1

1

1

2

1

1

10

2

1

1

1

1

7

1

1

2

1

1

1

1

1

1

1

1

5

1

1

1

1

3

1

1

1

2

2

1

1

73

45

1

1

1

1

1

1

1

1

1

1

1

1

1

1

1

1

1

1

1

1

1

1

1

1

1

1

1

1

1

14

1

1

1

1

1

1

1

1

1

1

1

1

1

1

1

1

3

1

1

1

1

1

1

22

1

9

1

1

1

1

1

1

1

1

1

1

1

1

1

1

1

1

1

1

1

1

1

2

1

1

1

1

1

5

2

1

1

3

1

1

1

3

3

1

2

1

1

40

1

1

1

1

1

1

1

1

1

1

1

1

1

1

1

1

1

1

1

1

1

1

1

1

1

1

1

1

1

1

1

1

1

1

1

1

1

1

1

1

65

4

1

1

1

1

42

1

1

1

1

1

1

1

1

1

1

1

1

1

1

1

1

1

1

1

1

1

1

1

1

1

4

1

1

1

1

1

7

1

1

1

1

1

1

1

1

1

1

1

1

1

1

1

1

1

1

1

1

7

1

1

3

1

1

1

1

1

12

1

1

1

1

1

1

1

1

1

1

1

1

1

7

4

1

1

1

1

2

1

1

1

1

1

1

1

1

1

1

37

1

1

1

1

1

1

1

1

1

1

1

1

1

1

1

1

16

1

15

1

1

1

1

1

1

1

1

1

1

1

1

1

1

1

1

1

1

1

1

1

43

34

1

1

1

1

2

1

1

2

1

1

1

1

1

1

2

1

1

5

1

1

1

1

1

1

1

1

1

3

1

1

1

4

1

1

1

1

1

11

1

1

1

1

1

1

1

1

1

1

1

1

7

5

1

3

1

1

1

1

1

1

1

1

2

1

1

4

1

1

3

1

1

1

4

1

1

3

1

1

1

1

1

1

1

80

7

2

1

1

1

1

4

1

1

1

1

1

1

1

1

1

71

32

6

1

1

2

1

1

1

1

1

1

2

1

1

1

2

1

1

9

1

1

1

1

1

1

1

1

1

1

12

1

1

1

1

1

1

1

1

1

1

1

1

18

4

1

1

1

1

7

1

1

1

1

1

1

1

2

1

1

3

1

1

1

2

1

1

21

5

2

1

1

1

1

1

3

2

1

1

1

7

1

1

1

1

1

1

1

1

3

1

1

1

3

1

1

1

90

2

1

1

5

1

1

1

1

1

1

1

12

1

1

1

1

1

1

1

1

1

1

1

1

58

1

1

1

1

1

1

1

1

1

1

1

1

1

1

1

1

1

1

1

1

1

1

1

1

1

1

1

1

1

1

1

1

1

1

1

1

1

1

1

1

1

1

1

1

1

1

1

1

1

1

1

1

1

1

1

1

1

1

11

1

1

1

1

1

1

1

1

1

1

1

1

1

25

6

2

1

1

1

1

1

1

1

1

1

7

1

6

1

1

1

1

1

1

1

1

2

1

1

8

1

1

1

1

1

1

1

1

184

2

1

1

1

1

3

3

1

1

1

179

1

1

3

1

1

1

165

66

12

1

1

1

1

1

1

1

4

1

1

1

1

1

54

1

1

1

1

1

1

1

1

1

1

1

1

37

1

1

1

1

1

1

1

1

1

1

1

1

1

1

1

1

1

1

1

1

1

1

1

1

1

1

1

1

1

1

1

1

1

1

1

1

1

1

1

1

1

1

2

1

1

97

1

1

1

69

1

1

1

1

1

1

1

1

1

1

1

1

1

1

1

1

1

1

1

1

1

1

1

1

1

1

1

1

1

1

1

1

1

1

1

1

1

1

1

1

1

1

1

1

1

1

1

1

1

1

1

1

1

1

1

1

1

1

1

1

1

1

1

1

1

1

1

1

1

1

1

1

1

1

1

1

1

1

1

1

1

1

1

1

1

1

1

1

1

1

1

1

1

1

10

1

1

3

1

1

1

5

1

1

1

1

1

1

1

224

1

1

1

24

1

1

1

1

1

1

1

1

1

1

1

1

1

1

1

1

1

1

1

1

1

1

1

1

62

24

1

1

1

1

1

1

1

1

1

1

1

1

1

1

1

1

1

1

1

1

1

1

1

1

24

1

1

1

1

1

1

1

1

1

1

13

1

1

1

1

1

1

1

1

1

1

1

1

1

1

3

1

1

1

1

7

1

1

1

1

3

1

1

1

1

1

3

1

1

1

4

1

1

3

1

1

1

5

1

1

4

1

1

1

1

1

1

1

49

5

1

1

1

1

1

1

13

1

1

1

1

1

1

1

1

2

1

1

1

1

1

23

1

1

1

1

1

1

1

1

1

1

1

1

1

1

1

1

1

1

1

1

1

1

1

6

1

1

1

1

1

1

2

1

1

8

8

1

1

1

5

1

1

1

1

1

70

33

3

1

1

1

3

1

1

1

1

1

1

8

1

1

1

1

1

1

1

1

1

18

1

1

1

1

1

1

1

1

4

1

1

1

1

1

1

1

1

1

1

31

6

1

1

1

1

1

1

1

7

1

1

3

1

1

1

1

1

2

1

1

3

1

1

1

1

1

1

6

2

1

1

1

1

1

2

1

1

2

1

1

1

4

1

1

1

1

1

6

1

1

1

1

1

1

45

10

10

1

1

1

2

1

1

1

1

4

1

1

3

3

1

1

1

2

1

1

16

3

1

1

1

1

1

5

1

1

1

1

1

7

1

1

1

1

1

1

1

1

19

11

3

1

1

1

1

1

1

1

1

1

1

1

5

1

1

1

1

1

1

1

1

1

1

1

1

1

1

4

1

1

1

1

1036

732

45

6

5

1

1

1

1

1

1

4

1

1

1

1

4

1

1

1

1

2

2

1

1

29

1

1

1

1

12

1

1

1

1

1

1

1

1

1

1

1

1

1

1

4

1

1

1

1

2

1

1

1

1

1

5

1

1

1

1

1

40

2

1

1

1

28

1

1

11

1

1

1

1

1

1

1

1

1

1

1

1

1

1

1

1

1

1

1

1

1

1

1

1

1

1

2

1

1

8

1

2

1

1

1

1

1

1

1

1

1

15

5

1

1

1

1

1

5

1

1

1

1

1

5

1

1

1

1

1

1

27

1

1

1

1

1

1

1

1

1

1

5

1

1

1

1

1

1

1

1

1

1

1

1

1

1

1

1

1

1

1

1

1

1

1

37

2

1

1

29

1

1

25

1

1

1

1

1

1

1

1

1

1

1

1

1

1

1

1

1

1

1

1

1

1

1

1

1

1

1

6

4

1

1

1

1

1

1

1

1

1

1

1

15

1

1

14

4

1

1

1

1

1

1

1

1

1

4

1

1

1

1

1

1

1

12

6

4

1

1

1

1

1

1

3

1

1

1

3

1

1

1

9

7

7

4

1

1

1

1

1

1

2

1

1

2

1

1

1

1

5

3

1

1

1

1

1

1

1

1

96

1

1

1

63

1

1

2

1

1

2

1

1

4

1

3

1

1

1

1

1

1

1

2

1

1

1

1

1

13

1

1

1

1

1

1

1

1

1

2

1

1

1

1

1

1

1

1

1

3

1

1

1

13

1

1

1

1

1

1

1

1

1

1

1

1

1

1

1

1

1

1

1

1

5

3

1

1

1

1

1

1

1

1

1

1

3

1

1

1

1

1

1

1

1

1

2

1

1

1

22

1

1

3

1

1

1

1

4

1

1

1

1

10

2

1

1

3

1

1

1

5

1

1

1

1

1

1

1

2

1

1

1

1

6

1

1

1

1

1

1

2

1

1

1

1

1

1

1

1

1

1

1

1

144

2

1

1

2

1

1

84

5

1

1

1

1

1

24

4

2

1

1

1

1

2

1

1

3

1

1

1

1

1

2

1

1

6

1

1

1

1

1

1

1

1

5

1

1

1

1

1

55

9

1

1

1

3

1

1

1

1

1

1

44

1

1

1

1

1

1

1

1

1

1

1

1

1

1

1

1

1

1

1

1

1

1

1

1

1

1

1

1

10

1

1

1

1

1

1

1

1

1

1

1

1

1

1

1

1

2

1

1

1

1

1

5

1

1

2

1

1

1

1

1

1

1

50

1

1

7

1

1

1

1

1

1

1

2

1

1

2

1

1

1

1

35

1

1

1

1

1

1

1

1

1

1

1

1

1

1

1

1

1

1

1

1

1

1

1

4

1

1

1

1

1

1

1

1

1

1

1

1

1

1

1

1

4

1

1

1

1

1

1

1

35

1

1

1

1

20

2

1

1

3

1

1

1

2

1

1

1

6

1

1

1

1

1

1

1

1

2

1

1

1

1

1

3

2

1

1

1

4

1

1

1

1

6

1

1

1

1

1

1

1

1

1

16

6

1

1

2

1

1

1

1

9

1

1

1

1

1

1

1

1

1

1

1

1

102

1

1

1

3

1

1

1

3

1

1

1

1

1

4

1

1

1

1

3

1

1

1

83

1

1

1

1

1

1

1

1

1

1

1

1

1

1

1

1

1

1

1

1

1

1

1

1

1

1

1

1

1

13

1

1

1

1

1

1

1

1

1

1

1

1

1

1

1

1

1

1

1

1

1

1

1

1

1

1

1

1

1

1

1

1

1

1

1

1

1

1

1

1

1

1

1

1

1

1

1

1

1

1

1

1

1

1

4

1

1

1

1

66

3

1

1

1

1

1

3

1

1

1

1

1

4

1

1

1

1

1

1

7

1

1

1

1

1

1

1

11

1

1

1

1

1

1

1

1

1

1

1

8

3

1

1

1

1

1

1

1

1

4

1

1

1

1

1

1

1

1

1

1

1

5

1

1

1

1

1

5

1

1

1

1

1

1

3

1

1

1

4

1

1

1

1

3

1

1

1

51

5

1

1

1

1

1

1

2

1

1

1

1

33

1

1

1

1

1

1

1

1

1

1

1

1

1

1

1

1

1

1

1

1

1

1

4

1

1

1

1

1

1

1

1

1

1

1

5

1

1

1

1

1

1

1

1

1

3

1

1

1

4

1

1

1

1

1

1

1

1

6

5

1

1

1

1

1

1

298

3

3

1

1

1

8

1

1

1

1

1

1

1

2

1

1

1

1

1

1

1

1

1

206

4

1

1

3

1

1

1

5

1

1

1

1

1

1

1

1

56

1

1

14

1

1

1

2

1

1

1

1

1

1

1

1

1

1

1

7

1

1

1

1

1

1

1

5

1

1

1

1

1

7

1

1

1

1

1

1

1

16

1

1

1

1

1

1

1

1

1

1

1

1

1

1

1

1

5

1

1

1

1

1

1

1

1

1

1

119

17

3

1

1

1

1

1

1

1

1

1

1

1

1

1

1

1

1

1

9

1

1

1

1

4

1

1

1

1

1

1

1

2

1

1

5

1

1

1

1

1

1

1

1

1

1

4

1

1

1

1

1

1

1

1

9

1

1

1

1

1

1

1

1

1

1

1

9

1

1

1

1

1

1

1

1

1

1

2

1

1

2

1

1

3

1

1

1

1

1

2

1

1

35

1

1

1

1

1

1

1

1

1

1

1

1

1

1

1

1

1

1

1

1

1

1

1

1

1

1

1

1

1

1

1

1

1

1

1

1

1

2

1

1

12

1

1

1

1

1

1

1

1

1

1

1

1

7

3

1

1

1

2

2

1

1

2

1

1

6

6

1

1

1

1

1

1

1

1

1

7

1

1

5

1

1

1

1

1

1

1

21

1

1

1

1

1

1

1

1

1

1

1

1

1

1

1

1

1

1

1

1

1

36

3

1

1

1

2

1

1

1

1

1

31

1

1

1

1

1

1

1

1

1

1

1

1

1

1

1

1

1

1

1

1

1

1

1

1

1

1

1

1

1

1

1

24

12

11

1

1

1

1

1

1

1

1

1

1

1

1

1

1

1

1

5

5

1

1

1

1

1

1

1

1

5

2

1

1

2

1

1

1

1

3

1

1

1

1

1

965

1

1

962

1

1

1

1

1

1

1

1

1

1

1

1

1

1

1

1

1

1

1

1

1

1

1

1

1

1

1

1

1

1

1

1

1

1

1

1

1

1

1

1

1

1

1

1

1

1

1

1

1

1

1

1

1

1

1

1

1

1

1

1

1

1

1

1

1

1

1

1

1

1

1

1

1

1

1

1

1

1

1

1

1

1

1

1

1

1

1

1

1

1

1

1

1

1

1

1

1

1

1

1

1

1

1

1

1

1

1

1

1

1

1

1

1

1

1

1

1

1

1

1

1

1

1

1

1

1

1

1

1

1

1

1

1

1

1

1

1

1

1

1

1

1

1

1

1

1

1

1

1

1

1

1

1

1

1

1

1

1

1

1

1

1

1

1

1

1

1

1

1

1

1

1

1

1

1

1

1

1

1

1

1

1

1

1

1

1

1

1

1

1

1

1

1

1

1

1

1

1

1

1

1

1

1

1

1

1

1

1

1

1

1

1

1

1

1

1

1

1

1

1

1

1

1

1

1

1

1

1

1

1

1

1

1

1

1

1

1

1

1

1

1

1

1

1

1

1

1

1

1

1

1

1

1

1

1

1

1

1

1

1

1

1

1

1

1

1

1

1

1

1

1

1

1

1

1

1

1

1

1

1

1

1

1

1

1

1

1

1

1

1

1

1

1

1

1

1

1

1

1

1

1

1

1

1

1

1

1

1

1

1

1

1

1

1

1

1

1

1

1

1

1

1

1

1

1

1

1

1

1

1

1

1

1

1

1

1

1

1

1

1

1

1

1

1

1

1

1

1

1

1

1

1

1

1

1

1

1

1

1

1

1

1

1

1

1

1

1

1

1

1

1

1

1

1

1

1

1

1

1

1

1

1

1

1

1

1

1

1

1

1

1

1

1

1

1

1

1

1

1

1

1

1

1

1

1

1

1

1

1

1

1

1

1

1

1

1

1

1

1

1

1

1

1

1

1

1

1

1

1

1

1

1

1

1

1

1

1

1

1

1

1

1

1

1

1

1

1

1

1

1

1

1

1

1

1

1

1

1

1

1

1

1

1

1

1

1

1

1

1

1

1

1

1

1

1

1

1

1

1

1

1

1

1

1

1

1

1

1

1

1

1

1

1

1

1

1

1

1

1

1

1

1

1

1

1

1

1

1

1

1

1

1

1

1

1

1

1

1

1

1

1

1

1

1

1

1

1

1

1

1

1

1

1

1

1

1

1

1

1

1

1

1

1

1

1

1

1

1

1

1

1

1

1

1

1

1

1

1

1

1

1

1

1

1

1

1

1

1

1

1

1

1

1

1

1

1

1

1

1

1

1

1

1

1

1

1

1

1

1

1

1

1

1

1

1

1

1

1

1

1

1

1

1

1

1

1

1

1

1

1

1

1

1

1

1

1

1

1

1

1

1

1

1

1

1

1

1

1

1

1

1

1

1

1

1

1

1

1

1

1

1

1

1

1

1

1

1

1

1

1

1

1

1

1

1

1

1

1

1

1

1

1

1

1

1

1

1

1

1

1

1

1

1

1

1

1

1

1

1

1

1

1

1

1

1

1

1

1

1

1

1

1

1

1

1

1

1

1

1

1

1

1

1

1

1

1

1

1

1

1

1

1

1

1

1

1

1

1

1

1

1

1

1

1

1

1

1

1

1

1

1

1

1

1

1

1

1

1

1

1

1

1

1

1

1

1

1

1

1

1

1

1

1

1

1

1

1

1

1

1

1

1

1

1

1

1

1

1

1

1

1

1

1

1

1

1

1

1

1

1

1

1

1

1

1

1

1

1

1

1

1

1

1

1

1

1

1

1

1

1

1

1

1

1

1

1

1

1

1

1

1

1

1

1

1

1

1

1

1

1

1

1

1

1

1

1

1

1

1

1

1

1

1

1

1

1

1

1

1

1

1

1

1

1

1

1

1

1

1

1

1

1

1

1

1

1

1

1

1

1

1

1

1

1

1

1

1

1

1

1

1

1

1

1

1

1

1

1

1

1

1

1

1

1

1

1

1

1

1

1

1

1

1

1

1

1

1

1

1

1

1

1

1

1

1

1

1

1

1

1

1

1

1

1

1

1

1

1

1

1

1

1

1

1

1

1

1

1

1

1

1

1

1

1

1

1

1

1

1

1

1

1

1

1

1

1

1

1

1

1

1

1

1

1

1

1

1

1

1

1

1

1

1

15

1

1

1

8

6

1

1

1

1

1

1

1

1

1

2

1

1

1

1

1

1

3

3

1

2

1

1

1

1

22

8

1

1

1

5

1

1

1

1

1

1

1

1

1

1

2

1

1

1

1

2

1

1

1

1

1

1

1

1

3

2

1

1

1

1

1

1

6

3

1

1

1

2

1

1

1

1
