# Supplementary material for: Conserved Secondary Structures in Viral mRNAs
Source: Viruses. 2019 Apr 29;11(5):401. doi: 10.3390/v11050401 (PMC6563262; doi:10.3390/v11050401)
Supplement: Supplementary file 1 [file viruses-11-00401-s001.zip › supplement/Table_S1_S2.pdf]

Table S1: Structural coverage of subVOG mRNAs associated with GO terms. The GO terms are clustered into functionally similar groups using Revigo. Only manually and experimentally annotated GO terms are shown. A color coded version of the table is provided online at http://subvogs.bio.wzw.tum.de/sup/table_s1.html.

GO term	Ontology	Description	Revigo cluster	Mean structural coverage	Number of subVOGs	Number of proteins
GO:0006139	biological process	nucleobase-containing compound metabolic process	1	0.1410	1	2
GO:0006259	biological process	DNA metabolic process	2	0.1410	1	2
GO:0006278	biological process	RNA-dependent DNA biosynthetic process	3	0.1410	1	2
GO:0006725	biological process	cellular aromatic compound metabolic process	4	0.1410	1	2
GO:0006807	biological process	nitrogen compound metabolic process	5	0.1410	1	2
GO:0008150	biological process	biological process	6	0.0689	10	59
GO:0008152	biological process	metabolic process	7	0.1410	1	2
GO:0009058	biological process	biosynthetic process	8	0.1410	1	2
GO:0009059	biological process	macromolecule biosynthetic process	9	0.1410	1	2
GO:0009894	biological process	regulation of catabolic process	10	0.1130	4	12
GO:0009987	biological process	cellular process	11	0.1410	1	2
GO:0010506	biological process	regulation of autophagy	12	0.1130	4	12
GO:0010508	biological process	positive regulation of autophagy	12	0.1130	4	12
GO:0010605	biological process	negative regulation of macromolecule metabolic process	13	0.1130	4	12
GO:0009892	biological process	negative regulation of metabolic process	13	0.1130	4	12
GO:0010629	biological process	negative regulation of gene expression	13	0.1130	4	12
GO:0010608	biological process	posttranscriptional regulation of gene expression	14	0.1130	4	12
GO:0018130	biological process	heterocycle biosynthetic process	15	0.1410	1	2
GO:0019222	biological process	regulation of metabolic process	16	0.1130	4	12

GO:0019438	biological process	aromatic compound biosynthetic process	17	0.1410	1	2
GO:0031325	biological process	positive regulation of cellular metabolic process	18	0.1130	4	12
GO:0009893	biological process	positive regulation of metabolic process	18	0.1130	4	12
GO:0009896	biological process	positive regulation of catabolic process	18	0.1130	4	12
GO:0031329	biological process	regulation of cellular catabolic process	18	0.1130	4	12
GO:0031331	biological process	positive regulation of cellular catabolic process	18	0.1130	4	12
GO:0048522	biological process	positive regulation of cellular process	18	0.1130	4	12
GO:0032268	biological process	regulation of cellular protein metabolic process	19	0.1130	4	12
GO:0006417	biological process	regulation of translation	19	0.1130	4	12
GO:0032879	biological process	regulation of localization	20	0.1130	4	12
GO:0034248	biological process	regulation of cellular amide metabolic process	21	0.1130	4	12
GO:0034641	biological process	cellular nitrogen compound metabolic process	22	0.1410	1	2
GO:0034645	biological process	cellular macromolecule biosynthetic process	23	0.1410	1	2
GO:0034654	biological process	nucleobase-containing compound biosynthetic process	24	0.1410	1	2
GO:0035821	biological process	modification of morphology or physiology of other organism	25	0.1130	4	12
GO:0039604	biological process	suppression by virus of host translation	26	0.1130	4	12
GO:0019057	biological process	modulation by virus of host translation	26	0.1130	4	12
GO:0039606	biological process	suppression by virus of host translation initiation	26	0.1130	4	12
GO:0039611	biological process	suppression by virus of host translation initiation factor activity	26	0.1130	4	12
GO:0039690	biological process	positive stranded viral RNA replication	27	0.0494	4	12
GO:0019083	biological process	viral transcription	27	0.0494	4	42
GO:0039689	biological process	negative stranded viral RNA replication	27	0.0494	4	42
GO:0039694	biological process	viral RNA genome replication	27	0.0494	8	54
GO:0039696	biological process	RNA-templated viral transcription	28	0.0113	4	42
GO:0039697	biological process	negative stranded viral RNA transcription	29	0.0190	1	7

GO:0043170	biological process	macromolecule metabolic process	30	0.1410	1	2
GO:0044073	biological process	modulation by symbiont of host translation	31	0.1130	4	12
GO:0044092	biological process	negative regulation of molecular function	32	0.1130	4	12
GO:0044237	biological process	cellular metabolic process	33	0.1410	1	2
GO:0044238	biological process	primary metabolic process	34	0.1410	1	2
GO:0044249	biological process	cellular biosynthetic process	35	0.1410	1	2
GO:0044260	biological process	cellular macromolecule metabolic process	36	0.1410	1	2
GO:0044271	biological process	cellular nitrogen compound biosynthetic process	37	0.1410	1	2
GO:0044359	biological process	modulation of molecular function in other organism	38	0.1130	4	12
GO:0019048	biological process	modulation by virus of host morphology or physiology	38	0.1130	4	12
GO:0019054	biological process	modulation by virus of host process	38	0.1130	4	12
GO:0039506	biological process	modulation by virus of host molecular function	38	0.1130	4	12
GO:0039507	biological process	suppression by virus of host molecular function	38	0.1130	4	12
GO:0039519	biological process	modulation by virus of host autophagy	38	0.1130	4	12
GO:0039520	biological process	induction by virus of host autophagy	38	0.1130	4	12
GO:0039656	biological process	modulation by virus of host gene expression	38	0.1130	4	12
GO:0039657	biological process	suppression by virus of host gene expression	38	0.1130	4	12
GO:0044003	biological process	modification by symbiont of host morphology or physiology	38	0.1130	4	12
GO:0044068	biological process	modulation by symbiont of host cellular process	38	0.1130	4	12
GO:0044362	biological process	negative regulation of molecular function in other organism	38	0.1130	4	12
GO:0051817	biological process	modification of morphology or physiology of other organism involved in symbiotic interaction	38	0.1130	4	12
GO:0052055	biological process	modulation by symbiont of host molecular function	38	0.1130	4	12

GO:0052056	biological process	negative regulation by symbiont of host molecular function	38	0.1130	4	12
GO:0052204	biological process	negative regulation of molecular function in other organism involved in symbiotic interaction	38	0.1130	4	12
GO:0052205	biological process	modulation of molecular function in other organism involved in symbiotic interaction	38	0.1130	4	12
GO:0046483	biological process	heterocycle metabolic process	39	0.1410	1	2
GO:0048518	biological process	positive regulation of biological process	40	0.1130	4	12
GO:0048519	biological process	negative regulation of biological process	41	0.1130	4	12
GO:0050794	biological process	regulation of cellular process	42	0.1130	4	12
GO:0050789	biological process	regulation of biological process	42	0.1130	4	12
GO:0051246	biological process	regulation of protein metabolic process	43	0.1130	4	12
GO:0051701	biological process	interaction with host	44	0.0657	4	12
GO:0016032	biological process	viral process	44	0.0657	9	57
GO:0019079	biological process	viral genome replication	44	0.0657	8	54
GO:0044403	biological process	symbiosis, encompassing mutualism through parasitism	44	0.0657	9	57
GO:0044419	biological process	interspecies interaction between organisms	44	0.0657	9	57
GO:0046755	biological process	viral budding	44	0.0657	1	3
GO:0046765	biological process	viral budding from nuclear membrane	44	0.0657	1	3
GO:0051704	biological process	multi-organism process	45	0.0609	9	57
GO:0052038	biological process	modulation by symbiont of host intracellular transport	46	0.1130	4	12
GO:0039522	biological process	suppression by virus of host mRNA export from nucleus	46	0.1130	4	12
GO:0052230	biological process	modulation of intracellular transport in other organism involved in symbiotic interaction	47	0.1130	4	12
GO:0060341	biological process	regulation of cellular localization	48	0.1130	4	12

GO:0032386	biological process	regulation of intracellular transport	48	0.1130	4	12
GO:0051049	biological process	regulation of transport	48	0.1130	4	12
GO:0065007	biological process	biological regulation	49	0.1130	4	12
GO:0065009	biological process	regulation of molecular function	50	0.1130	4	12
GO:0071704	biological process	organic substance metabolic process	51	0.1410	1	2
GO:0071897	biological process	DNA biosynthetic process	52	0.1410	1	2
GO:0090304	biological process	nucleic acid metabolic process	53	0.1410	1	2
GO:1901360	biological process	organic cyclic compound metabolic process	54	0.1410	1	2
GO:1901362	biological process	organic cyclic compound biosynthetic process	55	0.1410	1	2
GO:1901576	biological process	organic substance biosynthetic process	56	0.1410	1	2
GO:2000112	biological process	regulation of cellular macromolecule biosynthetic process	57	0.1130	4	12
GO:0009889	biological process	regulation of biosynthetic process	57	0.1130	4	12
GO:0010468	biological process	regulation of gene expression	57	0.1130	4	12
GO:0010556	biological process	regulation of macromolecule biosynthetic process	57	0.1130	4	12
GO:0031323	biological process	regulation of cellular metabolic process	57	0.1130	4	12
GO:0031326	biological process	regulation of cellular biosynthetic process	57	0.1130	4	12
GO:0051171	biological process	regulation of nitrogen compound metabolic process	57	0.1130	4	12
GO:0060255	biological process	regulation of macromolecule metabolic process	57	0.1130	4	12
GO:0080090	biological process	regulation of primary metabolic process	57	0.1130	4	12
GO:0001681	molecular function	sialate O-acetylesterase activity	58	0.2260	1	2
GO:0008126	molecular function	acetylesterase activity	58	0.2260	1	2
GO:0003674	molecular function	molecular function	59	0.1195	4	11
GO:0003824	molecular function	catalytic activity	60	0.1480	3	9
GO:0004518	molecular function	nuclease activity	61	0.1930	1	2
GO:0004519	molecular function	endonuclease activity	61	0.1930	1	2

GO:0005488	molecular function	binding	62	0.0340	1	2
GO:0016740	molecular function	transferase activity	63	0.0250	1	5
GO:0016772	molecular function	transferase activity, transferring phosphorus-containing groups	64	0.0250	1	5
GO:0016779	molecular function	nucleotidyltransferase activity	65	0.0250	1	5
GO:0016787	molecular function	hydrolase activity	66	0.2095	2	4
GO:0016788	molecular function	hydrolase activity, acting on ester bonds	67	0.2095	2	4
GO:0034062	molecular function	5'-3' RNA polymerase activity	68	0.0250	1	5
GO:0003968	molecular function	RNA-directed 5'-3' RNA polymerase activity	68	0.0250	1	5
GO:0097747	molecular function	RNA polymerase activity	68	0.0250	1	5
GO:0034338	molecular function	short-chain carboxylesterase activity	69	0.2260	1	2
GO:0052689	molecular function	carboxylic ester hydrolase activity	70	0.2260	1	2

Table S2: Structural coverage of subVOG mRNAs associated with GO terms. The GO terms are clustered into functionally similar groups using Revigo. All GO terms without regard on evidence codes were used for the clustering. A color coded version of the table is provided online at http://subvogs.bio.wzw.tum.de/sup/table_s2.html.

GO term	Ontology	Description	Revigo cluster	Mean structural coverage	Number of subVOGs	Number of proteins
GO:0001505	biological process	regulation of neurotransmitter levels	1	0.1686	20	61
GO:0001510	biological process	RNA methylation	2	0.0810	1	4
GO:0001906	biological process	cell killing	3	0.2271	29	148
GO:0002097	biological process	tRNA wobble base modification	4	0.0620	1	3
GO:0002376	biological process	immune system process	5	0.1453	4	11
GO:0002920	biological process	regulation of humoral immune response	6	0.1973	2	5
GO:0002682	biological process	regulation of immune system process	6	0.1973	94	316
GO:0002697	biological process	regulation of immune effector process	6	0.1973	2	5
GO:0050690	biological process	regulation of defense response to virus by virus	6	0.1973	95	319
GO:0005975	biological process	carbohydrate metabolic process	7	0.1705	32	116
GO:0005976	biological process	polysaccharide metabolic process	8	0.1778	4	10
GO:0005996	biological process	monosaccharide metabolic process	9	0.0170	1	11
GO:0006002	biological process	fructose 6-phosphate metabolic process	10	0.1400	1	7
GO:0006022	biological process	aminoglycan metabolic process	11	0.1659	155	915
GO:0006040	biological process	amino sugar metabolic process	12	0.1625	31	96
GO:0006081	biological process	cellular aldehyde metabolic process	13	0.1735	2	13
GO:0006082	biological process	organic acid metabolic process	14	0.1519	73	274
GO:0006091	biological process	generation of precursor metabolites and energy	15	0.1954	13	92

GO:0006109	biological process	regulation of carbohydrate metabolic process	16	0.3035	2	4
GO:0006139	biological process	nucleobase-containing compound metabolic process	17	0.1306	1774	8662
GO:0006259	biological process	DNA metabolic process	18	0.1301	1014	5301
GO:0006260	biological process	DNA replication	19	0.1146	516	2643
GO:0006261	biological process	DNA-dependent DNA replication	20	0.1142	121	382
GO:0006265	biological process	DNA topological change	21	0.1377	43	228
GO:0006266	biological process	DNA ligation	22	0.1471	11	55
GO:0006270	biological process	DNA replication initiation	23	0.2598	12	35
GO:0006284	biological process	base-excision repair	24	0.0986	14	57
GO:0006289	biological process	nucleotide-excision repair	25	0.0723	4	13
GO:0006298	biological process	mismatch repair	26	0.0425	4	10
GO:0006304	biological process	DNA modification	27	0.1373	36	166
GO:0006305	biological process	DNA alkylation	28	0.1230	32	153
GO:0044728	biological process	DNA methylation or demethylation	28	0.1230	32	153
GO:0006310	biological process	DNA recombination	29	0.1468	208	1044
GO:0006313	biological process	transposition, DNA-mediated	30	0.2166	10	26
GO:0006325	biological process	chromatin organization	31	0.3430	1	2
GO:0006352	biological process	DNA-templated transcription, initiation	32	0.1353	35	140
GO:0006353	biological process	DNA-templated transcription, termination	33	0.1983	3	9
GO:0006370	biological process	7-methylguanosine mRNA capping	34	0.1916	14	48
GO:0006396	biological process	RNA processing	35	0.1124	83	374
GO:0006402	biological process	mRNA catabolic process	36	0.1883	2	4
GO:0006308	biological process	DNA catabolic process	36	0.1883	4	17
GO:0006397	biological process	mRNA processing	36	0.1883	19	76
GO:0006401	biological process	RNA catabolic process	36	0.1883	7	22
GO:0009143	biological process	nucleoside triphosphate catabolic process	36	0.1883	2	4

GO:0009166	biological process	nucleotide catabolic process	36	0.1883	13	53
GO:0009264	biological process	deoxyribonucleotide catabolic process	36	0.1883	11	45
GO:0034661	biological process	ncRNA catabolic process	36	0.1883	5	18
GO:0006413	biological process	translational initiation	37	0.1225	2	12
GO:0006420	biological process	arginyl-tRNA aminoacylation	38	0.0070	1	3
GO:0006421	biological process	asparaginyl-tRNA aminoacylation	39	0.0720	1	2
GO:0006428	biological process	isoleucyl-tRNA aminoacylation	40	0.0610	1	3
GO:0006431	biological process	methionyl-tRNA aminoacylation	41	0.1090	1	2
GO:0006457	biological process	protein folding	42	0.2132	18	91
GO:0006487	biological process	protein N-linked glycosylation	43	0.0180	1	4
GO:0006497	biological process	protein lipidation	44	0.3058	4	10
GO:0006508	biological process	proteolysis	45	0.1755	27	98
GO:0006518	biological process	peptide metabolic process	46	0.2159	1	2
GO:0043604	biological process	amide biosynthetic process	46	0.2159	53	196
GO:0006541	biological process	glutamine metabolic process	47	0.1602	6	23
GO:0006560	biological process	proline metabolic process	48	0.0175	1	9
GO:0009084	biological process	glutamine family amino acid biosynthetic process	48	0.0175	1	3
GO:0006575	biological process	cellular modified amino acid metabolic process	49	0.1569	50	182
GO:0006629	biological process	lipid metabolic process	50	0.1251	31	117
GO:0006725	biological process	cellular aromatic compound metabolic process	51	0.1318	1830	8877
GO:0006730	biological process	one-carbon metabolic process	52	0.2320	1	2
GO:0006739	biological process	NADP metabolic process	53	0.1735	2	13
GO:0006766	biological process	vitamin metabolic process	54	0.2650	5	17
GO:0006790	biological process	sulfur compound metabolic process	55	0.4793	4	12
GO:0006793	biological process	phosphorus metabolic process	56	0.1545	235	1412
GO:0006796	biological process	phosphate-containing compound metabolic process	57	0.1541	233	1407

GO:0019637	biological process	organophosphate metabolic process	57	0.1541	233	1407
GO:0006807	biological process	nitrogen compound metabolic process	58	0.1364	2061	10074
GO:0006811	biological process	ion transport	59	0.1544	5	27
GO:0006820	biological process	anion transport	60	0.0828	4	25
GO:0034220	biological process	ion transmembrane transport	60	0.0828	1	15
GO:0006869	biological process	lipid transport	61	0.1950	2	4
GO:0006898	biological process	receptor-mediated endocytosis	62	0.1322	5	11
GO:0006950	biological process	response to stress	63	0.1536	205	1106
GO:0006952	biological process	defense response	64	0.2462	19	82
GO:0006974	biological process	cellular response to DNA damage stimulus	65	0.1443	171	959
GO:0006979	biological process	response to oxidative stress	66	0.1005	2	6
GO:0006996	biological process	organelle organization	67	0.1203	102	562
GO:0007049	biological process	cell cycle	68	0.0770	3	9
GO:0007154	biological process	cell communication	69	0.1829	8	37
GO:0007155	biological process	cell adhesion	70	0.0995	4	10
GO:0007166	biological process	cell surface receptor signaling pathway	71	0.2150	1	2
GO:0007264	biological process	small GTPase mediated signal transduction	72	0.2020	1	2
GO:0008150	biological process	biological process	73	0.1392	3435	16134
GO:0008152	biological process	metabolic process	74	0.1385	2201	10701
GO:0008202	biological process	steroid metabolic process	75	0.1166	5	26
GO:0008216	biological process	spermidine metabolic process	76	0.2253	6	21
GO:0008217	biological process	regulation of blood pressure	77	0.1750	1	2
GO:0008380	biological process	RNA splicing	78	0.1175	2	4
GO:0008654	biological process	phospholipid biosynthetic process	79	0.2727	1	3
GO:0006644	biological process	phospholipid metabolic process	79	0.2727	4	10
GO:0008610	biological process	lipid biosynthetic process	79	0.2727	6	29
GO:0044255	biological process	cellular lipid metabolic process	79	0.2727	5	12

GO:0009056	biological process	catabolic process	80	0.1604	162	951
GO:0009058	biological process	biosynthetic process	81	0.1287	1290	6001
GO:0009059	biological process	macromolecule biosynthetic process	82	0.1197	1019	4509
GO:0009065	biological process	glutamine family amino acid catabolic process	83	0.0180	1	9
GO:0009066	biological process	aspartate family amino acid metabolic process	84	0.2480	4	17
GO:0009069	biological process	serine family amino acid metabolic process	85	0.1732	21	64
GO:0009071	biological process	serine family amino acid catabolic process	86	0.2716	1	3
GO:0006563	biological process	L-serine metabolic process	86	0.2716	2	5
GO:0009070	biological process	serine family amino acid biosynthetic process	86	0.2716	20	61
GO:0019264	biological process	glycine biosynthetic process from serine	86	0.2716	1	2
GO:0009072	biological process	aromatic amino acid family metabolic process	87	0.1325	2	15
GO:0009112	biological process	nucleobase metabolic process	88	0.1240	1	3
GO:0009129	biological process	pyrimidine nucleoside monophosphate metabolic process	89	0.1513	83	497
GO:0009132	biological process	nucleoside diphosphate metabolic process	90	0.1563	6	15
GO:0009141	biological process	nucleoside triphosphate metabolic process	91	0.0846	41	165
GO:0009126	biological process	purine nucleoside monophosphate metabolic process	91	0.0846	2	15
GO:0009142	biological process	nucleoside triphosphate biosynthetic process	91	0.0846	6	26
GO:0009152	biological process	purine ribonucleotide biosynthetic process	91	0.0846	3	17
GO:0009161	biological process	ribonucleoside monophosphate metabolic process	91	0.0846	12	67
GO:0009259	biological process	ribonucleotide metabolic process	91	0.0846	14	72
GO:0046390	biological process	ribose phosphate biosynthetic process	91	0.0846	13	69
GO:0009144	biological process	purine nucleoside triphosphate metabolic process	92	0.1564	1	2
GO:0006753	biological process	nucleoside phosphate metabolic process	92	0.1564	227	1388
GO:0009117	biological process	nucleotide metabolic process	92	0.1564	225	1384
GO:0009123	biological process	nucleoside monophosphate metabolic process	92	0.1564	85	512

GO:0009199	biological process	ribonucleoside triphosphate metabolic process	92	0.1564	1	2
GO:0090407	biological process	organophosphate biosynthetic process	92	0.1564	163	1101
GO:1901293	biological process	nucleoside phosphate biosynthetic process	92	0.1564	161	1096
GO:0009147	biological process	pyrimidine nucleoside triphosphate metabolic process	93	0.1118	39	161
GO:0009162	biological process	deoxyribonucleoside monophosphate metabolic process	94	0.1640	73	445
GO:0009173	biological process	pyrimidine ribonucleoside monophosphate metabolic process	95	0.0587	10	52
GO:0009200	biological process	deoxyribonucleoside triphosphate metabolic process	96	0.1103	38	159
GO:0009202	biological process	deoxyribonucleoside triphosphate biosynthetic process	97	0.0756	5	24
GO:0009218	biological process	pyrimidine ribonucleotide metabolic process	98	0.0687	11	54
GO:0009225	biological process	nucleotide-sugar metabolic process	99	0.0916	5	38
GO:0009226	biological process	nucleotide-sugar biosynthetic process	100	0.0511	4	16
GO:0009298	biological process	GDP-mannose biosynthetic process	100	0.0511	1	4
GO:0019673	biological process	GDP-mannose metabolic process	100	0.0511	2	26
GO:0009235	biological process	cobalamin metabolic process	101	0.2049	4	13
GO:0006778	biological process	porphyrin-containing compound metabolic process	101	0.2049	1	5
GO:0015939	biological process	pantothenate metabolic process	101	0.2049	1	4
GO:0033014	biological process	tetrapyrrole biosynthetic process	101	0.2049	7	28
GO:0042559	biological process	pteridine-containing compound biosynthetic process	101	0.2049	47	173
GO:0046653	biological process	tetrahydrofolate metabolic process	101	0.2049	47	172
GO:0046654	biological process	tetrahydrofolate biosynthetic process	101	0.2049	46	170
GO:0009254	biological process	peptidoglycan turnover	102	0.1764	10	45
GO:0006026	biological process	aminoglycan catabolic process	102	0.1764	120	793

GO:0006027	biological process	glycosaminoglycan catabolic process	102	0.1764	114	775
GO:0030203	biological process	glycosaminoglycan metabolic process	102	0.1764	125	822
GO:0009262	biological process	deoxyribonucleotide metabolic process	103	0.1488	162	939
GO:0009263	biological process	deoxyribonucleotide biosynthetic process	104	0.1538	118	759
GO:0009308	biological process	amine metabolic process	105	0.1878	10	35
GO:0009372	biological process	quorum sensing	106	0.0340	1	2
GO:0009411	biological process	response to UV	107	0.0840	2	7
GO:0009445	biological process	putrescine metabolic process	108	0.0960	1	4
GO:0009451	biological process	RNA modification	109	0.0877	7	30
GO:0009605	biological process	response to external stimulus	110	0.1497	126	461
GO:0009607	biological process	response to biotic stimulus	111	0.1499	115	410
GO:0009628	biological process	response to abiotic stimulus	112	0.0957	3	9
GO:0009653	biological process	anatomical structure morphogenesis	113	0.0842	3	11
GO:0032989	biological process	cellular component morphogenesis	113	0.0842	1	5
GO:0009892	biological process	negative regulation of metabolic process	114	0.1758	44	150
GO:0009890	biological process	negative regulation of biosynthetic process	114	0.1758	12	44
GO:0010629	biological process	negative regulation of gene expression	114	0.1758	41	142
GO:0010648	biological process	negative regulation of cell communication	114	0.1758	68	225
GO:0010941	biological process	regulation of cell death	114	0.1758	45	147
GO:0023057	biological process	negative regulation of signaling	114	0.1758	68	225
GO:0031324	biological process	negative regulation of cellular metabolic process	114	0.1758	15	52
GO:0043066	biological process	negative regulation of apoptotic process	114	0.1758	8	21
GO:0045934	biological process	negative regulation of nucleobase-containing compound metabolic process	114	0.1758	11	32
GO:0048523	biological process	negative regulation of cellular process	114	0.1758	113	404
GO:0048585	biological process	negative regulation of response to stimulus	114	0.1758	92	309

GO:0051172	biological process	negative regulation of nitrogen compound metabolic process	114	0.1758	15	52
GO:0060548	biological process	negative regulation of cell death	114	0.1758	13	32
GO:0080134	biological process	regulation of response to stress	114	0.1758	90	304
GO:0009894	biological process	regulation of catabolic process	115	0.1311	51	167
GO:0009987	biological process	cellular process	116	0.1397	2291	10962
GO:0009991	biological process	response to extracellular stimulus	117	0.1825	8	37
GO:0042330	biological process	taxis	117	0.1825	3	6
GO:0071496	biological process	cellular response to external stimulus	117	0.1825	8	37
GO:0010207	biological process	photosystem II assembly	118	0.1789	1	3
GO:0009772	biological process	photosynthetic electron transport in photosystem II	118	0.1789	12	90
GO:0010468	biological process	regulation of gene expression	119	0.1535	226	893
GO:0009889	biological process	regulation of biosynthetic process	119	0.1535	232	901
GO:0019219	biological process	regulation of nucleobase-containing compound metabolic process	119	0.1535	242	928
GO:0031323	biological process	regulation of cellular metabolic process	119	0.1535	288	1090
GO:0051171	biological process	regulation of nitrogen compound metabolic process	119	0.1535	250	958
GO:0051252	biological process	regulation of RNA metabolic process	119	0.1535	205	781
GO:0060255	biological process	regulation of macromolecule metabolic process	119	0.1535	270	1058
GO:0080090	biological process	regulation of primary metabolic process	119	0.1535	252	962
GO:1903506	biological process	regulation of nucleic acid-templated transcription	119	0.1535	173	668
GO:2000112	biological process	regulation of cellular macromolecule biosynthetic process	119	0.1535	211	827
GO:2001141	biological process	regulation of RNA biosynthetic process	119	0.1535	194	742
GO:0010469	biological process	regulation of receptor activity	120	0.3270	2	5
GO:0010506	biological process	regulation of autophagy	121	0.1397	46	149

GO:0010604	biological process	positive regulation of macromolecule metabolic process	122	0.1583	35	156
GO:0009891	biological process	positive regulation of biosynthetic process	122	0.1583	7	32
GO:0009893	biological process	positive regulation of metabolic process	122	0.1583	76	295
GO:0010628	biological process	positive regulation of gene expression	122	0.1583	24	126
GO:0051054	biological process	positive regulation of DNA metabolic process	122	0.1583	1	2
GO:0051173	biological process	positive regulation of nitrogen compound metabolic process	122	0.1583	17	60
GO:0051254	biological process	positive regulation of RNA metabolic process	122	0.1583	16	58
GO:1902680	biological process	positive regulation of RNA biosynthetic process	122	0.1583	6	30
GO:0010608	biological process	posttranscriptional regulation of gene expression	123	0.0750	1	12
GO:0010646	biological process	regulation of cell communication	124	0.1573	73	238
GO:0015074	biological process	DNA integration	125	0.1498	123	688
GO:0015698	biological process	inorganic anion transport	126	0.0680	2	21
GO:0015711	biological process	organic anion transport	127	0.1950	2	4
GO:0015849	biological process	organic acid transport	128	0.1950	2	4
GO:0015931	biological process	nucleobase-containing compound transport	129	0.1123	15	99
GO:0015979	biological process	photosynthesis	130	0.2910	2	5
GO:0016042	biological process	lipid catabolic process	131	0.0566	7	28
GO:0016051	biological process	carbohydrate biosynthetic process	132	0.1778	4	10
GO:0016070	biological process	RNA metabolic process	133	0.1198	548	2031
GO:0016071	biological process	mRNA metabolic process	134	0.2031	21	80
GO:0016072	biological process	rRNA metabolic process	135	0.0925	6	20
GO:0006364	biological process	rRNA processing	135	0.0925	1	2
GO:0006399	biological process	tRNA metabolic process	135	0.0925	19	77
GO:0006400	biological process	tRNA modification	135	0.0925	6	26
GO:0008033	biological process	tRNA processing	135	0.0925	12	56

GO:0034470	biological process	ncRNA processing	135	0.0925	13	58
GO:0016192	biological process	vesicle-mediated transport	136	0.0731	10	110
GO:0016310	biological process	phosphorylation	137	0.0230	1	3
GO:0016539	biological process	intein-mediated protein splicing	138	0.2358	9	25
GO:0016485	biological process	protein processing	138	0.2358	17	64
GO:0030908	biological process	protein splicing	138	0.2358	10	28
GO:0097428	biological process	protein maturation by iron-sulfur cluster transfer	138	0.2358	1	2
GO:0017006	biological process	protein-tetrapyrrole linkage	139	0.2212	5	26
GO:0017144	biological process	drug metabolic process	140	0.1726	53	165
GO:0018126	biological process	protein hydroxylation	141	0.3048	4	21
GO:0018130	biological process	heterocycle biosynthetic process	142	0.1328	746	3266
GO:0018142	biological process	protein-DNA covalent cross-linking	143	0.1118	36	104
GO:0018144	biological process	RNA-protein covalent cross-linking	143	0.1118	123	409
GO:0018143	biological process	nucleic acid-protein covalent cross-linking	144	0.1071	159	513
GO:0018193	biological process	peptidyl-amino acid modification	145	0.3115	6	26
GO:0018197	biological process	peptidyl-aspartic acid modification	146	0.3048	4	21
GO:0018298	biological process	protein-chromophore linkage	147	0.1190	1	2
GO:0018904	biological process	ether metabolic process	148	0.1333	4	15
GO:0019082	biological process	viral protein processing	149	0.1817	22	55
GO:0019083	biological process	viral transcription	150	0.0955	29	110
GO:0039686	biological process	bidirectional double-stranded viral DNA replication	150	0.0955	20	135
GO:0039689	biological process	negative stranded viral RNA replication	150	0.0955	1	2
GO:0039693	biological process	viral DNA genome replication	150	0.0955	70	353
GO:0039694	biological process	viral RNA genome replication	150	0.0955	306	997
GO:0039696	biological process	RNA-templated viral transcription	150	0.0955	1	2
GO:0046782	biological process	regulation of viral transcription	150	0.0955	29	127
GO:0075526	biological process	cap snatching	150	0.0955	1	2

GO:0019222	biological process	regulation of metabolic process	151	0.1509	310	1195
GO:0019438	biological process	aromatic compound biosynthetic process	152	0.1328	747	3278
GO:0019439	biological process	aromatic compound catabolic process	153	0.1733	26	96
GO:0043632	biological process	modification-dependent macromolecule catabolic process	153	0.1733	4	12
GO:0044265	biological process	cellular macromolecule catabolic process	153	0.1733	15	51
GO:0044270	biological process	cellular nitrogen compound catabolic process	153	0.1733	26	96
GO:0046700	biological process	heterocycle catabolic process	153	0.1733	27	105
GO:0051603	biological process	proteolysis involved in cellular protein catabolic process	153	0.1733	4	12
GO:0019538	biological process	protein metabolic process	154	0.1274	212	703
GO:0019692	biological process	deoxyribose phosphate metabolic process	155	0.1462	117	619
GO:0019693	biological process	ribose phosphate metabolic process	156	0.0937	16	85
GO:0019725	biological process	cellular homeostasis	157	0.1554	60	297
GO:0042592	biological process	homeostatic process	157	0.1554	62	328
GO:0045454	biological process	cell redox homeostasis	157	0.1554	57	283
GO:0019835	biological process	cytolysis	158	0.2859	61	313
GO:0022607	biological process	cellular component assembly	159	0.1566	66	163
GO:0022610	biological process	biological adhesion	160	0.1201	130	450
GO:0023051	biological process	regulation of signaling	161	0.1573	73	238
GO:0030154	biological process	cell differentiation	162	0.1932	1	3
GO:0048869	biological process	cellular developmental process	162	0.1932	2	8
GO:0030162	biological process	regulation of proteolysis	163	0.1484	5	13
GO:0031399	biological process	regulation of protein modification process	163	0.1484	24	74
GO:0032268	biological process	regulation of cellular protein metabolic process	163	0.1484	28	94
GO:0030163	biological process	protein catabolic process	164	0.0560	3	10
GO:0030258	biological process	lipid modification	165	0.0230	1	3

GO:0031123	biological process	RNA 3'-end processing	166	0.1190	3	18
GO:0031163	biological process	metallo-sulfur cluster assembly	167	0.4105	2	7
GO:0031341	biological process	regulation of cell killing	168	0.3430	9	38
GO:0031342	biological process	negative regulation of cell killing	168	0.3430	8	36
GO:0031343	biological process	positive regulation of cell killing	169	0.0700	1	2
GO:0051715	biological process	cytolysis in other organism	169	0.0700	1	19
GO:0031365	biological process	N-terminal protein amino acid modification	170	0.3058	4	10
GO:0031638	biological process	zymogen activation	171	0.1090	1	6
GO:0032196	biological process	transposition	172	0.1998	13	43
GO:0032259	biological process	methylation	173	0.1218	33	157
GO:0032409	biological process	regulation of transporter activity	174	0.3579	8	36
GO:0032502	biological process	developmental process	175	0.1390	4	14
GO:0032787	biological process	monocarboxylic acid metabolic process	176	0.2230	6	23
GO:0032879	biological process	regulation of localization	177	0.3579	8	36
GO:0033013	biological process	tetrapyrrole metabolic process	178	0.2285	8	33
GO:0033036	biological process	macromolecule localization	179	0.0210	1	6
GO:0033567	biological process	DNA replication, Okazaki fragment processing	180	0.1190	10	26
GO:0033865	biological process	nucleoside bisphosphate metabolic process	181	0.2530	1	3
GO:0034248	biological process	regulation of cellular amide metabolic process	182	0.0750	1	12
GO:0034404	biological process	nucleobase-containing small molecule biosynthetic process	183	0.1719	18	67
GO:0008652	biological process	cellular amino acid biosynthetic process	183	0.1719	27	96
GO:0016053	biological process	organic acid biosynthetic process	183	0.1719	57	217
GO:1901607	biological process	alpha-amino acid biosynthetic process	183	0.1719	25	81
GO:0034622	biological process	cellular macromolecular complex assembly	184	0.0589	8	26
GO:0017004	biological process	cytochrome complex assembly	184	0.0589	1	9
GO:0033108	biological process	mitochondrial respiratory chain complex assembly	184	0.0589	1	9

GO:0051260	biological process	protein homooligomerization	184	0.0589	6	19
GO:0070897	biological process	DNA-templated transcriptional preinitiation complex assembly	184	0.0589	5	12
GO:0034641	biological process	cellular nitrogen compound metabolic process	185	0.1324	1843	8915
GO:0034645	biological process	cellular macromolecule biosynthetic process	186	0.1187	979	4356
GO:0034654	biological process	nucleobase-containing compound biosynthetic process	187	0.1226	694	3073
GO:0032774	biological process	RNA biosynthetic process	187	0.1226	484	1764
GO:0097659	biological process	nucleic acid-templated transcription	187	0.1226	446	1615
GO:0034660	biological process	ncRNA metabolic process	188	0.1032	25	97
GO:0035556	biological process	intracellular signal transduction	189	0.0963	3	8
GO:0007165	biological process	signal transduction	189	0.0963	4	10
GO:0035821	biological process	modification of morphology or physiology of other organism	190	0.1610	228	810
GO:0036211	biological process	protein modification process	191	0.1202	186	613
GO:0036260	biological process	RNA capping	192	0.1842	15	52
GO:0039514	biological process	suppression by virus of host JAK-STAT cascade	193	0.1206	24	86
GO:0039644	biological process	suppression by virus of host NF-kappaB transcription factor activity	194	0.1839	5	10
GO:0019048	biological process	modulation by virus of host morphology or physiology	194	0.1839	180	687
GO:0039548	biological process	suppression by virus of host IRF3 activity	194	0.1839	1	2
GO:0039579	biological process	suppression by virus of host ISG15 activity	194	0.1839	5	10
GO:0039648	biological process	modulation by virus of host protein ubiquitination	194	0.1839	27	79
GO:0039656	biological process	modulation by virus of host gene expression	194	0.1839	27	94
GO:0044068	biological process	modulation by symbiont of host cellular process	194	0.1839	122	412
GO:0044359	biological process	modulation of molecular function in other organism	194	0.1839	53	169

GO:0044362	biological process	negative regulation of molecular function in other organism	194	0.1839	32	102
GO:0044364	biological process	disruption of cells of other organism	194	0.1839	29	148
GO:0044501	biological process	modulation of signal transduction in other organism	194	0.1839	68	225
GO:0051673	biological process	membrane disruption in other organism	194	0.1839	47	104
GO:0051807	biological process	evasion or tolerance of defense response of other organism involved in symbiotic interaction	194	0.1839	95	319
GO:0051817	biological process	modification of morphology or physiology of other organism involved in symbiotic interaction	194	0.1839	227	791
GO:0051818	biological process	disruption of cells of other organism involved in symbiotic interaction	194	0.1839	28	129
GO:0051833	biological process	suppression of defenses of other organism involved in symbiotic interaction	194	0.1839	90	304
GO:0052185	biological process	modification of structure of other organism involved in symbiotic interaction	194	0.1839	47	104
GO:0052255	biological process	modulation by organism of defense response of other organism involved in symbiotic interaction	194	0.1839	90	304
GO:0052312	biological process	modulation of transcription in other organism involved in symbiotic interaction	194	0.1839	22	76
GO:0052490	biological process	negative regulation by organism of programmed cell death in other organism involved in symbiotic interaction	194	0.1839	10	24
GO:0052564	biological process	response to immune response of other organism involved in symbiotic interaction	194	0.1839	95	319
GO:0039654	biological process	fusion of virus membrane with host endosome membrane	195	0.1062	33	117
GO:0019064	biological process	fusion of virus membrane with host plasma membrane	195	0.1062	39	143
GO:0019069	biological process	viral capsid assembly	195	0.1062	53	308

GO:0019073	biological process	viral DNA genome packaging	195	0.1062	50	185
GO:0046755	biological process	viral budding	195	0.1062	25	94
GO:0046760	biological process	viral budding from Golgi membrane	195	0.1062	7	20
GO:0046765	biological process	viral budding from nuclear membrane	195	0.1062	12	61
GO:0039664	biological process	lysis of host organelle involved in viral entry into host cell	196	0.1385	6	22
GO:0039665	biological process	permeabilization of host organelle membrane involved in viral entry into host cell	197	0.0100	1	3
GO:0039666	biological process	virion attachment to host cell pilus	198	0.3980	2	4
GO:0046813	biological process	receptor-mediated virion attachment to host cell	198	0.3980	1	2
GO:0039667	biological process	viral entry into host cell via pilus retraction	199	0.2051	1	2
GO:0009405	biological process	pathogenesis	199	0.2051	32	137
GO:0016032	biological process	viral process	199	0.2051	1152	4827
GO:0032897	biological process	negative regulation of viral transcription	199	0.2051	7	36
GO:0035890	biological process	exit from host	199	0.2051	128	613
GO:0043901	biological process	negative regulation of multi-organism process	199	0.2051	15	72
GO:0050792	biological process	regulation of viral process	199	0.2051	39	169
GO:0051701	biological process	interaction with host	199	0.2051	463	1942
GO:0051828	biological process	entry into other organism involved in symbiotic interaction	199	0.2051	155	589
GO:0052192	biological process	movement in environment of other organism involved in symbiotic interaction	199	0.2051	164	818
GO:0075136	biological process	response to host	199	0.2051	98	335
GO:1903900	biological process	regulation of viral life cycle	199	0.2051	8	36
GO:0039678	biological process	viral genome ejection through host cell envelope	200	0.2386	34	187
GO:0039687	biological process	viral DNA strand displacement replication	201	0.1455	2	4
GO:0039702	biological process	viral budding via host ESCRT complex	202	0.1675	2	4
GO:0019072	biological process	viral genome packaging	202	0.1675	84	336

GO:0040011	biological process	locomotion	203	0.1708	173	841
GO:0040012	biological process	regulation of locomotion	204	0.3579	8	36
GO:0042135	biological process	neurotransmitter catabolic process	205	0.2995	1	3
GO:0042136	biological process	neurotransmitter biosynthetic process	205	0.2995	19	58
GO:0042221	biological process	response to chemical	206	0.1095	4	22
GO:0042357	biological process	thiamine diphosphate metabolic process	207	0.3225	1	2
GO:0000096	biological process	sulfur amino acid metabolic process	207	0.3225	1	3
GO:0006213	biological process	pyrimidine nucleoside metabolic process	207	0.3225	1	2
GO:0006220	biological process	pyrimidine nucleotide metabolic process	207	0.3225	144	759
GO:0006221	biological process	pyrimidine nucleotide biosynthetic process	207	0.3225	96	540
GO:0044272	biological process	sulfur compound biosynthetic process	207	0.3225	2	5
GO:0072528	biological process	pyrimidine-containing compound biosynthetic process	207	0.3225	98	545
GO:0042398	biological process	cellular modified amino acid biosynthetic process	208	0.1557	48	177
GO:0042440	biological process	pigment metabolic process	209	0.2850	1	5
GO:0042558	biological process	pteridine-containing compound metabolic process	210	0.1566	49	178
GO:0042737	biological process	drug catabolic process	211	0.2303	7	21
GO:0042886	biological process	amide transport	212	0.0210	1	6
GO:0043038	biological process	amino acid activation	213	0.0936	7	21
GO:0043086	biological process	negative regulation of catalytic activity	214	0.0923	3	8
GO:0043170	biological process	macromolecule metabolic process	215	0.1328	1754	8370
GO:0043412	biological process	macromolecule modification	216	0.1219	229	809
GO:0043543	biological process	protein acylation	217	0.3058	4	10
GO:0043603	biological process	cellular amide metabolic process	218	0.1655	55	201
GO:0043648	biological process	dicarboxylic acid metabolic process	219	0.0480	1	5
GO:0043900	biological process	regulation of multi-organism process	220	0.1689	40	171
GO:0043902	biological process	positive regulation of multi-organism process	221	0.0900	1	2

GO:0043933	biological process	macromolecular complex subunit organization	222	0.1486	64	156
GO:0043934	biological process	sporulation	223	0.2370	1	3
GO:0044036	biological process	cell wall macromolecule metabolic process	224	0.1932	72	369
GO:0044092	biological process	negative regulation of molecular function	225	0.1607	43	146
GO:0050790	biological process	regulation of catalytic activity	225	0.1607	29	87
GO:0051336	biological process	regulation of hydrolase activity	225	0.1607	5	13
GO:0051338	biological process	regulation of transferase activity	225	0.1607	24	74
GO:0044237	biological process	cellular metabolic process	226	0.1368	2010	9679
GO:0044238	biological process	primary metabolic process	227	0.1337	1923	9214
GO:0044249	biological process	cellular biosynthetic process	228	0.1281	1265	5912
GO:0044260	biological process	cellular macromolecule metabolic process	229	0.1322	1554	7346
GO:0044262	biological process	cellular carbohydrate metabolic process	230	0.1515	2	6
GO:0044267	biological process	cellular protein metabolic process	231	0.1210	187	615
GO:0044271	biological process	cellular nitrogen compound biosynthetic process	232	0.1344	762	3324
GO:0044281	biological process	small molecule metabolic process	233	0.1492	316	1757
GO:0044282	biological process	small molecule catabolic process	234	0.1943	2	12
GO:0009057	biological process	macromolecule catabolic process	234	0.1943	138	854
GO:0044248	biological process	cellular catabolic process	234	0.1943	105	491
GO:0044283	biological process	small molecule biosynthetic process	235	0.1754	79	297
GO:0044419	biological process	interspecies interaction between organisms	236	0.1143	1197	5025
GO:0019058	biological process	viral life cycle	236	0.1143	37	148
GO:0019068	biological process	virion assembly	236	0.1143	28	144
GO:0019079	biological process	viral genome replication	236	0.1143	420	1514
GO:0044403	biological process	symbiosis, encompassing mutualism through parasitism	236	0.1143	1153	4829
GO:0044764	biological process	multi-organism cellular process	236	0.1143	75	283

GO:0044531	biological process	modulation of programmed cell death in other organism	237	0.1325	34	112
GO:0045229	biological process	external encapsulating structure organization	238	0.2740	1	2
GO:0046116	biological process	queuosine metabolic process	239	0.2065	2	8
GO:0046349	biological process	amino sugar biosynthetic process	240	0.1915	2	5
GO:0006047	biological process	UDP-N-acetylglucosamine metabolic process	240	0.1915	2	5
GO:0046348	biological process	amino sugar catabolic process	240	0.1915	6	18
GO:1901071	biological process	glucosamine-containing compound metabolic process	240	0.1915	29	91
GO:0046385	biological process	deoxyribose phosphate biosynthetic process	241	0.1582	84	484
GO:0046434	biological process	organophosphate catabolic process	242	0.1915	15	57
GO:0046483	biological process	heterocycle metabolic process	243	0.1317	1829	8871
GO:0046486	biological process	glycerolipid metabolic process	244	0.0230	1	3
GO:0046753	biological process	non-lytic viral release	245	0.2064	7	15
GO:0046903	biological process	secretion	246	0.1950	2	4
GO:0048017	biological process	inositol lipid-mediated signaling	247	0.0230	1	3
GO:0048518	biological process	positive regulation of biological process	248	0.1457	77	297
GO:0048519	biological process	negative regulation of biological process	249	0.1744	175	646
GO:0048522	biological process	positive regulation of cellular process	250	0.1176	59	201
GO:0010942	biological process	positive regulation of cell death	250	0.1176	1	2
GO:0031325	biological process	positive regulation of cellular metabolic process	250	0.1176	58	199
GO:0048583	biological process	regulation of response to stimulus	251	0.1493	99	329
GO:0048646	biological process	anatomical structure formation involved in morphogenesis	252	0.2370	1	3
GO:0048878	biological process	chemical homeostasis	253	0.0636	3	14
GO:0060249	biological process	anatomical structure homeostasis	253	0.0636	2	31
GO:0050684	biological process	regulation of mRNA processing	254	0.1944	6	21
GO:0061013	biological process	regulation of mRNA catabolic process	254	0.1944	10	28

GO:1903313	biological process	positive regulation of mRNA metabolic process	254	0.1944	10	28
GO:0050794	biological process	regulation of cellular process	255	0.1540	410	1612
GO:0050789	biological process	regulation of biological process	255	0.1540	457	1799
GO:0050865	biological process	regulation of cell activation	256	0.2485	2	7
GO:0050896	biological process	response to stimulus	257	0.1465	314	1488
GO:0051052	biological process	regulation of DNA metabolic process	258	0.0967	38	150
GO:0051128	biological process	regulation of cellular component organization	259	0.2848	4	10
GO:0051156	biological process	glucose 6-phosphate metabolic process	260	0.1735	2	13
GO:0051174	biological process	regulation of phosphorus metabolic process	261	0.3270	2	5
GO:0051179	biological process	localization	262	0.1198	126	708
GO:0051186	biological process	cofactor metabolic process	263	0.1765	74	366
GO:0051188	biological process	cofactor biosynthetic process	264	0.1565	67	336
GO:0006732	biological process	coenzyme metabolic process	264	0.1565	64	326
GO:0006733	biological process	oxidoreduction coenzyme metabolic process	264	0.1565	13	142
GO:0009108	biological process	coenzyme biosynthetic process	264	0.1565	60	308
GO:0051202	biological process	phytochromobilin metabolic process	265	0.1297	3	15
GO:0051246	biological process	regulation of protein metabolic process	266	0.1455	30	99
GO:0051248	biological process	negative regulation of protein metabolic process	267	0.0909	4	20
GO:0045861	biological process	negative regulation of proteolysis	267	0.0909	3	8
GO:0052548	biological process	regulation of endopeptidase activity	267	0.0909	3	8
GO:0051259	biological process	protein oligomerization	268	0.1517	56	130
GO:0051301	biological process	cell division	269	0.4140	1	2
GO:0051604	biological process	protein maturation	270	0.1973	18	66
GO:0051606	biological process	detection of stimulus	271	0.1190	1	2
GO:0051641	biological process	cellular localization	272	0.1043	43	197
GO:0051649	biological process	establishment of localization in cell	273	0.1053	43	197
GO:0051169	biological process	nuclear transport	273	0.1053	42	191

GO:0051704	biological process	multi-organism process	274	0.1300	1258	5237
GO:0051716	biological process	cellular response to stimulus	275	0.1460	179	996
GO:0051726	biological process	regulation of cell cycle	276	0.1811	17	60
GO:0051814	biological process	movement in other organism involved in symbiotic interaction	277	0.1116	36	205
GO:0051821	biological process	dissemination or transmission of organism from other organism involved in symbiotic interaction	278	0.2005	6	17
GO:0052203	biological process	modulation of catalytic activity in other organism involved in symbiotic interaction	279	0.0958	22	69
GO:0039523	biological process	suppression by virus of host RNA polymerase II activity	279	0.0958	1	2
GO:0039563	biological process	suppression by virus of host STAT1 activity	279	0.0958	3	7
GO:0039724	biological process	suppression by virus of host IKBKE activity	279	0.0958	1	2
GO:0055085	biological process	transmembrane transport	280	0.1745	2	32
GO:0055086	biological process	nucleobase-containing small molecule metabolic process	281	0.1491	239	1460
GO:0055114	biological process	oxidation-reduction process	282	0.1741	27	149
GO:0060759	biological process	regulation of response to cytokine stimulus	283	0.1536	68	225
GO:0060968	biological process	regulation of gene silencing	284	0.1938	18	96
GO:0061024	biological process	membrane organization	285	0.0989	73	262
GO:0065007	biological process	biological regulation	286	0.1553	483	1907
GO:0065008	biological process	regulation of biological quality	287	0.1559	83	391
GO:0065009	biological process	regulation of molecular function	288	0.1742	68	223
GO:0070085	biological process	glycosylation	289	0.0654	5	14
GO:0070647	biological process	protein modification by small protein conjugation or removal	290	0.1148	6	22
GO:0071103	biological process	DNA conformation change	291	0.0785	100	531
GO:0006323	biological process	DNA packaging	291	0.0785	52	278

GO:0032200	biological process	telomere organization	291	0.0785	2	31
GO:0032392	biological process	DNA geometric change	291	0.0785	5	25
GO:0071702	biological process	organic substance transport	292	0.1164	18	109
GO:0071704	biological process	organic substance metabolic process	293	0.1367	2134	10355
GO:0071705	biological process	nitrogen compound transport	294	0.1066	16	105
GO:0071824	biological process	protein-DNA complex subunit organization	295	0.1353	6	14
GO:0071840	biological process	cellular component organization or biogenesis	296	0.1241	243	994
GO:0071897	biological process	DNA biosynthetic process	297	0.1430	35	177
GO:0072330	biological process	monocarboxylic acid biosynthetic process	298	0.2038	5	21
GO:0006631	biological process	fatty acid metabolic process	298	0.2038	1	2
GO:0072521	biological process	purine-containing compound metabolic process	299	0.2333	11	57
GO:0072522	biological process	purine-containing compound biosynthetic process	300	0.1923	10	54
GO:0009119	biological process	ribonucleoside metabolic process	300	0.1923	4	13
GO:0009150	biological process	purine ribonucleotide metabolic process	300	0.1923	4	20
GO:0042278	biological process	purine nucleoside metabolic process	300	0.1923	2	5
GO:0042451	biological process	purine nucleoside biosynthetic process	300	0.1923	1	2
GO:0072524	biological process	pyridine-containing compound metabolic process	301	0.1339	13	142
GO:0072525	biological process	pyridine-containing compound biosynthetic process	302	0.1267	11	129
GO:0019674	biological process	NAD metabolic process	302	0.1267	11	129
GO:0072527	biological process	pyrimidine-containing compound metabolic process	303	0.1469	146	764
GO:0072583	biological process	clathrin-dependent endocytosis	304	0.1790	1	2
GO:0072593	biological process	reactive oxygen species metabolic process	305	0.0965	2	13
GO:0090304	biological process	nucleic acid metabolic process	306	0.1275	1542	7247
GO:0097164	biological process	ammonium ion metabolic process	307	0.2069	7	25
GO:0098003	biological process	viral tail assembly	308	0.2250	24	98

GO:0098657	biological process	import into cell	309	0.0731	10	110
GO:0099000	biological process	viral genome ejection through host cell envelope, contractile tail mechanism	310	0.2239	24	116
GO:0099002	biological process	viral genome ejection through host cell envelope, short tail mechanism	310	0.2239	10	71
GO:0099018	biological process	restriction-modification system evasion by virus	311	0.0980	1	9
GO:0099045	biological process	viral extrusion	312	0.2808	1	2
GO:0046761	biological process	viral budding from plasma membrane	312	0.2808	6	13
GO:0099046	biological process	clearance of foreign intracellular nucleic acids	313	0.2287	2	7
GO:0009617	biological process	response to bacterium	313	0.2287	17	75
GO:0009620	biological process	response to fungus	313	0.2287	1	3
GO:0052173	biological process	response to defenses of other organism involved in symbiotic interaction	313	0.2287	98	335
GO:0098542	biological process	defense response to other organism	313	0.2287	17	75
GO:1901135	biological process	carbohydrate derivative metabolic process	314	0.1538	362	2134
GO:1901136	biological process	carbohydrate derivative catabolic process	315	0.1656	131	838
GO:1901184	biological process	regulation of ERBB signaling pathway	316	0.3270	2	5
GO:1901360	biological process	organic cyclic compound metabolic process	317	0.1317	1836	8912
GO:1901361	biological process	organic cyclic compound catabolic process	318	0.1769	27	105
GO:1901362	biological process	organic cyclic compound biosynthetic process	319	0.1327	753	3307
GO:1901564	biological process	organonitrogen compound metabolic process	320	0.1482	636	3024
GO:1901565	biological process	organonitrogen compound catabolic process	321	0.1614	125	815
GO:1901566	biological process	organonitrogen compound biosynthetic process	322	0.1616	191	1003
GO:1901568	biological process	fatty acid derivative metabolic process	323	0.1750	1	2
GO:1901571	biological process	fatty acid derivative transport	324	0.1950	2	4
GO:1901576	biological process	organic substance biosynthetic process	325	0.1282	1274	5950
GO:1901605	biological process	alpha-amino acid metabolic process	326	0.1537	29	99
GO:0006520	biological process	cellular amino acid metabolic process	326	0.1537	39	138

GO:0009064	biological process	glutamine family amino acid metabolic process	326	0.1537	7	32
GO:1901657	biological process	glycosyl compound metabolic process	327	0.1539	20	138
GO:1901137	biological process	carbohydrate derivative biosynthetic process	327	0.1539	139	860
GO:1901659	biological process	glycosyl compound biosynthetic process	328	0.1940	3	10
GO:1902579	biological process	multi-organism localization	329	0.1157	111	626
GO:1902586	biological process	multi-organism intercellular transport	330	0.0901	36	205
GO:0075519	biological process	microtubule-dependent intracellular transport of viral material	330	0.0901	23	93
GO:0075606	biological process	transport of viral material towards nucleus	330	0.0901	23	93
GO:0075733	biological process	intracellular transport of virus	330	0.0901	49	227
GO:1902581	biological process	multi-organism cellular localization	330	0.0901	42	191
GO:1903311	biological process	regulation of mRNA metabolic process	331	0.1948	16	49
GO:1903320	biological process	regulation of protein modification by small protein conjugation or removal	332	0.1279	22	69
GO:0005575	cellular component	cellular component	333	0.2036	4461	17222
GO:0005576	cellular component	extracellular region	334	0.1400	67	267
GO:0009295	cellular component	nucleoid	335	0.1580	1	7
GO:0016020	cellular component	membrane	336	0.1150	193	696
GO:0019012	cellular component	virion	337	0.1398	57	261
GO:0019029	cellular component	helical viral capsid	338	0.0604	11	50
GO:0019867	cellular component	outer membrane	339	0.2223	13	51

GO:0032991	cellular component	macromolecular complex	340	0.1969	7	40
GO:0039615	cellular component	T=1 icosahedral viral capsid	341	0.0655	29	133
GO:0039617	cellular component	T=3 icosahedral viral capsid	341	0.0655	1	3
GO:0039620	cellular component	T=7 icosahedral viral capsid	341	0.0655	6	90
GO:0039621	cellular component	T=13 icosahedral viral capsid	341	0.0655	2	4
GO:0039622	cellular component	T=16 icosahedral viral capsid	341	0.0655	2	7
GO:0039623	cellular component	T=25 icosahedral viral capsid	341	0.0655	2	23
GO:0039624	cellular component	viral outer capsid	341	0.0655	3	7
GO:0039625	cellular component	viral inner capsid	341	0.0655	1	4
GO:0044172	cellular component	host cell endoplasmic reticulum-Golgi intermediate compartment	342	0.1294	7	15
GO:0030430	cellular component	host cell cytoplasm	342	0.1294	228	1016
GO:0033643	cellular component	host cell part	342	0.1294	771	3276
GO:0033646	cellular component	host intracellular part	342	0.1294	598	2535
GO:0033647	cellular component	host intracellular organelle	342	0.1294	410	1830
GO:0033648	cellular component	host intracellular membrane-bounded organelle	342	0.1294	409	1827

GO:0033650	cellular component	host cell mitochondrion	342	0.1294	1	8
GO:0033655	cellular component	host cell cytoplasm part	342	0.1294	100	284
GO:0042025	cellular component	host cell nucleus	342	0.1294	383	1752
GO:0044162	cellular component	host cell cytoplasmic vesicle membrane	342	0.1294	47	104
GO:0044165	cellular component	host cell endoplasmic reticulum	342	0.1294	2	7
GO:0044174	cellular component	host cell endosome	342	0.1294	2	4
GO:0044177	cellular component	host cell Golgi apparatus	342	0.1294	21	57
GO:0044216	cellular component	other organism cell	342	0.1294	19	79
GO:0044217	cellular component	other organism part	342	0.1294	794	3382
GO:0044220	cellular component	host cell perinuclear region of cytoplasm	342	0.1294	26	102
GO:0044279	cellular component	other organism membrane	342	0.1294	261	990
GO:0044219	cellular component	host cell plasmodesma	343	0.1058	6	13
GO:0020002	cellular component	host cell plasma membrane	343	0.1058	82	361
GO:0044156	cellular component	host cell junction	343	0.1058	11	30
GO:0044166	cellular component	host cell endoplasmic reticulum lumen	343	0.1058	1	2

GO:0044167	cellular component	host cell endoplasmic reticulum membrane	343	0.1058	26	90
GO:0044168	cellular component	host cell rough endoplasmic reticulum	343	0.1058	1	3
GO:0044175	cellular component	host cell endosome membrane	343	0.1058	25	111
GO:0044178	cellular component	host cell Golgi membrane	343	0.1058	29	106
GO:0044185	cellular component	host cell late endosome membrane	343	0.1058	2	4
GO:0044200	cellular component	host cell nuclear membrane	343	0.1058	29	122
GO:0044385	cellular component	integral to membrane of host cell	343	0.1058	50	112
GO:0044386	cellular component	integral to host endoplasmic reticulum membrane	343	0.1058	3	8
GO:0044228	cellular component	host cell surface	344	0.0170	1	3
GO:0044229	cellular component	host cell periplasmic space	345	0.1562	3	31
GO:0044094	cellular component	host cell nuclear part	345	0.1562	1	2
GO:0044383	cellular component	host chromosome	346	0.0450	1	3
GO:0044384	cellular component	host outer membrane	347	0.2015	2	25
GO:0044421	cellular component	extracellular region part	348	0.0816	15	64
GO:0005615	cellular component	extracellular space	348	0.0816	9	40

GO:0031012	cellular component	extracellular matrix	348	0.0816	6	24
GO:0044425	cellular component	membrane part	349	0.2373	3117	11330
GO:0046729	cellular component	viral procapsid	350	0.0370	1	4
GO:0098021	cellular component	viral capsid, decoration	351	0.2733	7	45
GO:0098025	cellular component	virus tail, baseplate	352	0.1198	72	343
GO:0019013	cellular component	viral nucleocapsid	352	0.1198	41	165
GO:0019028	cellular component	viral capsid	352	0.1198	708	2896
GO:0019030	cellular component	icosahedral viral capsid	352	0.1198	55	290
GO:0019033	cellular component	viral tegument	352	0.1198	56	218
GO:0036338	cellular component	viral membrane	352	0.1198	205	774
GO:0044423	cellular component	virion part	352	0.1198	1080	4429
GO:0055036	cellular component	virion membrane	352	0.1198	118	413
GO:0000150	molecular function	recombinase activity	353	0.1338	28	162
GO:0000217	molecular function	DNA secondary structure binding	354	0.2038	4	16
GO:0000287	molecular function	magnesium ion binding	355	0.1582	61	356
GO:0001067	molecular function	regulatory region nucleic acid binding	356	0.2100	1	2
GO:0001653	molecular function	peptide receptor activity	357	0.1817	3	6

GO:0001848	molecular function	complement binding	358	0.2530	2	5
GO:0001883	molecular function	purine nucleoside binding	359	0.1621	3	27
GO:0001882	molecular function	nucleoside binding	359	0.1621	13	71
GO:0032553	molecular function	ribonucleotide binding	359	0.1621	948	4728
GO:0002161	molecular function	aminoacyl-tRNA editing activity	360	0.0610	1	3
GO:0003674	molecular function	molecular function	361	0.1408	5381	25017
GO:0003676	molecular function	nucleic acid binding	362	0.1391	2430	10776
GO:0003677	molecular function	DNA binding	363	0.1440	1599	7503
GO:0003684	molecular function	damaged DNA binding	364	0.1213	3	9
GO:0003690	molecular function	double-stranded DNA binding	365	0.1100	26	116
GO:0003697	molecular function	single-stranded DNA binding	366	0.1455	95	777
GO:0003723	molecular function	RNA binding	367	0.1199	484	1683
GO:0003724	molecular function	RNA helicase activity	368	0.1190	170	511
GO:0003678	molecular function	DNA helicase activity	368	0.1190	52	559
GO:0070035	molecular function	purine NTP-dependent helicase activity	368	0.1190	70	287
GO:0003725	molecular function	double-stranded RNA binding	369	0.1087	20	84
GO:0003727	molecular function	single-stranded RNA binding	370	0.0885	2	4
GO:0003729	molecular function	mRNA binding	371	0.2080	2	6
GO:0003735	molecular function	structural constituent of ribosome	372	0.2670	1	2
GO:0003824	molecular function	catalytic activity	373	0.1404	3506	16251
GO:0003829	molecular function	beta-1,3-galactosyl-O-glycosyl-glycoprotein beta-1,6-N-acetylglucosaminyltransferase activity	374	0.0170	1	4
GO:0003830	molecular function	beta-1,4-mannosylglycoprotein 4-beta-N-acetylglucosaminyltransferase activity	375	0.0180	1	4
GO:0003899	molecular function	DNA-directed 5'-3' RNA polymerase activity	376	0.1189	106	596
GO:0003896	molecular function	DNA primase activity	376	0.1189	24	249
GO:0003968	molecular function	RNA-directed 5'-3' RNA polymerase activity	376	0.1189	362	1224

GO:0034061	molecular function	DNA polymerase activity	376	0.1189	233	825
GO:0097747	molecular function	RNA polymerase activity	376	0.1189	468	1820
GO:0003916	molecular function	DNA topoisomerase activity	377	0.1442	44	230
GO:0003917	molecular function	DNA topoisomerase type I activity	378	0.1523	11	39
GO:0061505	molecular function	DNA topoisomerase II activity	378	0.1523	31	185
GO:0003924	molecular function	GTPase activity	379	0.0887	3	10
GO:0003937	molecular function	IMP cyclohydrolase activity	380	0.1557	2	5
GO:0003933	molecular function	GTP cyclohydrolase activity	380	0.1557	6	27
GO:0003950	molecular function	NAD+ ADP-ribosyltransferase activity	381	0.4050	1	2
GO:0003952	molecular function	NAD+ synthase (glutamine-hydrolyzing) activity	382	0.0210	1	4
GO:0003964	molecular function	RNA-directed DNA polymerase activity	383	0.1734	5	13
GO:0003887	molecular function	DNA-directed DNA polymerase activity	383	0.1734	228	812
GO:0003972	molecular function	RNA ligase (ATP) activity	384	0.1500	25	129
GO:0003909	molecular function	DNA ligase activity	384	0.1500	42	247
GO:0003910	molecular function	DNA ligase (ATP) activity	384	0.1500	34	197
GO:0003911	molecular function	DNA ligase (NAD+) activity	384	0.1500	7	38
GO:0008452	molecular function	RNA ligase activity	384	0.1500	32	173
GO:0004014	molecular function	adenosylmethionine decarboxylase activity	385	0.2253	6	21
GO:0004019	molecular function	adenylosuccinate synthase activity	386	0.3830	1	19
GO:0004040	molecular function	amidase activity	387	0.1872	9	30
GO:0004045	molecular function	aminoacyl-tRNA hydrolase activity	388	0.2422	5	26
GO:0004066	molecular function	asparagine synthase (glutamine-hydrolyzing) activity	389	0.2480	4	17
GO:0004089	molecular function	carbonate dehydratase activity	390	0.0160	1	14
GO:0004132	molecular function	dCMP deaminase activity	391	0.1352	15	84
GO:0004000	molecular function	adenosine deaminase activity	391	0.1352	1	2
GO:0019238	molecular function	cyclohydrolase activity	391	0.1352	8	32

GO:0004150	molecular function	dihydroneopterin aldolase activity	392	0.1380	1	3
GO:0004170	molecular function	dUTP diphosphatase activity	393	0.1125	34	154
GO:0004176	molecular function	ATP-dependent peptidase activity	394	0.0665	2	6
GO:0004180	molecular function	carboxypeptidase activity	395	0.0532	2	9
GO:0008235	molecular function	metalloexopeptidase activity	395	0.0532	1	7
GO:0070008	molecular function	serine-type exopeptidase activity	395	0.0532	3	6
GO:0004222	molecular function	metalloendopeptidase activity	396	0.1425	15	54
GO:0004345	molecular function	glucose-6-phosphate dehydrogenase activity	397	0.0170	1	11
GO:0004386	molecular function	helicase activity	398	0.1321	360	1656
GO:0004392	molecular function	heme oxygenase (decyclizing) activity	399	0.2850	1	5
GO:0004482	molecular function	mRNA (guanine-N7-)-methyltransferase activity	400	0.1140	51	195
GO:0004484	molecular function	mRNA guanylyltransferase activity	401	0.1167	13	50
GO:0008193	molecular function	tRNA guanylyltransferase activity	401	0.1167	3	13
GO:0004497	molecular function	monooxygenase activity	402	0.2890	4	8
GO:0004514	molecular function	nicotinate-nucleotide diphosphorylase (carboxylating) activity	403	0.1373	10	125
GO:0004518	molecular function	nuclease activity	404	0.1566	605	2349
GO:0042578	molecular function	phosphoric ester hydrolase activity	404	0.1566	53	226
GO:0004532	molecular function	exoribonuclease activity	405	0.1595	6	13
GO:0004521	molecular function	endoribonuclease activity	405	0.1595	30	131
GO:0004523	molecular function	RNA-DNA hybrid ribonuclease activity	405	0.1595	19	88
GO:0008408	molecular function	3'-5' exonuclease activity	405	0.1595	38	144
GO:0016796	molecular function	exonuclease activity, active with either ribo- or deoxyribonucleic acids and producing 5'-phosphomonoesters	405	0.1595	10	32
GO:0016891	molecular function	endoribonuclease activity, producing 5'-phosphomonoesters	405	0.1595	27	125
GO:0032296	molecular function	double-stranded RNA-specific ribonuclease activity	405	0.1595	8	37

GO:0004536	molecular function	deoxyribonuclease activity	406	0.1598	108	391
GO:0004519	molecular function	endonuclease activity	406	0.1598	446	1702
GO:0004527	molecular function	exonuclease activity	406	0.1598	107	390
GO:0004540	molecular function	ribonuclease activity	406	0.1598	36	144
GO:0004550	molecular function	nucleoside diphosphate kinase activity	407	0.1308	1	2
GO:0004138	molecular function	deoxyguanosine kinase activity	407	0.1308	1	2
GO:0004797	molecular function	thymidine kinase activity	407	0.1308	39	191
GO:0019136	molecular function	deoxynucleoside kinase activity	407	0.1308	40	193
GO:0019206	molecular function	nucleoside kinase activity	407	0.1308	48	220
GO:0004553	molecular function	hydrolase activity, hydrolyzing O-glycosyl compounds	408	0.1213	104	483
GO:0016799	molecular function	hydrolase activity, hydrolyzing N-glycosyl compounds	408	0.1213	19	92
GO:0019104	molecular function	DNA N-glycosylase activity	408	0.1213	13	54
GO:0004568	molecular function	chitinase activity	409	0.1945	6	18
GO:0004588	molecular function	orotate phosphoribosyltransferase activity	410	0.1380	1	2
GO:0004597	molecular function	peptide-aspartate beta-dioxygenase activity	411	0.3048	4	21
GO:0004602	molecular function	glutathione peroxidase activity	412	0.0260	1	4
GO:0004609	molecular function	phosphatidylserine decarboxylase activity	413	0.2984	1	3
GO:0016831	molecular function	carboxy-lyase activity	413	0.2984	8	29
GO:0016832	molecular function	aldehyde-lyase activity	413	0.2984	8	46
GO:0004616	molecular function	phosphogluconate dehydrogenase (decarboxylating) activity	414	0.1735	2	13
GO:0004623	molecular function	phospholipase A2 activity	415	0.1950	2	4
GO:0004629	molecular function	phospholipase C activity	416	0.0130	1	3
GO:0004639	molecular function	phosphoribosylaminoimidazolesuccinocarboxamide synthase activity	417	0.2885	4	13

GO:0004643	molecular function	phosphoribosylaminoimidazolecarboxamide formyltransferase activity	418	0.2117	2	5
GO:0003864	molecular function	3-methyl-2-oxobutanoate hydroxymethyltransferase activity	418	0.2117	1	4
GO:0004372	molecular function	glycine hydroxymethyltransferase activity	418	0.2117	1	2
GO:0004649	molecular function	poly(ADP-ribose) glycohydrolase activity	419	0.1823	3	7
GO:0004657	molecular function	proline dehydrogenase activity	420	0.0180	1	9
GO:0004666	molecular function	prostaglandin-endoperoxide synthase activity	421	0.1750	1	2
GO:0004674	molecular function	protein serine/threonine kinase activity	422	0.1061	21	76
GO:0004672	molecular function	protein kinase activity	422	0.1061	86	391
GO:0004722	molecular function	protein serine/threonine phosphatase activity	423	0.0433	3	9
GO:0004788	molecular function	thiamine diphosphokinase activity	424	0.3652	1	2
GO:0003848	molecular function	2-amino-4-hydroxy-6-hydroxymethyldihydropteridine diphosphokinase activity	424	0.3652	1	3
GO:0004749	molecular function	ribose phosphate diphosphokinase activity	424	0.3652	11	100
GO:0004803	molecular function	transposase activity	425	0.2166	10	26
GO:0004814	molecular function	arginine-tRNA ligase activity	426	0.0070	1	3
GO:0004816	molecular function	asparagine-tRNA ligase activity	427	0.0720	1	2
GO:0004822	molecular function	isoleucine-tRNA ligase activity	428	0.0610	1	3
GO:0004825	molecular function	methionine-tRNA ligase activity	429	0.1090	1	2
GO:0004857	molecular function	enzyme inhibitor activity	430	0.2151	4	17
GO:0030234	molecular function	enzyme regulator activity	430	0.2151	14	67
GO:0061134	molecular function	peptidase regulator activity	430	0.2151	2	12
GO:0004896	molecular function	cytokine receptor activity	431	0.1392	3	6
GO:0004930	molecular function	G-protein coupled receptor activity	431	0.1392	19	59
GO:0005102	molecular function	receptor binding	432	0.1540	15	42
GO:0005198	molecular function	structural molecule activity	433	0.1119	592	2423

GO:0005215	molecular function	transporter activity	434	0.1943	87	293
GO:0005488	molecular function	binding	435	0.1383	3383	15711
GO:0005506	molecular function	iron ion binding	436	0.1421	38	144
GO:0005507	molecular function	copper ion binding	437	0.1670	4	22
GO:0005509	molecular function	calcium ion binding	438	0.1555	2	5
GO:0005515	molecular function	protein binding	439	0.1292	118	440
GO:0005521	molecular function	lamin binding	440	0.2016	5	13
GO:0008061	molecular function	chitin binding	441	0.1472	25	78
GO:0008079	molecular function	translation termination factor activity	442	0.1807	1	5
GO:0003743	molecular function	translation initiation factor activity	442	0.1807	12	35
GO:0003746	molecular function	translation elongation factor activity	442	0.1807	11	38
GO:0008081	molecular function	phosphoric diester hydrolase activity	443	0.1494	5	26
GO:0004721	molecular function	phosphoprotein phosphatase activity	443	0.1494	26	109
GO:0016791	molecular function	phosphatase activity	443	0.1494	48	200
GO:0008092	molecular function	cytoskeletal protein binding	444	0.0866	5	11
GO:0008094	molecular function	DNA-dependent ATPase activity	445	0.1435	66	465
GO:0008134	molecular function	transcription factor binding	446	0.1108	4	10
GO:0008135	molecular function	translation factor activity, RNA binding	447	0.1578	24	78
GO:0008138	molecular function	protein tyrosine/serine/threonine phosphatase activity	448	0.1329	17	77
GO:0004725	molecular function	protein tyrosine phosphatase activity	448	0.1329	19	79
GO:0008144	molecular function	drug binding	449	0.1229	971	4796
GO:0008172	molecular function	S-methyltransferase activity	450	0.0380	1	2
GO:0008174	molecular function	mRNA methyltransferase activity	451	0.1092	87	351
GO:0008194	molecular function	UDP-glycosyltransferase activity	452	0.0175	2	8
GO:0008199	molecular function	ferric iron binding	453	0.1207	3	14
GO:0008233	molecular function	peptidase activity	454	0.1365	410	1470

GO:0008234	molecular function	cysteine-type peptidase activity	455	0.1197	258	798
GO:0008238	molecular function	exopeptidase activity	456	0.1444	12	30
GO:0004175	molecular function	endopeptidase activity	456	0.1444	273	931
GO:0008237	molecular function	metallopeptidase activity	456	0.1444	19	68
GO:0070011	molecular function	peptidase activity, acting on L-amino acid peptides	456	0.1444	348	1195
GO:0008242	molecular function	omega peptidase activity	457	0.1982	6	13
GO:0004177	molecular function	aminopeptidase activity	457	0.1982	1	2
GO:0008252	molecular function	nucleotidase activity	458	0.1955	11	45
GO:0008270	molecular function	zinc ion binding	459	0.1564	147	788
GO:0008309	molecular function	double-stranded DNA exodeoxyribonuclease activity	460	0.0390	1	5
GO:0008324	molecular function	cation transmembrane transporter activity	461	0.1520	2	4
GO:0015075	molecular function	ion transmembrane transporter activity	461	0.1520	49	108
GO:0015077	molecular function	monovalent inorganic cation transmembrane transporter activity	461	0.1520	1	2
GO:0022804	molecular function	active transmembrane transporter activity	461	0.1520	1	6
GO:0008373	molecular function	sialyltransferase activity	462	0.0850	1	3
GO:0008374	molecular function	O-acyltransferase activity	463	0.1695	2	5
GO:0008409	molecular function	5'-3' exonuclease activity	464	0.0390	1	5
GO:0008417	molecular function	fucosyltransferase activity	465	0.0563	4	11
GO:0008446	molecular function	GDP-mannose 4,6-dehydratase activity	466	0.0110	1	22
GO:0008565	molecular function	protein transporter activity	467	0.0803	6	30
GO:0008821	molecular function	crossover junction endodeoxyribonuclease activity	468	0.0449	3	33
GO:0003906	molecular function	DNA-(apurinic or apyrimidinic site) lyase activity	468	0.0449	2	6
GO:0004531	molecular function	deoxyribonuclease II activity	468	0.0449	1	4
GO:0016836	molecular function	hydro-lyase activity	468	0.0449	2	36
GO:0016889	molecular function	endodeoxyribonuclease activity, producing 3'-phosphomonoesters	468	0.0449	4	37

GO:0016892	molecular function	endoribonuclease activity, producing 3'-phosphomonoesters	468	0.0449	3	6
GO:0008829	molecular function	dCTP deaminase activity	469	0.0756	5	24
GO:0008833	molecular function	deoxyribonuclease IV (phage-T4-induced) activity	470	0.2723	18	73
GO:0008879	molecular function	glucose-1-phosphate thymidylyltransferase activity	471	0.0290	1	4
GO:0008889	molecular function	glycerophosphodiester phosphodiesterase activity	472	0.2208	4	23
GO:0008933	molecular function	lytic transglycosylase activity	473	0.1900	1	2
GO:0003796	molecular function	lysozyme activity	473	0.1900	67	353
GO:0008745	molecular function	N-acetylmuramoyl-L-alanine amidase activity	473	0.1900	47	422
GO:0008962	molecular function	phosphatidylglycerophosphatase activity	474	0.1740	1	2
GO:0008998	molecular function	ribonucleoside-triphosphate reductase activity	475	0.1129	19	112
GO:0061731	molecular function	ribonucleoside-diphosphate reductase activity	475	0.1129	33	222
GO:0009007	molecular function	site-specific DNA-methyltransferase (adenine-specific) activity	476	0.1102	39	239
GO:0003886	molecular function	DNA (cytosine-5-)-methyltransferase activity	476	0.1102	5	28
GO:0015667	molecular function	site-specific DNA-methyltransferase (cytosine-N4-specific) activity	476	0.1102	5	17
GO:0009008	molecular function	DNA-methyltransferase activity	477	0.1276	49	284
GO:0009055	molecular function	electron carrier activity	478	0.1781	55	318
GO:0009378	molecular function	four-way junction helicase activity	479	0.1051	1	2
GO:0004003	molecular function	ATP-dependent DNA helicase activity	479	0.1051	8	76
GO:0010181	molecular function	FMN binding	480	0.1880	4	36
GO:0015036	molecular function	disulfide oxidoreductase activity	481	0.1351	39	206
GO:0016670	molecular function	oxidoreductase activity, acting on a sulfur group of donors, oxygen as acceptor	481	0.1351	22	85
GO:0015318	molecular function	inorganic solute uptake transmembrane transporter activity	482	0.1535	50	114
GO:0015666	molecular function	restriction endodeoxyribonuclease activity	483	0.1999	2	7

GO:0004520	molecular function	endodeoxyribonuclease activity	483	0.1999	99	355
GO:0004529	molecular function	exodeoxyribonuclease activity	483	0.1999	4	19
GO:0008855	molecular function	exodeoxyribonuclease VII activity	483	0.1999	3	14
GO:0016888	molecular function	endodeoxyribonuclease activity, producing 5'-phosphomonoesters	483	0.1999	86	289
GO:0016893	molecular function	endonuclease activity, active with either ribo- or deoxyribonucleic acids and producing 5'-phosphomonoesters	483	0.1999	112	412
GO:0015929	molecular function	hexosaminidase activity	484	0.2740	1	2
GO:0015932	molecular function	nucleobase-containing compound transmembrane transporter activity	485	0.1658	9	32
GO:0016209	molecular function	antioxidant activity	486	0.1050	8	31
GO:0016229	molecular function	steroid dehydrogenase activity	487	0.1166	5	26
GO:0016298	molecular function	lipase activity	488	0.1343	3	7
GO:0016410	molecular function	N-acyltransferase activity	489	0.2057	7	20
GO:0008080	molecular function	N-acetyltransferase activity	489	0.2057	2	5
GO:0016407	molecular function	acetyltransferase activity	489	0.2057	4	13
GO:0016747	molecular function	transferase activity, transferring acyl groups other than amino-acyl groups	489	0.2057	15	42
GO:0016412	molecular function	serine O-acyltransferase activity	490	0.2670	1	3
GO:0016413	molecular function	O-acetyltransferase activity	490	0.2670	1	3
GO:0016491	molecular function	oxidoreductase activity	491	0.1476	344	1780
GO:0016597	molecular function	amino acid binding	492	0.1240	1	3
GO:0016614	molecular function	oxidoreductase activity, acting on CH-OH group of donors	493	0.1084	16	78
GO:0016616	molecular function	oxidoreductase activity, acting on the CH-OH group of donors, NAD or NADP as acceptor	494	0.1143	12	65

GO:0016627	molecular function	oxidoreductase activity, acting on the CH-CH group of donors	495	0.1590	4	17
GO:0016628	molecular function	oxidoreductase activity, acting on the CH-CH group of donors, NAD or NADP as acceptor	496	0.2470	1	2
GO:0016636	molecular function	oxidoreductase activity, acting on the CH-CH group of donors, iron-sulfur protein as acceptor	497	0.1297	3	15
GO:0016638	molecular function	oxidoreductase activity, acting on the CH-NH2 group of donors	498	0.4450	1	3
GO:0016645	molecular function	oxidoreductase activity, acting on the CH-NH group of donors	499	0.1661	43	160
GO:0016646	molecular function	oxidoreductase activity, acting on the CH-NH group of donors, NAD or NADP as acceptor	500	0.1687	42	151
GO:0004146	molecular function	dihydrofolate reductase activity	500	0.1687	40	143
GO:0016651	molecular function	oxidoreductase activity, acting on NAD(P)H	501	0.1843	3	10
GO:0016657	molecular function	oxidoreductase activity, acting on NAD(P)H, nitrogenous group as acceptor	502	0.1622	2	8
GO:0003954	molecular function	NADH dehydrogenase activity	502	0.1622	1	2
GO:0016655	molecular function	oxidoreductase activity, acting on NAD(P)H, quinone or similar compound as acceptor	502	0.1622	1	2
GO:0016661	molecular function	oxidoreductase activity, acting on other nitrogenous compounds as donors	503	0.3140	1	3
GO:0016667	molecular function	oxidoreductase activity, acting on a sulfur group of donors	504	0.1435	61	291
GO:0016679	molecular function	oxidoreductase activity, acting on diphenols and related substances as donors	505	0.0805	2	19
GO:0016684	molecular function	oxidoreductase activity, acting on peroxide as acceptor	506	0.1303	3	10
GO:0016701	molecular function	oxidoreductase activity, acting on single donors with incorporation of molecular oxygen	507	0.4385	2	4

GO:0016705	molecular function	oxidoreductase activity, acting on paired donors, with incorporation or reduction of molecular oxygen	508	0.1332	40	156
GO:0016706	molecular function	oxidoreductase activity, acting on paired donors, with incorporation or reduction of molecular oxygen, 2-oxoglutarate as one donor, and incorporation of one atom each of oxygen into both donors	509	0.2340	6	28
GO:0016714	molecular function	oxidoreductase activity, acting on paired donors, with incorporation or reduction of molecular oxygen, reduced pteridine as one donor, and incorporation of one atom of oxygen	510	0.1580	1	2
GO:0016717	molecular function	oxidoreductase activity, acting on paired donors, with oxidation of a pair of donors resulting in the reduction of molecular oxygen to two molecules of water	511	0.0700	1	2
GO:0016721	molecular function	oxidoreductase activity, acting on superoxide radicals as acceptor	512	0.0898	5	21
GO:0016722	molecular function	oxidoreductase activity, oxidizing metal ions	513	0.1550	1	2
GO:0016725	molecular function	oxidoreductase activity, acting on CH or CH2 groups	514	0.1108	52	334
GO:0016740	molecular function	transferase activity	515	0.1233	1378	5826
GO:0016741	molecular function	transferase activity, transferring one-carbon groups	516	0.1317	340	1709
GO:0016742	molecular function	hydroxymethyl-, formyl- and related transferase activity	517	0.1313	5	15
GO:0008168	molecular function	methyltransferase activity	517	0.1313	334	1691
GO:0008170	molecular function	N-methyltransferase activity	517	0.1313	103	452
GO:0008171	molecular function	O-methyltransferase activity	517	0.1313	40	144
GO:0008173	molecular function	RNA methyltransferase activity	517	0.1313	89	356

GO:0008757	molecular function	S-adenosylmethionine-dependent methyltransferase activity	517	0.1313	136	635
GO:0016743	molecular function	carboxyl- or carbamoyltransferase activity	518	0.1240	1	3
GO:0016746	molecular function	transferase activity, transferring acyl groups	519	0.1932	17	46
GO:0016757	molecular function	transferase activity, transferring glycosyl groups	520	0.1560	47	247
GO:0016763	molecular function	transferase activity, transferring pentosyl groups	521	0.1586	18	160
GO:0016758	molecular function	transferase activity, transferring hexosyl groups	521	0.1586	16	49
GO:0016765	molecular function	transferase activity, transferring alkyl or aryl (other than methyl) groups	522	0.0640	1	10
GO:0016769	molecular function	transferase activity, transferring nitrogenous groups	523	0.1923	3	29
GO:0016772	molecular function	transferase activity, transferring phosphorus-containing groups	524	0.1166	986	3864
GO:0016773	molecular function	phosphotransferase activity, alcohol group as acceptor	525	0.1044	89	402
GO:0016301	molecular function	kinase activity	525	0.1044	195	838
GO:0016775	molecular function	phosphotransferase activity, nitrogenous group as acceptor	526	0.1190	1	2
GO:0016776	molecular function	phosphotransferase activity, phosphate group as acceptor	527	0.1581	7	17
GO:0016778	molecular function	diphosphotransferase activity	528	0.2016	13	105
GO:0016779	molecular function	nucleotidyltransferase activity	529	0.1148	776	2918
GO:0016780	molecular function	phosphotransferase activity, for other substituted phosphate groups	530	0.1590	1	3
GO:0016787	molecular function	hydrolase activity	531	0.1447	1867	8429
GO:0016788	molecular function	hydrolase activity, acting on ester bonds	532	0.1604	703	2742
GO:0016798	molecular function	hydrolase activity, acting on glycosyl bonds	533	0.1703	127	586
GO:0016801	molecular function	hydrolase activity, acting on ether bonds	534	0.2670	1	2

GO:0016810	molecular function	hydrolase activity, acting on carbon-nitrogen (but not peptide) bonds	535	0.1383	89	604
GO:0016813	molecular function	hydrolase activity, acting on carbon-nitrogen (but not peptide) bonds, in linear amidines	536	0.0960	1	4
GO:0016814	molecular function	hydrolase activity, acting on carbon-nitrogen (but not peptide) bonds, in cyclic amidines	537	0.1316	29	142
GO:0016811	molecular function	hydrolase activity, acting on carbon-nitrogen (but not peptide) bonds, in linear amides	537	0.1316	56	452
GO:0016817	molecular function	hydrolase activity, acting on acid anhydrides	538	0.1244	621	2833
GO:0016829	molecular function	lyase activity	539	0.2158	37	188
GO:0016830	molecular function	carbon-carbon lyase activity	540	0.2085	16	75
GO:0016835	molecular function	carbon-oxygen lyase activity	541	0.0760	3	41
GO:0016838	molecular function	carbon-oxygen lyase activity, acting on phosphates	542	0.2010	1	5
GO:0016853	molecular function	isomerase activity	543	0.1439	53	253
GO:0016854	molecular function	racemase and epimerase activity	544	0.0700	1	3
GO:0016859	molecular function	cis-trans isomerase activity	545	0.0360	1	2
GO:0016860	molecular function	intramolecular oxidoreductase activity	546	0.2580	1	2
GO:0016866	molecular function	intramolecular transferase activity	547	0.0240	1	4
GO:0016874	molecular function	ligase activity	548	0.1516	126	675
GO:0016875	molecular function	ligase activity, forming carbon-oxygen bonds	549	0.1865	14	48
GO:0016879	molecular function	ligase activity, forming carbon-nitrogen bonds	549	0.1865	19	101
GO:0016880	molecular function	acid-ammonia (or amide) ligase activity	550	0.0170	1	3
GO:0016881	molecular function	acid-amino acid ligase activity	551	0.2090	6	18
GO:0016884	molecular function	carbon-nitrogen ligase activity, with glutamine as amido-N-donor	551	0.2090	9	40
GO:0016882	molecular function	cyclo-ligase activity	552	0.0430	1	13
GO:0016886	molecular function	ligase activity, forming phosphoric ester bonds	553	0.1417	74	420
GO:0016887	molecular function	ATPase activity	554	0.1284	186	894

GO:0016462	molecular function	pyrophosphatase activity	554	0.1284	503	2400
GO:0016818	molecular function	hydrolase activity, acting on acid anhydrides, in phosphorus-containing anhydrides	554	0.1284	600	2742
GO:0017111	molecular function	nucleoside-triphosphatase activity	554	0.1284	467	2242
GO:0042623	molecular function	ATPase activity, coupled	554	0.1284	130	682
GO:0016894	molecular function	endonuclease activity, active with either ribo- or deoxyribonucleic acids and producing 3'-phosphomonoesters	555	0.0541	7	43
GO:0016903	molecular function	oxidoreductase activity, acting on the aldehyde or oxo group of donors	556	0.1580	1	2
GO:0016977	molecular function	chitosanase activity	557	0.0250	1	5
GO:0016987	molecular function	sigma factor activity	558	0.2228	4	14
GO:0016997	molecular function	alpha-sialidase activity	559	0.0830	10	36
GO:0017060	molecular function	3-galactosyl-N-acetylglucosaminide 4-alpha-L-fucosyltransferase activity	560	0.1110	1	2
GO:0017076	molecular function	purine nucleotide binding	561	0.1223	944	4692
GO:0017171	molecular function	serine hydrolase activity	562	0.1254	63	301
GO:0019001	molecular function	guanyl nucleotide binding	563	0.2133	3	27
GO:0019107	molecular function	myristoyltransferase activity	564	0.2574	5	15
GO:0019200	molecular function	carbohydrate kinase activity	565	0.1400	1	7
GO:0019205	molecular function	nucleobase-containing compound kinase activity	566	0.1303	55	237
GO:0019239	molecular function	deaminase activity	567	0.1425	21	110
GO:0019783	molecular function	ubiquitin-like protein-specific protease activity	568	0.1557	14	48
GO:0004197	molecular function	cysteine-type endopeptidase activity	568	0.1557	192	566
GO:0019784	molecular function	NEDD8-specific protease activity	569	0.0647	3	16
GO:0019787	molecular function	ubiquitin-like protein transferase activity	570	0.0653	7	27
GO:0019838	molecular function	growth factor binding	571	0.0950	2	11
GO:0019842	molecular function	vitamin binding	572	0.1148	33	163

GO:0019899	molecular function	enzyme binding	573	0.1065	2	5
GO:0019904	molecular function	protein domain specific binding	574	0.1798	18	62
GO:0019955	molecular function	cytokine binding	575	0.1475	4	17
GO:0020037	molecular function	heme binding	576	0.1740	7	19
GO:0022803	molecular function	passive transmembrane transporter activity	577	0.2062	66	215
GO:0022838	molecular function	substrate-specific channel activity	578	0.1537	47	104
GO:0022857	molecular function	transmembrane transporter activity	579	0.2028	81	263
GO:0030165	molecular function	PDZ domain binding	580	0.1590	1	2
GO:0030246	molecular function	carbohydrate binding	581	0.1239	35	130
GO:0030337	molecular function	DNA polymerase processivity factor activity	582	0.0607	10	50
GO:0030545	molecular function	receptor regulator activity	583	0.1198	11	33
GO:0030554	molecular function	adenyl nucleotide binding	584	0.1220	941	4665
GO:0030597	molecular function	RNA glycosylase activity	585	0.0750	1	12
GO:0031072	molecular function	heat shock protein binding	586	0.0150	2	7
GO:0031127	molecular function	alpha-(1,2)-fucosyltransferase activity	587	0.0450	2	6
GO:0031419	molecular function	cobalamin binding	588	0.0060	1	23
GO:0031423	molecular function	hexon binding	589	0.0318	6	46
GO:0033170	molecular function	protein-DNA loading ATPase activity	590	0.0720	1	2
GO:0034290	molecular function	holin activity	591	0.3359	19	111
GO:0035639	molecular function	purine ribonucleoside triphosphate binding	592	0.1223	944	4692
GO:0036094	molecular function	small molecule binding	593	0.1200	1191	5881
GO:0039660	molecular function	structural constituent of virion	594	0.1043	4	13
GO:0042083	molecular function	5,10-methylenetetrahydrofolate-dependent methyltransferase activity	595	0.1640	73	445
GO:0042301	molecular function	phosphate ion binding	596	0.0340	1	15
GO:0042624	molecular function	ATPase activity, uncoupled	597	0.1204	36	104
GO:0042802	molecular function	identical protein binding	598	0.0750	5	29

GO:0043167	molecular function	ion binding	599	0.1305	1409	7029
GO:0043168	molecular function	anion binding	600	0.1251	1032	5223
GO:0043169	molecular function	cation binding	601	0.1424	462	2122
GO:0043177	molecular function	organic acid binding	602	0.1067	29	113
GO:0043364	molecular function	catalysis of free radical formation	603	0.2375	15	90
GO:0043565	molecular function	sequence-specific DNA binding	604	0.1725	239	929
GO:0044389	molecular function	ubiquitin-like protein ligase binding	605	0.0110	1	3
GO:0045156	molecular function	electron transporter, transferring electrons within the cyclic electron transport pathway of photosynthesis activity	606	0.1569	12	90
GO:0046527	molecular function	glucosyltransferase activity	607	0.0980	1	9
GO:0046812	molecular function	host cell surface binding	608	0.0572	14	53
GO:0046872	molecular function	metal ion binding	609	0.1426	461	2119
GO:0046906	molecular function	tetrapyrrole binding	610	0.1530	8	42
GO:0046914	molecular function	transition metal ion binding	611	0.1534	192	969
GO:0046982	molecular function	protein heterodimerization activity	612	0.0928	4	23
GO:0046983	molecular function	protein dimerization activity	613	0.1159	47	185
GO:0047429	molecular function	nucleoside-triphosphate diphosphatase activity	614	0.1141	36	158
GO:0048029	molecular function	monosaccharide binding	615	0.1060	28	110
GO:0048037	molecular function	cofactor binding	616	0.1725	162	840
GO:0048256	molecular function	flap endonuclease activity	617	0.1190	10	26
GO:0050145	molecular function	nucleoside phosphate kinase activity	618	0.1563	6	15
GO:0050660	molecular function	flavin adenine dinucleotide binding	619	0.1544	44	311
GO:0050661	molecular function	NADP binding	619	0.1544	22	92
GO:0050662	molecular function	coenzyme binding	619	0.1544	121	646
GO:0051287	molecular function	NAD binding	619	0.1544	10	41
GO:0050797	molecular function	thymidylate synthase (FAD) activity	620	0.1623	39	295

GO:0004799	molecular function	thymidylate synthase activity	620	0.1623	34	150
GO:0050897	molecular function	cobalt ion binding	621	0.1297	3	15
GO:0051020	molecular function	GTPase binding	622	0.2020	1	2
GO:0051082	molecular function	unfolded protein binding	623	0.0603	4	11
GO:0051213	molecular function	dioxygenase activity	624	0.2838	9	34
GO:0051219	molecular function	phosphoprotein binding	625	0.2510	1	4
GO:0051537	molecular function	2 iron, 2 sulfur cluster binding	626	0.2886	5	17
GO:0051539	molecular function	4 iron, 4 sulfur cluster binding	626	0.2886	16	92
GO:0051540	molecular function	metal cluster binding	627	0.2456	32	150
GO:0052689	molecular function	carboxylic ester hydrolase activity	628	0.1823	10	40
GO:0052796	molecular function	exo-alpha-(2->8)-sialidase activity	629	0.1510	1	2
GO:0052794	molecular function	exo-alpha-(2->3)-sialidase activity	629	0.1510	1	2
GO:0052795	molecular function	exo-alpha-(2->6)-sialidase activity	629	0.1510	1	2
GO:0060089	molecular function	molecular transducer activity	630	0.1027	20	61
GO:0060703	molecular function	deoxyribonuclease inhibitor activity	631	0.3930	2	5
GO:0061783	molecular function	peptidoglycan muralytic activity	632	0.1621	115	777
GO:0070001	molecular function	aspartic-type peptidase activity	633	0.2353	6	16
GO:0070279	molecular function	vitamin B6 binding	634	0.2035	4	30
GO:0070403	molecular function	NAD+ binding	635	0.1720	6	26
GO:0070548	molecular function	L-glutamine aminotransferase activity	636	0.1675	2	8
GO:0070566	molecular function	adenylyltransferase activity	637	0.1668	8	46
GO:0070568	molecular function	guanylyltransferase activity	638	0.1373	16	63
GO:0070851	molecular function	growth factor receptor binding	639	0.1736	2	5
GO:0001664	molecular function	G-protein coupled receptor binding	639	0.1736	4	11
GO:0005125	molecular function	cytokine activity	639	0.1736	4	11
GO:0005126	molecular function	cytokine receptor binding	639	0.1736	4	11
GO:0008083	molecular function	growth factor activity	639	0.1736	7	22

GO:0071949	molecular function	FAD binding	640	0.2140	1	3
GO:0097159	molecular function	organic cyclic compound binding	641	0.1373	2999	14020
GO:0097367	molecular function	carbohydrate derivative binding	642	0.1237	988	4867
GO:0098518	molecular function	polynucleotide phosphatase activity	643	0.1861	8	29
GO:0098772	molecular function	molecular function regulator	644	0.1284	26	102
GO:0101005	molecular function	ubiquitinyl hydrolase activity	645	0.2137	11	32
GO:1901265	molecular function	nucleoside phosphate binding	646	0.1199	1148	5694
GO:1901363	molecular function	heterocyclic compound binding	647	0.1373	2999	14020
GO:1901505	molecular function	carbohydrate derivative transporter activity	648	0.1658	9	32
GO:1990817	molecular function	RNA adenylyltransferase activity	649	0.1190	3	18
GO:1990837	molecular function	sequence-specific double-stranded DNA binding	650	0.0612	14	71
GO:0030983	molecular function	mismatched DNA binding	650	0.0612	3	7
