# Supplementary figures and images for: Conserved Secondary Structures in Viral mRNAs
Source: Viruses. 2019 Apr 29;11(5):401. doi: 10.3390/v11050401 (PMC6563262; doi:10.3390/v11050401)

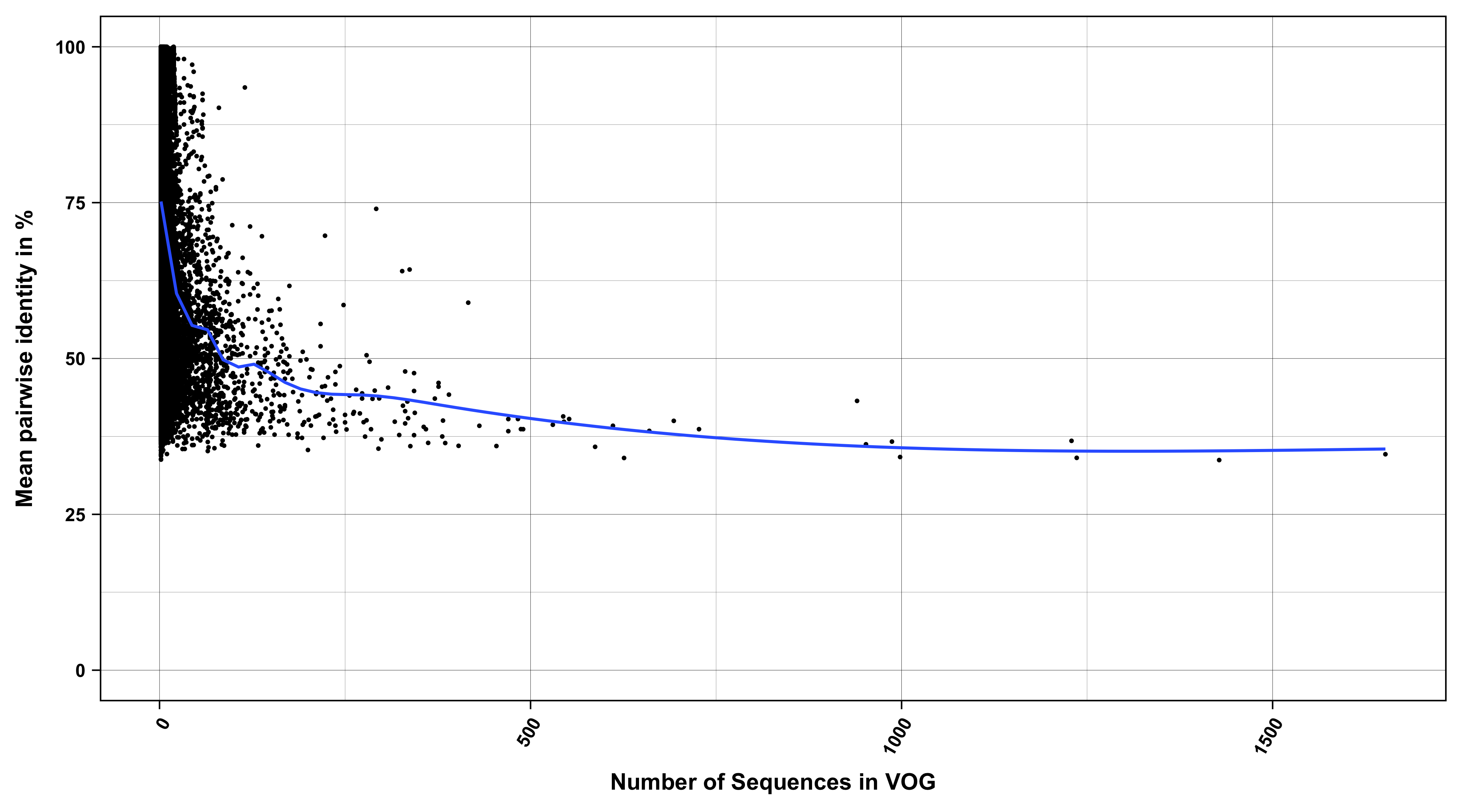

Supplement: Supplementary file 1 [file viruses-11-00401-s001.zip › supplement/Fig_S2.png]
